# Evidence for genetic association of *RORB *with bipolar disorder

**DOI:** 10.1186/1471-244X-9-70

**Published:** 2009-11-12

**Authors:** Casey L McGrath, Stephen J Glatt, Pamela Sklar, Helen Le-Niculescu, Ronald Kuczenski, Alysa E Doyle, Joseph Biederman, Eric Mick, Stephen V Faraone, Alexander B Niculescu, Ming T Tsuang

**Affiliations:** 1 Center for Human Genetic Research, Massachusetts General Hospital, Boston, MA, USA; 2 Department of Biology, Indiana University, Bloomington, IN, USA; 3 Department of Psychiatry, SUNY Upstate Medical University, Syracuse, NY, USA; 4 Laboratory of Neurophenomics, Department of Psychiatry, Indiana University School of Medicine, Indianapolis, IN, USA; 5 Department of Psychiatry, UC San Diego, La Jolla, CA, USA; 6 Pediatric Psychopharmacology Unit, Massachusetts General Hospital; Psychiatric Psychopharmacology Unit, Massachusetts General Hospital, Harvard Medical School, Boston, MA, USA

## Abstract

**Background:**

Bipolar disorder, particularly in children, is characterized by rapid cycling and switching, making circadian clock genes plausible molecular underpinnings for bipolar disorder. We previously reported work establishing mice lacking the clock gene D-box binding protein (*DBP*) as a stress-reactive genetic animal model of bipolar disorder. Microarray studies revealed that expression of two closely related clock genes, *RAR*-related orphan receptors alpha (*RORA*) and beta (*RORB*), was altered in these mice. These retinoid-related receptors are involved in a number of pathways including neurogenesis, stress response, and modulation of circadian rhythms. Here we report association studies between bipolar disorder and single-nucleotide polymorphisms (SNPs) in *RORA *and *RORB*.

**Methods:**

We genotyped 355 *RORA *and *RORB *SNPs in a pediatric cohort consisting of a family-based sample of 153 trios and an independent, non-overlapping case-control sample of 152 cases and 140 controls. Bipolar disorder in children and adolescents is characterized by increased stress reactivity and frequent episodes of shorter duration; thus our cohort provides a potentially enriched sample for identifying genes involved in cycling and switching.

**Results:**

We report that four intronic *RORB *SNPs showed positive associations with the pediatric bipolar phenotype that survived Bonferroni correction for multiple comparisons in the case-control sample. Three *RORB *haplotype blocks implicating an additional 11 SNPs were also associated with the disease in the case-control sample. However, these significant associations were not replicated in the sample of trios. There was no evidence for association between pediatric bipolar disorder and any *RORA *SNPs or haplotype blocks after multiple-test correction. In addition, we found no strong evidence for association between the age-at-onset of bipolar disorder with any *RORA *or *RORB *SNPs.

**Conclusion:**

Our findings suggest that clock genes in general and *RORB *in particular may be important candidates for further investigation in the search for the molecular basis of bipolar disorder.

## Background

Bipolar disorder is often characterized by circadian rhythm abnormalities, including decreased need for sleep and rapid cycling and switching. This appears to be particularly true among pediatric bipolar patients, where cycling and switching is more rapid than among adult bipolar patients [1-3]. Decreased sleep has even been noted as one of the earliest symptoms discriminating children with bipolar disorder from those with attention deficit hyperactivity disorder (ADHD) [4]. For these reasons, circadian rhythm abnormalities in general, and circadian clock genes in particular, have been proposed as possible mechanistic underpinnings for bipolar disorder, particularly for the phenomena of cycling and switching [5-14]. Associations between seasonal affective disorder (SAD), a variant of bipolar disorder, and polymorphisms in the clock genes *PER2*, *ARNTL/BMAL1*, and *NPAS2 *have been reported [15,16].

We previously described the identification of clock gene D-box binding protein (*DBP*) as a potential candidate gene for bipolar disorder [6] using a Bayesian-like approach called Convergent Functional Genomics. In additional work where we used an expanded Convergent Functional Genomics approach in a mouse pharmacogenomic model for bipolar disorder, we identified a series of other clock genes (*ARNTL/BMAL1*, *CRY2, CSNK1D*, and *CCR4/nocturnin*) as potential bipolar candidate genes [17]. Two subsequent reports have shown some suggestive association for one of these genes, *ARNTL/BMAL1*, in human bipolar samples [18,19]. *ARNTL/BMAL1 *is upstream of *DBP *in the circadian clock intracellular molecular machinery, driving the transcription of *DBP *[20,21].

To further assess the role of *DBP *in bipolar and related disorders, we have conducted and recently reported behavioral and gene expression studies in mice with a constitutive homozygous deletion of *DBP *(*DBP *KO mice) [14]. The studies in *DBP *KO mice revealed two other, closely related, clock genes whose expression levels were also altered: *RAR*-related orphan receptors alpha (*RORA*) and beta (*RORB*). Both *RORA *and *RORB *expression was increased in the amygdala and decreased in the pre-frontal cortex in *DBP *KO non-stressed, depressed-like mice. The *ROR *proteins, retinoid-related transcription factors, are in the steroid hormone receptor superfamily and play regulatory roles in neurogenesis, bone metabolism, and circadian rhythms (reviewed in [22]). *RORA *expression is widespread and appears to oscillate rhythmically in some tissues [23]. One of its roles involves activating transcription of the clock gene *ARNTL*/*BMAL1 *and regulating its circadian oscillation [24]. *Staggerer *mutant mice, which lack *RORA *activity, exhibit an enhanced response to novel environmental stress, mediated through corticosterone circadian rhythm abnormalities [25]. Of note, corticosterone abnormalities are prominent clinical findings in human affective disorder patients [26]. *RORB *expression is more limited than *RORA *and is highest in the eye, pineal gland, and brain, particularly in the primary sensory cortices (layer IV of the somatosensory cortex) and the suprachiasmatic nuclei of the hypothalamus (reviewed in [22,27]). Like *RORA*, *RORB *expression is known to change as a function of circadian rhythm in some tissues, and *RORB *-/- mice exhibit circadian rhythm abnormalities.

Here we report association studies for *RORA *and *RORB *in a pediatric bipolar disorder cohort. Bipolar disorder in children and adolescents is characterized by more rapid cycling and switching compared to adult bipolar disorder [1-3], possibly due to ongoing developmental processes and increased plasticity. Given our hypothesis that clock genes may underlie cycling and switching and the fact that *ROR *proteins play a role in development and neurogenesis, we reasoned that a pediatric bipolar cohort may represent an enriched pool in which to test for genetic association with illness. We therefore genotyped 312 *RORA *and 43 *RORB *single-nucleotide polymorphisms (SNPs) in two pediatric bipolar sub-cohorts: a family-based sample of 153 trios (each trio consisting of an affected proband and both parents) and a case-control sample of 152 cases (all independent from the family-based samples) and 140 independent controls.

## Methods

### Sample Identification

Subjects were ascertained from families recruited for genetic studies of pediatric psychopathology at the Clinical and Research Program in Pediatric Psychopharmacology and Adult ADHD at Massachusetts General Hospital [28-31]. All study procedures were reviewed and approved by the subcommittee for human subjects of our institution. All subjects' parents or guardians signed written informed consent forms and children older than 7 years of age signed written assent forms.

Potential bipolar disorder I (BP-I) probands were ascertained from our clinical service, referrals from local clinicians, or self-referral in response to internal hospital advertisements. Subjects' parents were administered a phone screen reviewing symptoms of DSM-IV BP-I and, if criteria were met, subjects were scheduled for a face-to-face structured diagnostic interview (described below). There were two sources of controls. The first group of controls was ascertained from outpatients referred for routine physical examinations to pediatric medical clinics at each setting identified from their computerized records as not having ADHD and who were found not to have BP-I on structured diagnostic interview. The second group of controls was selected from the Healthy Volunteer Specimen Bank (HVS) at the Harvard Medical School-Partners Healthcare Center for Genetics and Genomics. Healthy volunteers had also signed informed consent specifically allowing future DNA analyses. A thorough medical history and physical exam was performed to exclude all active diseases and current medication use and to obtain information such as lifetime tobacco use (yes or no). Controls were excluded if they had either ADHD or BPD. Other psychiatric disorders were not used as exclusion criteria.

### Diagnostic Procedures

All affected probands in the current analysis were diagnosed with bipolar I disorder according to DSM-IV criteria. The DSM-IV requires subjects to meet criterion A for a distinct period of extreme and persistently elevated, expansive, or irritable mood lasting at least one week, plus criterion B, manifested by three (four if the mood is irritable only) of seven symptoms during the period of mood disturbance. Also recorded was the onset of first episode, the number of episodes, offset of last episode, and total duration of illness. Psychiatric assessments of child family members (younger than 18 years) were made with the KSADSE (Epidemiologic Version) [32] and assessments of adult family members were made with the Structured Clinical Interview for DSM-IV [33]. Diagnoses were based on independent interviews with mothers and direct interviews with the children older than 12 years of age. Data were combined such that endorsement by either reporter resulted in a positive diagnosis. Interviews were conducted by extensively trained and supervised psychometricians with undergraduate degrees in psychology. This training involved several weeks of classroom instruction of interview mechanics, diagnostic criteria, and coding algorithms. They also observed interviews by experienced raters and clinicians and were observed while conducting interviews during the final training period. A committee of three psychiatrists, each board-certified in both child and adult psychiatry, resolved all diagnostic uncertainties. The committee members were blind to the subjects' ascertainment group, ascertainment site, and data collected from family members.

Probands were selected for analysis if the age-at-onset of bipolar disorder was 18 years or younger. The sample for family-based association analysis consisted of 153 affected probands (age (mean ± S.D.): 17.5 ± 11.6 years; BP-I onset: 7.7 ± 4.9 years) and both parents (153 trios); the sample for case-control association analysis consisted of 152 independent, non-overlapping cases (age: 20.3 ± 12.1 years; BP-I onset: 9.1 ± 5.0 years) and 140 controls (age: 42.9 ± 10.3 years). Thus the combined samples comprised 305 BP-I probands. Within our sample, 97.5% (N = 429) of individuals in trios, 98.0% (N = 147) of cases and 89.4% (N = 118) of controls were Caucasian.

### SNP Tagging and Genotyping

SNP genotype information for the CEPH population (Utah residents of northern and western European ancestry) was downloaded from the Phase II HapMap data (release #20) for regions surrounding each gene (750.7 kb for *RORA *and 208.1 kb for *RORB*). We used the Tagger program as implemented in Haploview http://www.broad.mit.edu/mpg/haploview/[34] to select pair-wise tag-SNPs with minor allele frequencies (MAF) = 0.05 and an *r*^2 ^threshold of 0.8. In total, 332 tag-SNPs from *RORA *(99% of alleles captured; mean *r*^2 ^= 0.959) and 44 tag-SNPs from *RORB *(98% of alleles captured; mean *r*^2 ^= 0.959) were chosen for genotyping. Primers were designed using MassARRAY's Assay Design software (Bruker-Sequenom, USA) and were purchased from Integrated DNA Technologies (USA). Genotyping of samples was performed as single-base extension reactions (iPLEX) using the MassARRAY mass spectrometry system as previously described [35]. A list of the genotyped SNPs and the assay primers used can be found in Additional file 1.

### Data Analysis

A number of quality control measures were implemented to ensure accuracy of the data collected. Genotypes from intra- and inter-plate controls were compared for identity, and negative test controls were confirmed to have no genotypes called. In addition, assays that failed in over 10% of the samples (14 SNPs) were excluded and samples that failed in over 10% of the assays (33 samples) were excluded. The genotyping rate in the remaining individuals was 99.38%. Families with greater than 5% Mendelian errors (4 families) and SNPs with greater than 10% Mendelian errors (1 SNP) were excluded, and genotypes causing remaining Mendelian errors were set to missing. SNPs out of Hardy-Weinberg Equilibrium (*P *< 0.001; 6 SNPs) were excluded from analysis. These measures resulted in a final set of 312 *RORA *SNPs (93% of alleles captured; mean *r*^2 ^= 0.959), and 43 *RORB *SNPs (98% of alleles captured; mean *r*^2 ^= 0.959).

Family-based transmission disequilibrium tests (TDT) and case-control association tests were conducted independently on the two sample sets using the program PLINK http://pngu.mgh.harvard.edu/~purcell/plink/[36]. Bonferroni correction for multiple testing was implemented based on the number of SNPs analyzed per gene. The critical *P*-value for a positive association was therefore 1.6 × 10^-4 ^for SNPs from *RORA *and 1.2 × 10^-3 ^for SNPs from *RORB*. In addition to these separate analyses for the case-control and the family-based samples, a combined odds ratio for the two sample sets was determined via the method described in Kazeem and Farrall [37]. Haplotype analyses were performed for both sample sets using the Confidence Intervals algorithm in Haploview [34,38]. Haplotype block associations were considered significant with a two-tailed permutation-based *P*-value < 0.05 after 1000 permutations.

Association with genotyped SNPs and age-at-onset (AAO) of bipolar disorder was analyzed by performing a quantitative trait analysis using the option *qfam *in PLINK. This option takes into account family structure information, so we were able to include data from the family-based sample and the case-control sample in the same analysis. The AAO phenotype was set to missing for all controls. Results were considered significant with a permutation-based *P*-value < 0.05 after 1000 permutations.

## Results

**Figure 1:**
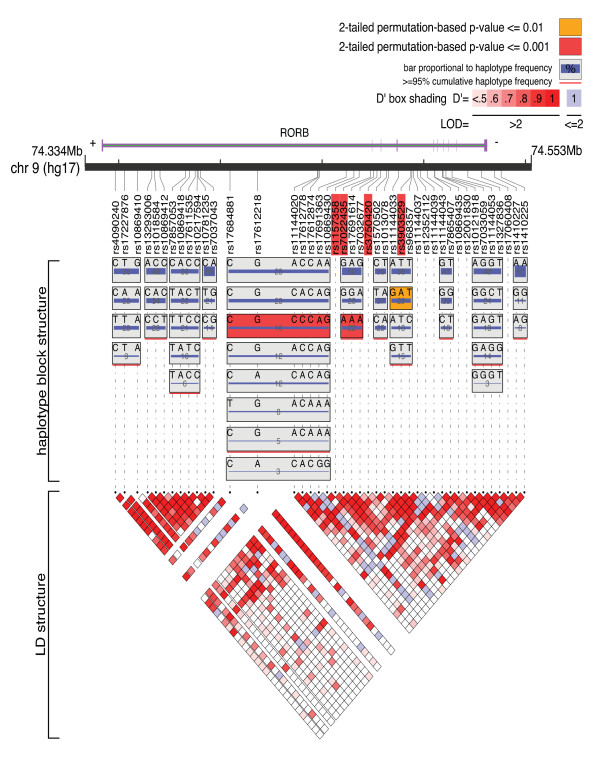
**Genomic Organization of *RORB *with Association Results**. The genomic location and organization of *RORB *are shown, with exons represented by vertical purple bars. All 43 analyzed *RORB *SNPs are shown and their positions are indicated. The four SNPs with significant associations to bipolar disorder in the case-control sample are highlighted in red. The haplotype block structure in the case-control sample as defined by the Confidence Intervals algorithm [38] in Haploview [34] is illustrated, with the linkage disequilibrium (LD) structure shown below. The two haplotype blocks with two-tailed permutation-based *P*-values < 0.001 are highlighted in red, and the block with *P *< 0.01 is highlighted in orange. Image created using LocusView http://www.broad.mit.edu/mpg/locusview/[51].

Several SNPs reached the nominal significance level of *P *< 0.05 in either the family-based sample or the case-control sample: 18 *RORA *SNPs and 8 *RORB *SNPs in the family-based sample, and 13 *RORA *SNPs and 16 *RORB *SNPs in the case-control sample [see Additional file 2]. However, after Bonferroni correction for multiple testing, no *RORA *SNPs and 4 *RORB *SNPs remained significant. These *RORB *SNPs were rs1157358 (*P *= 4.5 × 10^-5^) and rs7022435 (*P *= 1.1 × 10^-6^) in intron 1, rs3750420 (*P *= 7.9 × 10^-6^) in intron 2, and rs3903529 (*P *= 8.2 × 10^-5^) in intron 4 (Figure 1). All of these SNPs were significant only in the case-control sample and exhibited odds ratios in the opposite direction in the family-based sample (Table 1). No SNP exhibited a combined family-based/case-control *P*-value < 0.05. Of the four SNPs significant in the case-control sample, all but one exhibited Hardy-Weinberg Equilibrium (HWE) *P*-values >0.05 in both sample sets; the exception is rs3750420, for which the family-based HWE *P*-value was 0.037 (HWE *P *= 0.100 in case-control sample). The genotyping call rates for these four SNPs were between 97.66% and 100% in both cases and controls, and there were no significant differences in call rates between cases and controls (Fisher's exact test, all *P*-values > 0.05). Three *RORB *haplotype blocks in the case-control sample exhibited permuted *P*-values < 0.05 (blocks 5 and 6, *P *< 0.001; block 8, *P *= 0.002) (Figure 1 and Table 2). No *RORB *haplotype blocks in the family-based sample and no *RORA *haplotype blocks in the family-based or case-control samples remained significant after permutation.

**Table 1:** *RORB *SNPs with Significant Association to Bipolar Disorder.

SNP	Region	Minor Allele	Major Allele	Case-Control MAF	Case MAF	Control MAF	Case-Control OR	Case-Control *P*-value	TDT MAF (Founders)	T	U	TDT OR	TDT *P*-value	Combined OR	Combined *P*-value
rs1157358	Intron 1	T	C	0.137	0.218	0.091	2.802	4.5E-05	0.167	29	38	0.763	0.272	1.162	0.401
rs7022435	Intron 1	A	G	0.176	0.310	0.135	2.881	1.1E-06	0.203	34	51	0.667	0.065	1.150	0.373
rs3750420	Intron 2	T	C	0.237	0.375	0.201	2.388	7.9E-06	0.304	65	66	0.985	0.930	1.176	0.214
rs3903529	Intron 4	A	T	0.229	0.357	0.205	2.154	8.2E-05	0.247	34	55	0.618	0.026	1.094	0.537

MAF = Minor Allele Frequency

OR = Odds Ratio of the minor allele

T = Number of transmitted minor alleles

U = Number of untransmitted minor alleles

**Table 2:** *RORB *Haplotype Blocks with Significant Association to Bipolar Disorder.

Block	SNPs	Haplotype	Overall Frequency	Case Frequency	Control Frequency	*P*-value	Permuted *P*-value
Block 5	rs17684881	CGCCCAG	0.16	0.218	0.091	5.08E-05	< 0.001
	rs17612218						
	rs11144020						
	rs17612778						
	rs17612874						
	rs17691363						
	rs10869430						

Block 6	rs7022435	AAA	0.224	0.305	0.13	8.17E-07	< 0.001
	rs17691614						
	rs7032677						

Block 8	rs11144033	GAT	0.289	0.359	0.207	8.92E-05	0.002
	rs3903529						
	rs968357						

While our failure to replicate the association between *RORB *SNPs and bipolar disorder in the trios sample could be due to the fact that case-control designs exhibit higher power than family-based designs, it also raises the possibility that our results were due to population stratification within the case-control sample, particularly as there was a higher percentage of non-Caucasians among the controls (10.6%) than among the cases (2.0%). To investigate this possibility, we reran the SNP association analysis on the case-control data after removing all non-Caucasian individuals (and those with missing information) from the dataset, leaving a sample of 147 cases and 118 controls. All four *RORB *SNPs that were significant in the original sample remained significant after Bonferroni correction on the filtered Caucasian-only sample. An additional *RORB *SNP, rs7032677, was also significant after correction in this limited sample (*P *= 1.99 × 10^-4 ^in this sample, *P *= 0.0021 in the original sample).

For quantitative trait analysis with age-at-onset, 8 *RORA *and 2 *RORB *SNPs exhibited significant *P*-values after permutation [lowest *P*-values per gene: rs7175393 (*P *= 0.025) in *RORA *and rs12001830 (*P *= 0.037) in *RORB*; see Additional file 3]. The two significant SNPs from *RORB *from the AAO analysis, however, were not among the SNPs associated with the bipolar disorder "affected" phenotype used in the primary analysis reported above.

## Discussion

We identified a potential association between bipolar disorder and the retinoid-related receptor *RORB *in a pediatric bipolar disorder cohort. Four *RORB *SNPs and three haplotype blocks demonstrated positive associations in the case-control sample after Bonferroni correction or permutation. *RORB *was initially chosen for investigation due to its altered expression level in *DBP *knock-out mice (an animal model of bipolar disorder [14]) and due to the potential role of circadian clock genes in bipolar disorder.

The *RORB *gene encodes two isoforms, *RORB1 *and *RORB2*, which differ only in their N-terminal domains [39]. These alternative forms have different first exons and are believed to be produced as a result of transcription from alternative promoters. In *RORB1*, nine amino acids precede the first cysteine of the DNA binding domain, while in *RORB2 *there are twenty residues before this cysteine. The two forms of the protein exhibit differential expression patterns: *RORB2 *is found exclusively in the retina and pineal gland, and *RORB1 *is found mainly in the cerebral cortex (particularly in layer IV and, to a lesser extent, layer V), thalamus, and hypothalamus and is expressed only at very low levels in the retina and pineal gland [39,40]. *RORB2 *mRNA expression oscillates dramatically with circadian rhythms, peaking during the hours of darkness, while *RORB1 *expression fluctuates only mildly [39]. The DNA binding specificities and activities of the isoforms also differ. In rat, this results in *RORB2 *demonstrating increased activity in non-neuronal cells, though *RORB1 *and *RORB2 *perform equally well in neuroblastoma cells [39]. Taken together, these findings indicate that *RORB1 *is likely responsible for functions relating to the processing of sensory input while *RORB2 *is an integral member of the circadian clock machinery.

The four intronic SNPs of *RORB *we found to be associated with bipolar disorder are all downstream of the first exon in both *RORB1 *and *RORB2 *transcripts. If certain sequence variants of *RORB *(these SNPs or others in linkage disequilibrium with them) do indeed represent susceptibility variants for bipolar disorder, it is possible they do so by altering the expression of one or both of the *RORB *isoforms. One of the haplotype blocks exhibiting a positive association (block 5 in Figure 1 and Table 2) spans the region containing the *RORB2 *exon 1, from approximately 55.1 kb upstream to approximately 5.7 kb downstream of *RORB2 *exon 1. It is possible, therefore, that the positive signal in this haplotype is due to sequence variation in the *RORB2 *promoter that affects the location, timing, or magnitude of *RORB2 *expression, leading to increased risk for bipolar disorder.

Our findings should be considered in the context of important methodological limitations. Although our patient sample was relatively small, its focus on pediatric patients made it a potentially enriched pool in which to search for genes involved in rapid cycling and switching, such as clock genes. The significant association of four *RORB *SNPs and three *RORB *haplotype blocks with bipolar disorder in the case-control sample indicates that this may indeed be the case. However, the TDT odds ratios for the associated SNPs were not consistent with the highly significant case-control results. This raises the possibility that the case-control results could be false positives due to population stratification or that we had insufficient power in our replication sample.

Though we still observed significant association after limiting the sample to Caucasians only, we cannot rule out other sources of stratification, such as distinct ancestry. Alternatively, these findings could be the result of associated alleles of each marker on different haplotypes with the actual risk-conferring variant(s) in the two samples.

Despite these limitations, our results indicate that circadian clock genes in general and *RORB *in particular may be important candidates for genes involved in bipolar disorder. Of note, both *RORB *and *RORA *have nominally suggestive signals in three recently reported genome-wide association studies for bipolar disorder [41-43] that do not survive correction for multiple comparisons. This would be expected if our pediatric cohort does indeed represent an enriched sample in which to test for association with circadian clock genes that may be involved in cycling and switching. The nominally associated SNPs in RORB from these studies, however, do not overlap with those SNPs found to be associated in our study [see Additional file 4]. It is therefore necessary to verify these association results in other independent samples and to continue to study the relationship between *RORB*, other clock genes, and bipolar disorder.

Finally, it is important to note that *RORB*, *RORA*, and *DBP *were identified by us recently as possible genes involved in schizophrenia using a pharmacogenomic mouse model and CFG approach [44]. Overall, our findings are thus consistent with a model of heterogeneity, overlap, and interdependence of major psychiatric disorders [14,45,46]. Particularly intriguing from a translational standpoint is the possibility that the localized expression of *RORB *in layer IV somatosensory cortex [47] may contribute to integration of external and internal stimuli that have a bearing on response to stress, mood reactivity, and cognitive constructs in bipolar disorder pathophysiology [48-50].

## Conclusion

Our findings suggest that clock genes in general and *RORB *in particular may be important candidates for further investigation in the search for the molecular basis of bipolar disorder. These results are supported by our current understanding of the expression, localization, and possible roles of *RORB *in the brain and are also consistent with data from animal models of bipolar disorder.

## Competing interests

The authors declare that they have no competing interests related to this work.

## Authors' contributions

CLM conducted the genotyping experiments; PS supervised the genotyping experiments; CLM, SJG, and SVF contributed to data analysis; AED, EM, and JB contributed to sample collection and characterization; ABN, HLN, RK, and MTT contributed to the overall design of the project and candidate gene selection. CLM, PS, SJG, SVF, EM, ABN, HLN, RK, and MTT contributed to the writing of the manuscript. All authors read and approved the final manuscript.

## Pre-publication history

The pre-publication history for this paper can be accessed here:

http://www.biomedcentral.com/1471-244X/9/70/prepub

## Supplementary Material

Additional file 1 **Genotyped SNPs and assay primer sequences**. This table lists the SNPs genotyped and includes sequences of the assay primers used for genotyping. Click here for file

Additional file 2 **Association results for all analyzed *RORA *and *RORB *SNPs**. This table details the association results for all *RORA *and *RORB *SNPs analyzed and includes results from both case-control and family-based samples. Click here for file

Additional file 3 **Association results for age-at-onset quantitative trait analysis**. This table contains the results of the quantitative trait association analyses of *RORA *and *RORB *SNPs with age-at-onset of bipolar disorder. Click here for file

Additional file 4 ***RORB *SNPs associated with bipolar disorder in genome-wide association studies**. This table contains the *P*-values of RORB SNPs associated with bipolar disorder in our study and four genome-wide association analyses. Click here for file
