# Supplementary material for: Evidence for genetic association of RORB with bipolar disorder
Source: BMC Psychiatry. 2009 Nov 12;9:70. doi: 10.1186/1471-244X-9-70 (PMC2780413; doi:10.1186/1471-244X-9-70)
Supplement: Additional file 1 — Genotyped SNPs and assay primer sequences. This table lists the SNPs genotyped and includes sequences of the assay primers used for genotyping. [file 1471-244X-9-70-S1.doc]

Genotyped SNPs and assay primer sequences

| **Gene** | **SNP** | **Forward PCR primer** | **Reverse PCR primer** | **Extend primer** |
| --- | --- | --- | --- | --- |
| RORA | rs7181662 | ACGTTGGATGACTGGTCCTGTTAGGAACTG | ACGTTGGATGCATCTGACAGAATCTGGAGC | GTGAGCCGAGGGTGAACCAGCATT |
| RORA | rs1866007 | ACGTTGGATGGCCTGCTCTTTCTAAAGAAC | ACGTTGGATGAAAATTAAAGTTGGGTGGGG | CAACTTTTTTTCCTCATATGCTTCTT |
| RORA | rs782938 | ACGTTGGATGTTTGATGCCGATTCAGCACC | ACGTTGGATGGCTTCCTGATGAGAAGCAAC | CACCCTCTCTGCCCC |
| RORA | rs11630062 | ACGTTGGATGTCTTCCTTCTCTGTGGTTGC | ACGTTGGATGGGGTGGAAAACACAAATTCG | CCTCAGCCATCTTCC |
| RORA | rs2899662 | ACGTTGGATGTCCCTCCTTTACATCAACGG | ACGTTGGATGAGACCAGTGAGAACCAAAGC | ACATCAACGGCTTCCA |
| RORA | rs4775374 | ACGTTGGATGGATGCAGTTTGCTCATCCTC | ACGTTGGATGAGCCTGAGATCTGGCAACTG | CAGCCCGGCAGCACAC |
| RORA | rs16943318 | ACGTTGGATGAGTTAAAGGCAAGGGCTGAG | ACGTTGGATGTCAGGAAATAAGGGCCGATG | TAATCCCTTTCCCTCAC |
| RORA | rs4774371 | ACGTTGGATGACTTCTAATGCAAGCCAGAG | ACGTTGGATGCAACAGAGACTCCGTCTCAA | CCAGAGTTGAATGCCAC |
| RORA | rs113168 | ACGTTGGATGCTGTTATACAGGCTGTCCAC | ACGTTGGATGAGAGCAAAAGAATCTGAGGG | TGCTCTCCCTGTATCCCT |
| RORA | rs8039843 | ACGTTGGATGGAATCCATGGTAGCCACATC | ACGTTGGATGGTTGAGTACTTGGAAAGCCC | CCCTCGTGGTATCCCTCA |
| RORA | rs8025324 | ACGTTGGATGGAAGGAGGCAGAACAAAGTG | ACGTTGGATGAGCTGAATGTCCTTTCCCTC | TTGTAGAGAGAGATGCCC |
| RORA | rs8026340 | ACGTTGGATGCTATGCACAGTCATCATCCG | ACGTTGGATGTTAAAACCTTTTCAGGCGGG | AGTCATCATCCGAAGACCT |
| RORA | rs11631432 | ACGTTGGATGGTGGTTGCAACGAAACAAAAC | ACGTTGGATGCCCACTGTTCTGTATCTTCC | GACAAAACTTTGGGTGTTC |
| RORA | rs341380 | ACGTTGGATGGGGACTTACCTCAAGTGATG | ACGTTGGATGACAGATACCTCTGATGCCAC | GAGTGATGAGAGCATTGTT |
| RORA | rs2899666 | ACGTTGGATGAAGGCATGAAGAGTGGGAAC | ACGTTGGATGGCAACTGCAGTTGTGTTCTC | CCCTAACAGACCCGGCGTAT |
| RORA | rs11638433 | ACGTTGGATGATACATTCCAGACCACCTTG | ACGTTGGATGAAAGAGAGTGGCCTCAAGTG | GGTCGGTAGGCGTTCTCAAT |
| RORA | rs782947 | ACGTTGGATGGCAGCTCTACCTAGTCCTTC | ACGTTGGATGCCTTATAGGTTTCACACCAG | CCAGTGCAACCAGCCGTTCCA |
| RORA | rs12438866 | ACGTTGGATGGAAGACCTTGCCAGATTACC | ACGTTGGATGCACTCTTCCCATTCTTGAGC | CCCGAGCCATCTGACAAACTA |
| RORA | rs782913 | ACGTTGGATGAGCTTTTGCCTACTTCTCCC | ACGTTGGATGAGTGGAGCTGAGTAGAGAG | CCCACTACTTCTCCCCTCATGT |
| RORA | rs4613007 | ACGTTGGATGTTCTAGATCTGGCTCAACCC | ACGTTGGATGCCTTTTCCTACCTTGGTTTC | CCCACCCAGCTGCCTCTTACTA |
| RORA | rs11071587 | ACGTTGGATGCAGGGCAGAAGATTACATAAC | ACGTTGGATGCAGTTAAGCTTGTAATCGATG | ATTGGCTTTAAATCATTCCAGT |
| RORA | rs7166062 | ACGTTGGATGGCACAGTCTACAGATATATGC | ACGTTGGATGCCCCAACAACCACTGATCTT | GTTTGCATCTCTGAGAAATAAC |
| RORA | rs12591914 | ACGTTGGATGCCAGACCTACATCTCCATTC | ACGTTGGATGGGGCTCATGAAATGGTAGTC | CCCGTCTCAAGCCTATTCAAAAC |
| RORA | rs12903172 | ACGTTGGATGTGAACTACACACAAGTGGGC | ACGTTGGATGCGGTTTTAGTGTTTCCCTTG | GTGGCTGTGACTGTAATTCAGCA |
| RORA | rs341365 | ACGTTGGATGAAGTTGAAATGCAGCTAGAG | ACGTTGGATGTTGCTTTTTGCTCCAGAATG | AAGGAATGCAGCTAGAGATATCC |
| RORA | rs340009 | ACGTTGGATGGGCACTTATAGAAATACGCTG | ACGTTGGATGAGGTATGTATACCATGTCAC | TCCGATGTACCTCATTTTCTGATA |
| RORA | rs17191582 | ACGTTGGATGAGGACAGATCATCCAGAGAG | ACGTTGGATGTGTTGCCACTGACCCTTGAG | GAGAGCTTTGCAGAGATCTGGTAA |
| RORA | rs8043356 | ACGTTGGATGCATTTGCTAAGCCTTAAGGG | ACGTTGGATGCTGTAGCCCTTGACCTTATC | TTGAATTAACATCTTTACCTCAAGC |
| RORA | rs17270216 | ACGTTGGATGAACTCTGTCCTAGCACGTTC | ACGTTGGATGAGATGAGACAAGGCAACAC | AAGTGTTTTTACATCAACAGCGTAT |
| RORA | rs17270501 | ACGTTGGATGCCCACACTGTAGCTAAATCC | ACGTTGGATGAGAGAATCTATGAGGAGGAC | GATGGTAGCTAAATCCAGACTTTAC |
| RORA | rs4774381 | ACGTTGGATGTATAAGGTCAAGCCCAGCAG | ACGTTGGATGCGGCAATTGTGTCACATGCT | GCACCCAATCCAAACTTATCTTCCTG |
| RORA | rs1866006 | ACGTTGGATGCTGTTTAACTGAAGTGAGGG | ACGTTGGATGCTAGCAACTCTGAGACTCTG | CGGAGGGTTGATTTATAAATTACTTC |
| RORA | rs4775371 | ACGTTGGATGCTAAAAAAGAGCAAAGAACC | ACGTTGGATGTCAGGATGTAGCTAGCTTTC | AAAACAAAGAGCAAAGAACCTAATGT |
| RORA | rs11632600 | ACGTTGGATGTTGCTTGATGTTACGGATGC | ACGTTGGATGTGCATTCACAGCCACGTTTG | CTTAAGGATGCAACTTAAGAGGAGGA |
| RORA | rs17204489 | ACGTTGGATGGGAGCTTCCAGTCTAAACAG | ACGTTGGATGTAAACTGTGTTGGAAAGGGC | CCCGGTCAAATTCTTAGCTCAAAAATA |
| RORA | rs12916690 | ACGTTGGATGACTCAAGTTAACCTCTAAGC | ACGTTGGATGGGCAATTCAGGATAACTCTC | CACCAAAGTTAACCTCTAAGCTGTTGT |
| RORA | rs4774388 | ACGTTGGATGTCTGTTCCGTAGGGAAAGAG | ACGTTGGATGATGGAGGCTTTAGTCTCTGG | TATGGCCAGCTAATGTTTATTGCATACT |
| RORA | rs1869488 | ACGTTGGATGGGACACGTCATATAAATCCC | ACGTTGGATGGCTCAAAAGTTTTTTGTCTAC | GGACACTAGATAAAATGAAACTCTACAT |
| RORA | rs12324086 | ACGTTGGATGGGATTCCACATTGCATTGAG | ACGTTGGATGAACAACAAGGAAAACCAAGG | CCCCTGTGTTGTCTACTTAGTCTCCCCCA |
| RORA | rs10519070 | ACGTTGGATGGACTTCACAACCAGGCTTTC | ACGTTGGATGCTGTGCCTATCTCAGTTTGG | TGCTCTTCTGCTCCT |
| RORA | rs17204952 | ACGTTGGATGAAGAGATCCGGGTTAGCCTG | ACGTTGGATGAGTTCTGCAAAACTGGAGGG | AGCCTGTGCTGTTGT |
| RORA | rs1437543 | ACGTTGGATGCTAAATGAGTTAGAGGCCAC | ACGTTGGATGGTCAGACTATTTGGTGTTCC | GACAGTAGGGCGGTT |
| RORA | rs4775301 | ACGTTGGATGAGGCCACTTAAGCAAACACC | ACGTTGGATGGCTGGGCAAATCTGCATCTG | AGCATCACAAAGCCCA |
| RORA | rs341387 | ACGTTGGATGGCAGTGGGAAGAAAGACAAG | ACGTTGGATGCATGATCTTTGGACCCTTGC | AGAGCAGAGGCTGACA |
| RORA | rs893287 | ACGTTGGATGTAGCTTGTGCCAGAACTAGG | ACGTTGGATGAACCATCTCCCAAGGCACAC | GGGGTTTGTTAGGGTG |
| RORA | rs11071591 | ACGTTGGATGGGATGTGTCTGTGCACTATC | ACGTTGGATGGCAGAGCCATTTGGATCAAC | CACACTATCCAGCCCCA |
| RORA | rs1370429 | ACGTTGGATGTGCTTAAAGGGCTGACCATC | ACGTTGGATGATCCTGGTCTCCTAAATGGG | GCTGACCATCTGATGTA |
| RORA | rs16943489 | ACGTTGGATGTTGCATACCCTGTTTGGGTC | ACGTTGGATGTCATTACTTTGGACATGGGC | TTGAACACTAGGCTGAC |
| RORA | rs4238351 | ACGTTGGATGCTTCCCCAACTCCTAAACTG | ACGTTGGATGGGACTGCTTGCTTATTCCAC | ATTCCTGCCCAGCTATCA |
| RORA | rs17191463 | ACGTTGGATGTAACTCTATGGTTGAAAGG | ACGTTGGATGAACGCAGTTGCCATGTGTTA | CTGGTTGAAAGGTTCTGA |
| RORA | rs12902540 | ACGTTGGATGCCTAGGAGTGAAATTGCTGG | ACGTTGGATGGGTTGGAATATAACGTTGTC | GTGTTGAGGGGCTACCAA |
| RORA | rs782928 | ACGTTGGATGGCTCTCTTTCATATCTCCAG | ACGTTGGATGTGAAGCACCTCCCAGTAAAG | TTCATATCTCCAGAGTCCA |
| RORA | rs4775362 | ACGTTGGATGTGGCTTTATCAGGCACACAG | ACGTTGGATGAACCCAAAGCAGTAGGTAGG | CAGTCACACAGCAAGGTCC |
| RORA | rs11638592 | ACGTTGGATGTGATCTTTGCAGGGCAGAAG | ACGTTGGATGAGCAGTGAAACAATGAGGGC | GGATGGCAGAAGCCTGTGG |
| RORA | rs4775369 | ACGTTGGATGTATTTAACCTCTCTGGGCTC | ACGTTGGATGTATCTGACCCGCATAATGGC | CAGTGCTCTCATGTAGAAAA |
| RORA | rs716593 | ACGTTGGATGAAGATCCTGGTGCTGCTTTG | ACGTTGGATGAGAGGCTGGTCAATAAGGAG | CCCCTTCTCTGGCCATGCCGA |
| RORA | rs782945 | ACGTTGGATGCCAGCAGGTTTCTATTAGCC | ACGTTGGATGTGATTGCGACACTCAGTTCC | GGTTTCTATTAGCCAATAAGC |
| RORA | rs17204367 | ACGTTGGATGGTTTACATAGAAATGGATGA | ACGTTGGATGTGTCTCCTTATGTGTGTGTG | TGGATGAAAAAAGTAGTCCTA |
| RORA | rs340021 | ACGTTGGATGTCCAATTATGGCATAGACTC | ACGTTGGATGCAGTAGCCTTTCTTTCAGAC | GTTTCACATAATTTCCCCATAT |
| RORA | rs10851685 | ACGTTGGATGATCCGAGTGTACAAGGCAAC | ACGTTGGATGGAGCCCAAACTTGAGGATAG | GGGCAAGGCAACTTCAATCTAT |
| RORA | rs2279291 | ACGTTGGATGACTTAGCTGTCAGTCTGGAG | ACGTTGGATGTAGCACTTTCTACTCCACTG | CCCCGCATTCTCTTGAAAATGTT |
| RORA | rs17204848 | ACGTTGGATGCTACAGAGGGTTATCAAGGC | ACGTTGGATGCAACATGTGAGTGCCTTGAG | AAGGTTTATCAAGGCCTGAATGT |
| RORA | rs4638514 | ACGTTGGATGCTCAAAGTTTTCCCTCATAG | ACGTTGGATGGAATACCATTCTTGCTTGTC | GGGCGGGGGTCGTAAATGCAATT |
| RORA | rs4378570 | ACGTTGGATGCCTTCACCAAAGCTTCTTCC | ACGTTGGATGACTCACCTTCTGAAAGACCC | CCCCATGGCCCACTGACCCCCGGT |
| RORA | rs4774386 | ACGTTGGATGTCAGCCCCTCTACCTGTTTG | ACGTTGGATGGATTTTAGTTCCTCTGATGC | TTACCTGTTTGAAAATCTTTGCTA |
| RORA | rs951265 | ACGTTGGATGTAGAGATGGGTAGATGGGAC | ACGTTGGATGTATCTTCTTAGAAAGCACCC | AATTTTTTTTAAGCCATAACTATCA |
| RORA | rs11633266 | ACGTTGGATGTTTATGAAGTGCCCGGGCTG | ACGTTGGATGACTCCGAGACAAGAGAGAAG | GTCCTGGCTGTGTTTGTGTTACTGA |
| RORA | rs726914 | ACGTTGGATGGAACACATTTGGCATGTTTCC | ACGTTGGATGATCCTCCCAAAGCTAGATGC | GGGAGTTTGGCATGTTTCCATTTAT |
| RORA | rs2289163 | ACGTTGGATGAAGAAGTCGATAAGAAATC | ACGTTGGATGAAAAGAGAGGAAACCTCCCG | GGGCTCTTAATAATTTTTCCTCCCAA |
| RORA | rs1820357 | ACGTTGGATGGAATGAGGGGATTAAAAATG | ACGTTGGATGCTGCAGATCCTTTTAGTGTG | GGGAGGGGATTAAAAATGAAGAAAA |
| RORA | rs12324440 | ACGTTGGATGTACCCACAACTTCCTGGTTC | ACGTTGGATGGGAAAAGAGCAGGTTGAGTG | GGTGATCATGGATCCCCGGGCTGGTG |
| RORA | rs1403739 | ACGTTGGATGCTTCTGATGCATGAGGCTCC | ACGTTGGATGCTGTTGGGCTGGTAATCTAC | GGGAGGCATGAGGCTCCGAATTTTAT |
| RORA | rs13329643 | ACGTTGGATGAACATTTTCCAGTGGTTCCC | ACGTTGGATGTGGGTGAGATTCCATTACAG | CCTAGATTTCATAAAGTCTGAAGTCTT |
| RORA | rs7168905 | ACGTTGGATGATGCTCACTTAAACAGGTAG | ACGTTGGATGTTTACTCTGTGTGTAGTGGG | GGGAACTTAAACAGGTAGATATGTAAG |
| RORA | rs339996 | ACGTTGGATGCTAAGATTCAAACCCGGGTC | ACGTTGGATGTTGTTAACGGAAGGGAGTGG | GAAGCTAATGTCCGAGTCCCTTCCAATA |
| RORA | rs1437547 | ACGTTGGATGGACAGTCCAATCTTGACAAT | ACGTTGGATGTGTCTTTGTCAGTGCTTCCC | TGGACAATTATTAACTATAACTCATCAA |
| RORA | rs708683 | ACGTTGGATGCTCTCAGAGCCTGAGGAAAG | ACGTTGGATGGAGGGCATGAAGCTCAAATG | GGCCGAGCCTGAGGAAAGCTCGTCAATG |
| RORA | rs12592999 | ACGTTGGATGAATAGCTCGGCATCTTGTGG | ACGTTGGATGCTTTGCCATGAATGACAGAG | GGCGGAGCAAAGTATTTCCTTAGGCTGA |
| RORA | rs4775314 | ACGTTGGATGTGATGGAGACAAGGCAGTTC | ACGTTGGATGCCCAATCAGTCAAACGACAC | CAGTTCTGCCGACTT |
| RORA | rs8034950 | ACGTTGGATGGGGAAAAATTGCTGCCTCAC | ACGTTGGATGGCTTTGCTCCTCAGAACTAC | ACCCATTTCTCCCCTT |
| RORA | rs6494217 | ACGTTGGATGACAGGCTCTGATAACCTCAC | ACGTTGGATGCAAACATGCTCACCAGTGAC | ACTCTTCCCTCCAGAC |
| RORA | rs1425287 | ACGTTGGATGCCAGAGAAAATGTTAGGGTG | ACGTTGGATGGCCGGTTTTACTACTAGAGC | TTAGGGTGGCATCAAC |
| RORA | rs12899546 | ACGTTGGATGATACACTGGTCTTATTTCC | ACGTTGGATGGCCCCACTTAGTTTTCTTTG | GTCTTATTTCCCCGTGT |
| RORA | rs6494219 | ACGTTGGATGAGGTCATGGAATGAGTCAGG | ACGTTGGATGTTTGTGGATCCACCGATCTC | AAGAGAGAAACAGAGGC |
| RORA | rs1465812 | ACGTTGGATGACACATCGCTCACTTTCCTC | ACGTTGGATGAGGCCTCTCTGCTAAAGATG | AGCTTTCCTCCACTGCTC |
| RORA | rs11630018 | ACGTTGGATGACCTAAACTGGCAGTCCAAC | ACGTTGGATGTTCTCTGAAGTGCTCTGCTC | GGCAGTCCAACAGTACTT |
| RORA | rs17270745 | ACGTTGGATGCATTTCAGTCAGAGGTGAGG | ACGTTGGATGGCTTTCTAAGCTTACTCCTG | TGGATTTTCATTGGCTTCC |
| RORA | rs4774392 | ACGTTGGATGTACCTGGAATTCCAGGTAG | ACGTTGGATGGCATATTCCATAGGTTTAGG | CGTAGGCCCATATGAGAAG |
| RORA | rs17303572 | ACGTTGGATGCACTCCAGATAAGATCCACC | ACGTTGGATGAAGGCCCATATGACTGACTG | ATCCCCCTCTTGAAGCTCCC |
| RORA | rs1351546 | ACGTTGGATGTTGGTGTGGCCTGAGGTTTC | ACGTTGGATGAGCCATAGATGTTTTCACTG | ATGTCTCTCCTGATTATCAC |
| RORA | rs1589702 | ACGTTGGATGTTAGAAAGTGTTCCCTCTGC | ACGTTGGATGCTCCATCAATGGATTCTACC | GAAGGATTGGTGCTAACTTT |
| RORA | rs1816624 | ACGTTGGATGAGCATTATTTGTGGATGCTG | ACGTTGGATGATCCCTACACAGCCATTCAG | GACATCAGAACTCCTTCCATC |
| RORA | rs2433026 | ACGTTGGATGCACCTAAGGGCATAACCTTG | ACGTTGGATGGTGGCTCATAGCTGAATTGC | TGGCCAGGCAGCTCCACTTTA |
| RORA | rs4775360 | ACGTTGGATGGTCTATTCCACTAACCCGAC | ACGTTGGATGTCCACGAAATTTGTTGGCTC | GAGAACAGTGGTGTGAAACAA |
| RORA | rs7172348 | ACGTTGGATGGAAGAAACCAGTCAACAGGC | ACGTTGGATGCCAGAGATGTTTTGTGTCCC | ACATTCAACAGGCTGCATAATA |
| RORA | rs7181803 | ACGTTGGATGCCAGTTTGCCAGGAATTTTC | ACGTTGGATGTATCCCAGTTTTGCCCAGAC | GTGCCAGGAATTTTCCTGGTTT |
| RORA | rs12595623 | ACGTTGGATGACAAGGGTGGGTTTGGTTTC | ACGTTGGATGAACGGTTTGTGGCCAATGAG | CTCGGTTTCCTTCACTTATACAA |
| RORA | rs8034880 | ACGTTGGATGTTCCTATGACAGCTCCCTTC | ACGTTGGATGTTAGTCATCCCTGAGTCCTC | GGAAAAACAGGAAATAACCTAAG |
| RORA | rs12440095 | ACGTTGGATGCCTCTCCCCCTAAAACTTTC | ACGTTGGATGATGCTCTGTCTGACTGGTTC | CCCACCCTAAAACTTTCAGAAGAA |
| RORA | rs10519107 | ACGTTGGATGGACATTTTGTAATCCTGTG | ACGTTGGATGCTTGAACTTGACCAACATGC | ATTTGAATCCTGTGTAACTCTGAT |
| RORA | rs1403737 | ACGTTGGATGAACCCCAATTTGGCATGTCC | ACGTTGGATGTCAACTCCCAACACTCGTTC | GAGTGGGACCCATTAAGAGTCTAT |
| RORA | rs7175883 | ACGTTGGATGTGGTCGAGAGAAGAGTTAGC | ACGTTGGATGGTCTGCTGAGCTGCATTTAC | GTGAAAGAAGAGTTAGCAGGCATT |
| RORA | rs12443239 | ACGTTGGATGCAAACGGAATGTCTGATCAC | ACGTTGGATGCGAAGTCCTTTTCCCTTAGC | ACACAGCCATATTTTTCCCCCTTAA |
| RORA | rs8040332 | ACGTTGGATGCTGCTCTTCATATCCACAAC | ACGTTGGATGAGAACATGCTAAGGGTTGGG | TTGCAACTGAAAAAATTCAGGCTAC |
| RORA | rs8036966 | ACGTTGGATGGGTCAGAATGATCCACACTC | ACGTTGGATGGTTTACCCCTATCCCTAGTC | TCCAAACTCTCATCTAGTTGCTTTTA |
| RORA | rs4332688 | ACGTTGGATGCTGGTGATGCAAATACAAGG | ACGTTGGATGATGTTGGCATTTTGGAGTGG | GGCCCATACAAGGCATCATCCATTTA |
| RORA | rs12324535 | ACGTTGGATGAACTGCTGCGATGAAGAGTG | ACGTTGGATGGTAAAGCTTAATAGCATGGG | GAGTAATGAAAAGCAGCCCTCAAAAA |
| RORA | rs7178442 | ACGTTGGATGTGGTAGGATTTGAGTTGAGC | ACGTTGGATGGGAGGACGGTCTGTCTAATC | TTTTATTTGAAGAATAAGCAGACTTG |
| RORA | rs4594196 | ACGTTGGATGCAGACAGCACTACTGTCAAC | ACGTTGGATGTAAACCTCTTTGCTGGGATG | CACGTATATTGATTAAATTAGCCCACA |
| RORA | rs7164773 | ACGTTGGATGAGGTTTGATGTCCCATAAGC | ACGTTGGATGGAAACAACGGTCTCTTTTCC | ATAAGTGTTTATTAAATTAAGCCCTCC |
| RORA | rs2306502 | ACGTTGGATGGATTGTGCAGCCGAAAGAAG | ACGTTGGATGAGCACAGAACATTCCTGTGG | GGCAGAAAGAAGGCCCGGTGCCCTTGG |
| RORA | rs1110418 | ACGTTGGATGTATGTCCACCACAGAGTATG | ACGTTGGATGTTTCCCCTTGAACTTGACTC | AAGCAGAGTATGAATATGAAAAAAGAA |
| RORA | rs930358 | ACGTTGGATGTGTCCATAGAGATGAGATTG | ACGTTGGATGGGCTTATGAAGCAAATTCGC | GGCTGTAGTTTAGAATACCTTTAACTTT |
| RORA | rs341403 | ACGTTGGATGGTCTGATTCTGCCTTGACTC | ACGTTGGATGATGTTAAGCAGAGACGCTCC | CCCTCTAATTTTTAGGAAGTTAGAGTGC |
| RORA | rs754499 | ACGTTGGATGGGTTTGCCAGTTAAAGAGGG | ACGTTGGATGGCCCACACTTTCACATCTTC | GGGAAATATTCCTGGTGGAGAGAATAAC |
| RORA | rs2062091 | ACGTTGGATGTTCAGACTGCAAGGACACTG | ACGTTGGATGTCAGCAGAGTACCTGGAATG | CTCCCAAGCTTCGAC |
| RORA | rs341390 | ACGTTGGATGGCACTTGTATAGTCAGCGAG | ACGTTGGATGCTGTAAAGGCCCTTCATCTG | GTCAGCGAGGAGACT |
| RORA | rs782926 | ACGTTGGATGAAAGAGGGAGAGTAGGAGAG | ACGTTGGATGTTTGCTCAAATGCCACTCCC | GGGAAAGGGAAGCGG |
| RORA | rs974828 | ACGTTGGATGTCCTCTTCTTGCACTTTGGC | ACGTTGGATGCCTTCTAAAGCAAAAGTCTG | CACTTTGGCCTAAGCA |
| RORA | rs1673336 | ACGTTGGATGGAACTGGAAGAACACTGGAC | ACGTTGGATGGTCCATCAAATAGGGACAAC | TGGACTTGGAGTCAGA |
| RORA | rs930359 | ACGTTGGATGCCGCTTTTGGATTTTGCTTC | ACGTTGGATGATGGACAAGTCTTTCTGAGG | GACACCACCATCTGGTA |
| RORA | rs7176717 | ACGTTGGATGAAATGGCACAAAGAAGGCCC | ACGTTGGATGGGCCTAAATCCTGGCAACTA | TGAGGCCCACGGGTTTG |
| RORA | rs8038077 | ACGTTGGATGTAGCCAGGACACAAACTCTC | ACGTTGGATGATGGCAACTGACACATGACC | CCCCACAGCACTCTCTAT |
| RORA | rs10519097 | ACGTTGGATGGTTTATGGAGAGAGTGAGCC | ACGTTGGATGGATTCAGTTGTGCTCTTGGG | AATGCACAGACACTATCA |
| RORA | rs235512 | ACGTTGGATGAGATGTTGCTTGCCTCTCTG | ACGTTGGATGCCTCCTGGGCATTACTAAAG | CGGGACCCCCAGGAATCT |
| RORA | rs2414686 | ACGTTGGATGTATGACACTACAAGGACAGC | ACGTTGGATGCAGTTCTAACTCGATTTTC | GTGGCTGAAGAATGGAAT |
| RORA | rs8024716 | ACGTTGGATGGGAATCCATTTAGTTCCCCC | ACGTTGGATGGAAGCAGACGCTTTCACAAG | TTCTTGACTGCCCCCCACC |
| RORA | rs341413 | ACGTTGGATGTGGAAGAGCACCACCTATTG | ACGTTGGATGAGTTCCCAGGTACTGCCCAT | GGAAGGTTAACCTCTAGAC |
| RORA | rs10519105 | ACGTTGGATGTATTCCCTAGCAACTCATCC | ACGTTGGATGAATTCGGAGCCTCATGCATC | CTCGCAACTCATCCATCGTT |
| RORA | rs17204440 | ACGTTGGATGGGTATAGCTATCCTCAAGTC | ACGTTGGATGAAATTTCCCAAGGGCAGAGG | AAATAAATGTGAAGCACACC |
| RORA | rs4265751 | ACGTTGGATGGGAGCCTATAAAGTCCACAG | ACGTTGGATGCAAACCCCAATATCATCTC | GGGCCACAGTGGGTCAAAAA |
| RORA | rs10519076 | ACGTTGGATGGTCCTACTGATGAAAGATAG | ACGTTGGATGCACACAGCTAAATGCTGCAC | CCCCATGCAAAAGCAAGAATC |
| RORA | rs7168008 | ACGTTGGATGATGGGCTTAGGGAATACAGG | ACGTTGGATGCGTGATTCATTGAGATGCGA | GGGAAATAGACTTGTGCAGAG |
| RORA | rs4775297 | ACGTTGGATGGCTAAAGGACACCAACCTTC | ACGTTGGATGGCTCAGTAGCCCTATTTCAG | GGAGGGGTTGACAGTGGATCA |
| RORA | rs4775310 | ACGTTGGATGAAAGCAAGGCATACCCTAAG | ACGTTGGATGTAATACGTGTCTGACCTGAG | GCATCGGCGGATTTTTTTATTT |
| RORA | rs4774390 | ACGTTGGATGCCCAAGCCTGTCAGTATTTC | ACGTTGGATGAGCACAAAGGCACTTCAAGG | CCTCCTGTCAGTATTTCTAAGCA |
| RORA | rs7172917 | ACGTTGGATGGCAAGGCGGGAAATGAAATC | ACGTTGGATGCTTACCTCCCTGTTTAAGGC | GGATCACAGACACCAGGGCTCGT |
| RORA | rs726955 | ACGTTGGATGTCATAGTGACCCTGCAGAAC | ACGTTGGATGCGGATGATAAGTTTGCTGTC | TTACGCAGAACACCAAAGAAAGA |
| RORA | rs11629812 | ACGTTGGATGATGTGTGGGATACCCACAGG | ACGTTGGATGATACCTCTCAGTCAGCCAAC | CCGGAGCCTCGTTTCTCATTATTA |
| RORA | rs17204698 | ACGTTGGATGGAAGCATGGAAAGGGACATC | ACGTTGGATGGCGGCTTCTGTAAAAGTGTC | GAGAGACATCATGCCATGTGTTAG |
| RORA | rs11071577 | ACGTTGGATGTGCAGGACTTTGGATTAGGC | ACGTTGGATGTGTTGAAATCCAATTTACC | GAGGAGGCAATAGTTTCTTAAGTG |
| RORA | rs2899664 | ACGTTGGATGGGCGGCACTATTAGTGCTAC | ACGTTGGATGAAAAGGAATGACTCTTCTGG | GGGAGTAAGCTTTGGGAAGCTACA |
| RORA | rs12908671 | ACGTTGGATGCTAAACCATACTTTAATCAG | ACGTTGGATGTCCCAGGCCTTAAAATGTCC | TGCACCATACTTTAATCAGCTTAAC |
| RORA | rs1351545 | ACGTTGGATGAGAGGGCTGTCCATTTATAG | ACGTTGGATGCCAGTGAAAACATCTATGGC | GGAGTTCCATTTATAGACTCTCGAC |
| RORA | rs922782 | ACGTTGGATGGAGTCTATACACTAGAAGCTC | ACGTTGGATGCTCTTTGCTCTCTTGCATCC | CCTTGTGACTATTGGATTAAACAGCA |
| RORA | rs3905275 | ACGTTGGATGTTCTGGGCTTGCTATAGAGG | ACGTTGGATGCCCCAACTAAATAAACTGGA | CGGGCTGTAAATGCATTTTCAAGGTT |
| RORA | rs17303097 | ACGTTGGATGTGTGCCCCACGTGAATTATC | ACGTTGGATGTAGCTTAACTTCTCTGAGCC | CCCACGTGAATTATCTTACATTTTACT |
| RORA | rs7183955 | ACGTTGGATGAGCCAACCAGCTATACACAG | ACGTTGGATGGTTTGACTGTGTAAGGTGGC | CCACAAGCTATACACAGGTTCAGGAAA |
| RORA | rs1563565 | ACGTTGGATGTGCTGTGTAACCTCCTAAAC | ACGTTGGATGCCAGGGAAATTATCTCATGC | AAAGGGAAACTTTCTAAAAGAAAGTTA |
| RORA | rs8040151 | ACGTTGGATGCGGTACTTTACTCACTCAGC | ACGTTGGATGAATTGGTCCCCGGTTTCTTG | TTGTTACTTTACTCACTCAGCTTCTAGT |
| RORA | rs7495128 | ACGTTGGATGCACTTTTCCGAATTACTTCC | ACGTTGGATGTGGCCACTAAGCTAACTAAG | GAAGGACTTCCATAAAATCTGTATTCTA |
| RORA | rs9302220 | ACGTTGGATGTGAGAGGAGTACTCTTCTAC | ACGTTGGATGCTTTGTTCAAATCTGTTTC | AAAGGAATGTTCTTATTGTATCACTGCC |
| RORA | rs7342684 | ACGTTGGATGCTTTGCACACGACCTCTAAG | ACGTTGGATGTAGTACCTGTGAAGGTGGTG | CCCCCCGACCTCTAAGCTAGATCATCTAC |
| RORA | rs17237563 | ACGTTGGATGACATAAGAAGAACACACACC | ACGTTGGATGGATTATAAGTGAGGTTCGAC | ACACACACCTGCAAA |
| RORA | rs6494232 | ACGTTGGATGAGTCGATGAGGAAGTCAGTC | ACGTTGGATGATTAAGAGGAAGCTGATGCG | CCCCGAAATATGCCA |
| RORA | rs4775352 | ACGTTGGATGAAGTATTTTCTGGAGGAGGC | ACGTTGGATGGGACAAATTGGACTGGAGAG | AGGAGGCAAAAGGAC |
| RORA | rs718911 | ACGTTGGATGTGAGGATCAACCATTTCACC | ACGTTGGATGGGGATCATCTAGTTGATTCAG | ACCTGGGTCAGGCTGC |
| RORA | rs809736 | ACGTTGGATGACTGGATCTGCATTCCAAGG | ACGTTGGATGTGCCTCACTTCATGGGAATC | CCAAGGTCATCTCTTCC |
| RORA | rs17237570 | ACGTTGGATGCATGGCCCATAAAGCCAAAC | ACGTTGGATGTCTTACTGGTTTCTCACAGC | TTTGCTGACTTCTGCTC |
| RORA | rs7171287 | ACGTTGGATGACCCAGCAGAATAACCCTTG | ACGTTGGATGTGCCCAGGCGAGCAAATGTA | GGCTAGGGAGTTTTACA |
| RORA | rs782920 | ACGTTGGATGTAGGTGTTTGCTGGTCATGG | ACGTTGGATGTTATTCAAGGGCCAAAAGAG | TGGTCATGGTCTCATTAC |
| RORA | rs17204959 | ACGTTGGATGCTAACCTACGAGTGACCAAG | ACGTTGGATGTCTTCCTCGGTGAGTACTTG | AAGGTCAGGGTGAAAAAG |
| RORA | rs3959689 | ACGTTGGATGGGGCACATGGTTTAACCAAA | ACGTTGGATGGCCAAATGCTCCCTTGAAAG | ACACTTCAGTGTCTTCTGG |
| RORA | rs11854760 | ACGTTGGATGCAGATGAGAGTGAAAAGAGG | ACGTTGGATGAAGTGACTCTGAGTGTCAGG | GATTGAAAAGAGGCTGTGA |
| RORA | rs9920962 | ACGTTGGATGAGTCTGTCCTCTGGAAACTG | ACGTTGGATGGAGGCAGCTCCATTTCATAC | AAACGACACAGCTGTTTCTA |
| RORA | rs4775311 | ACGTTGGATGGGTCTAGAAGGATACACACC | ACGTTGGATGACGTATCAGAACAAGGAGGG | GGATACACACCTAGACGTAA |
| RORA | rs2279295 | ACGTTGGATGCATCCATGTTGAAACTGGGC | ACGTTGGATGCTACCACAAAGGACAGCATC | CTAACCAACTTCCCGATTCAA |
| RORA | rs1364822 | ACGTTGGATGGCTTAGGAGAAACTGAGTTC | ACGTTGGATGGGCAGAAATTCCCATGAAGG | CCTCTCCTCTGAAGCTTAACA |
| RORA | rs12442730 | ACGTTGGATGGAGAGTTCCTGTTCTTTCCC | ACGTTGGATGCCACCCTCTCTCTGAAAATC | ATATTTGTGCAGTTTATGTGA |
| RORA | rs4775351 | ACGTTGGATGACTTGCCCTTGCTCAGTTTC | ACGTTGGATGCAGACTCTCCCCTTCTAAAG | GTCATTGCTCAGTTTCTCTGCT |
| RORA | rs17204496 | ACGTTGGATGATTGAAAGAGGTCCCCCTTG | ACGTTGGATGGCATTGCCTGGTGATTATGG | AGCATGCGCTGTTTTAAGGACT |
| RORA | rs16943672 | ACGTTGGATGTGAGGGCAAAAGGGATAAGG | ACGTTGGATGCCCAAACACCATAAGATTCC | AAAAGGGATAAGGATACTAAAA |
| RORA | rs340023 | ACGTTGGATGTTCTGTGGATGGAGGTTGAG | ACGTTGGATGTGCTCACATGTCAAGAGCTG | GGGATGCTTCGAAAATAACTCTG |
| RORA | rs782929 | ACGTTGGATGTACCCTGGGAAGGAAAATGC | ACGTTGGATGATCCTTCCTTCTCAGGGCTT | GTGAAGGAAAATGCCTCAGTTAC |
| RORA | rs1550226 | ACGTTGGATGTAGCTTGGCCTGAGAGTATG | ACGTTGGATGCAAGGTCCGAAATGCTTAGG | TTTGTTGGTGAGGAGTAGGAGAT |
| RORA | rs16943012 | ACGTTGGATGCTTGAAAGACCAAACCAAGG | ACGTTGGATGACATGCAGACGAGGCTGTAG | CCAACGACCAAACCAAGGTAGTTC |
| RORA | rs12439995 | ACGTTGGATGCTCTCTTCTTCCTGATTCCC | ACGTTGGATGAGCCTAGAGAACTACTGGTG | CCCCCCCCATTGATTTTGATTCTCT |
| RORA | rs16943472 | ACGTTGGATGTAAAAATCCCTGTGAGGGCG | ACGTTGGATGTGGTTGATAGTTCCAATGGG | CAAGGTCATTAATAACTCAACTTAG |
| RORA | rs17303530 | ACGTTGGATGGTCAACCTGCTAGGAATTAC | ACGTTGGATGGAATATCAGGATCCATCTGG | GGGACTCTCAAAGCTATTGAGTCTT |
| RORA | rs7169281 | ACGTTGGATGTTCGATCAAAGTCACACAGC | ACGTTGGATGTAGTATGAGGCTCAGGAGTC | GGGAAAGCATGTGGTCAGCTGAGGA |
| RORA | rs10519111 | ACGTTGGATGGGCAAGCATTCCCAAATTAC | ACGTTGGATGGAGAATCTTGCCACCTAGTG | TCCCTCCCAAATTACACAATGAGTAC |
| RORA | rs8037669 | ACGTTGGATGTTCTCCTCAACACAGGGAAG | ACGTTGGATGAGTGCTGGATCTTGATGGTG | CCTTTGAGGAGAAACCATGGAAAAGT |
| RORA | rs12915830 | ACGTTGGATGGCTCTGCTCTAGTAGCTTTG | ACGTTGGATGAGATGAGAAAACTCAGGTCC | CCAGTAAAATCCATTAACCTCATTAGA |
| RORA | rs11638929 | ACGTTGGATGCAGCCGTCTTGACTTCTCTG | ACGTTGGATGTTTAAGCTTTACAGTGCCGC | ACATCGTGGTAGATATAATAAATGCCC |
| RORA | rs6494246 | ACGTTGGATGTGAATCAACCATAAAGGGGC | ACGTTGGATGTCGCCCCTCATTCTAAAGTG | ACCCGAAAGGGGCTTAGAGGTTGGTAC |
| RORA | rs11632858 | ACGTTGGATGGGAAGTTCATCAGGCATCAG | ACGTTGGATGGTGCCCAGCACAATCAATAC | CCAGTCATCAGAAAAACCATTCATTCAT |
| RORA | rs782910 | ACGTTGGATGGGTTTAGATTCCAGAGTAGC | ACGTTGGATGTGCCTAAGTGGCTGCTTTTC | TGGCATATCTGATTTATACTTGATAAAG |
| RORA | rs1916645 | ACGTTGGATGCACCCATGTATATCTTCCAC | ACGTTGGATGTAGAGATGGGTAGATGGGAC | AAGGGATATCTTCCACATGATAGTTATG |
| RORA | rs1437550 | ACGTTGGATGCAACACCACATCTGCTGAAG | ACGTTGGATGTCCGCAGTTTCCGAAAACAC | GGGGCGTTGACTGGACCTGAAATGCTGG |
| RORA | rs7167741 | ACGTTGGATGAGCCTTCATATGCATCTGTC | ACGTTGGATGAGTCACTTAACCAGCATGTC | TTCCTTGCTCAGCAC |
| RORA | rs7162388 | ACGTTGGATGCTAACAATCCTCTTGGGCAC | ACGTTGGATGATTCCCATGAAGTGAGGCAG | AGAACCAGCAAGCAC |
| RORA | rs17191554 | ACGTTGGATGTCTAATGTCCGAGTCCCTTC | ACGTTGGATGCACCGCTGACAATATGCATT | CCTGACCACTCCCTTC |
| RORA | rs16943636 | ACGTTGGATGTGTTTCCAAAAGCAGGGAGG | ACGTTGGATGTTAGCTTTGGTTGATGGGCG | ATGAGCTGGAATCACC |
| RORA | rs103946 | ACGTTGGATGAGATGGATGATCCTTGTGTG | ACGTTGGATGTGTCTGCATACAAGCGGGAG | CGTGGGGACCAACTTG |
| RORA | rs10519085 | ACGTTGGATGATGTTTGTAGACCCAACCAC | ACGTTGGATGGTTACTCTGAGGACCCAATG | GACCCAACCACTTACAT |
| RORA | rs10519108 | ACGTTGGATGCCAGCAGGTTTCTATTAGCC | ACGTTGGATGATGGTGGCAGCAGGATGATT | GGGGCACATGGAACTGA |
| RORA | rs4774376 | ACGTTGGATGCCATTTCCTCCATTAGACCC | ACGTTGGATGCCTAAAACCAGGCCTGTAAC | CCCCTGATACCCACACAG |
| RORA | rs17204770 | ACGTTGGATGGGTTGCTGAGCAGTGAAAAC | ACGTTGGATGTTCCCACAGCAGTGTTTCAG | GCAGTGAAAACATCCTCA |
| RORA | rs7175393 | ACGTTGGATGTGATCTCCTTAAGACTAGGC | ACGTTGGATGATGTAACCTCTTCCTCTGCC | TTAAGACTAGGCTCATACC |
| RORA | rs16943579 | ACGTTGGATGAACATCCCATGGCTTTCCTG | ACGTTGGATGGCTCCCATCTTTTGCTTCAG | GGGGAGTCCACAGTTTGGC |
| RORA | rs17237367 | ACGTTGGATGCCTTCCTCAATACCTAGTGG | ACGTTGGATGTCCTCCACCTTGAGCTTTTC | ACTCCGCTCCCAGAGACAAG |
| RORA | rs1159814 | ACGTTGGATGCAGGAGAGACAGAGTGAGTT | ACGTTGGATGGAAGCTACAAGCTTTTAGGG | TCTGATAGGAGAAAAGAACC |
| RORA | rs12438355 | ACGTTGGATGTGAAAAATGTGCTGTCCCCC | ACGTTGGATGATCAGTCACCACTCCCCTTC | TGCTGTCCCCCTGTTATAATT |
| RORA | rs17204402 | ACGTTGGATGTGGGTCGTGTCACTGAATAC | ACGTTGGATGTGTTCACCTCTACCATCCAG | CATGGAGAATTACTGAGCATT |
| RORA | rs10519099 | ACGTTGGATGGTTAGCGTCTCCAAATGCAG | ACGTTGGATGCCCTCTTGACTAGATGACAC | CTCTAAACTAGTCAGCCTACTT |
| RORA | rs341398 | ACGTTGGATGTGCCTAACCTACTGTTTCCC | ACGTTGGATGGAGATTCTCAGCTTTCCTGC | ACCAATGTTTCCCAGGTTTTCC |
| RORA | rs17270599 | ACGTTGGATGAGGTTTCTCACAACAGACTC | ACGTTGGATGTGCAAAGTCACCAAGGTCTG | GGGGTACTCTGCTGCTGAAAAA |
| RORA | rs12899193 | ACGTTGGATGCTGACTTTACCTCTGGCTTC | ACGTTGGATGTACCACATAGAGTATCCAAG | ACTCCCTTTGTTTTCTTTCCACC |
| RORA | rs12913421 | ACGTTGGATGAGACTCAGTTGTCTAGGAAG | ACGTTGGATGACTAAAAGGACTGTGTCCTC | TAGAAAATATCCCCATTGCAAAA |
| RORA | rs7173461 | ACGTTGGATGAGGAAGCAGATGAGGAAGTC | ACGTTGGATGGATTTTCATCAGTGAAAGGC | CCCCATCTCTCAAAGGCACATCAA |
| RORA | rs341400 | ACGTTGGATGATTACGTCTGGTGTGAGTGG | ACGTTGGATGCTGAAAGACCCCTCTTTCTG | ATAGTGTTCCCCATGAACCTACTG |
| RORA | rs11071564 | ACGTTGGATGTGGTTGTCCAGAAACCCTTC | ACGTTGGATGCTGGTTTTCTGAGGGAAGAG | GGGGCCCAGAAACCCTTCAGAGAA |
| RORA | rs339976 | ACGTTGGATGGAAGGTCAAGACTTTGCCAG | ACGTTGGATGCATGAAAACACAACTTCGGG | CCCCGGTCTGAGAATTCCTAGCTTT |
| RORA | rs17303404 | ACGTTGGATGACGACAGAACCATCCAAACC | ACGTTGGATGAAACACGTGAGTCACCTCTG | GTGGGAATGAGCAACGTTAAAACTC |
| RORA | rs9788704 | ACGTTGGATGCCAGAAGTCAATATTGCACC | ACGTTGGATGGATCAAGGAGTTCTGTCTAC | CCCCCCAATATTGCACCTCTTTGTAT |
| RORA | rs8033151 | ACGTTGGATGGTCTCCTGCAGTTCTAAGAG | ACGTTGGATGGGAGGAGAAACCCACACAAC | AGAGCTAAAGTTTCTCTTATTCTTCA |
| RORA | rs7166448 | ACGTTGGATGCTAGTTCTTTCCTATGGCAG | ACGTTGGATGCACCATGCTAAGGAGTTGAC | TGTTGCTTTCCTATGGCAGTACATCT |
| RORA | rs17270167 | ACGTTGGATGAGCTGATGTGACTGAGAACC | ACGTTGGATGGCTCATGTAGAGACTCCTTC | CCCCTAACCCGCTAGCTAAACTGCACA |
| RORA | rs8041466 | ACGTTGGATGACAGTTTCTTGGGTCTATCC | ACGTTGGATGCAACTGTAAAAGATTGGCCC | ACGTGATCAACAAAAACTCTAGGAATA |
| RORA | rs10438343 | ACGTTGGATGTCTAGATGGTCTTTTCAGGG | ACGTTGGATGCCCCTTGGACCTTTAATGAG | CCCCTACTAAACATCACCTTAGTTAAAG |
| RORA | rs1902618 | ACGTTGGATGACACTGGGTAAAAGGTATGC | ACGTTGGATGAGTTCATCCACAAAGCTTTC | ATTAGCTTTACAACTCTATGCAAATCTA |
| RORA | rs7183068 | ACGTTGGATGTCGCCATTTGATAGCTAAAG | ACGTTGGATGCCTTTAAGTGACCTTATATC | AGGACTATATTTGGGGCTTAATTTCCTC |
| RORA | rs1437541 | ACGTTGGATGGGTTCTCATGGCATTCTCAG | ACGTTGGATGCTCGAAAAACAGAATTCTGC | CCCACTCTCAGCAAAGTCCCCTTCTTCGC |
| RORA | rs7180208 | ACGTTGGATGCCAAGAATTTTGTATCTTGC | ACGTTGGATGGTAGGTCAGGTGTCATAGTG | GAAAGTATCCTTCAAAAGTGAATGAGAA |
| RORA | rs16943117 | ACGTTGGATGCCTGAAATAGGGCTACTGAG | ACGTTGGATGTGTTTTGCAGCAGGATCTGG | GCTACTGAGCAGCTT |
| RORA | rs7177611 | ACGTTGGATGCCAAACCTGCATAGATCTGG | ACGTTGGATGGATACCAGGCACAAGATGGG | GCATGGCTCAAATGC |
| RORA | rs782909 | ACGTTGGATGGTCCAAGATAAGTGGCCACC | ACGTTGGATGAGGTCTCTGTTCTTCTGTGG | CCTTCCTCAAAGCCTG |
| RORA | rs17204475 | ACGTTGGATGTTTAGATGTCACCCCTGTCC | ACGTTGGATGTCTGGACTTCCACATCCTTG | GCAGCCCTGGCGAGAG |
| RORA | rs17191596 | ACGTTGGATGGAAGTGGAAGATGAGTGAGC | ACGTTGGATGCCTCAGTCATTCTTTCACTC | AGTGAGCTTCAATCCCA |
| RORA | rs1523527 | ACGTTGGATGCATTCCCTGAAACCTGAAGC | ACGTTGGATGATGAATTCGGGAGGCATGAC | GGCCTAAGGAGAGAACA |
| RORA | rs17204545 | ACGTTGGATGGTTGCTCCTGATGACTCAAG | ACGTTGGATGTGCTGTTGTCAGGACTCTTG | CCCTTGGTCCACCTCGCA |
| RORA | rs4775294 | ACGTTGGATGAAACATGGCTCTATGCCTTC | ACGTTGGATGTAACAATAATAGTCTGTATG | AGCCTTCATTGAACAACA |
| RORA | rs890156 | ACGTTGGATGACACAATGTCTTCCTTGCCC | ACGTTGGATGGAGATACACAATACAGTGGG | TCTTCCTTGCCCTAAGATG |
| RORA | rs782944 | ACGTTGGATGCCATGTTTTGCCAGAATGGG | ACGTTGGATGAAGAGGAATCTACGTCTGGC | GGGACAAGGGCCATCCTGT |
| RORA | rs2607582 | ACGTTGGATGGTAAACTCAGACACTGCCTC | ACGTTGGATGTCCACTTGGTGAAACTGGTC | CCCTGTCCTCCCTCATAAGT |
| RORA | rs7169364 | ACGTTGGATGAAGGCTGATACTTGGCCTTC | ACGTTGGATGACAGTAGGGAAACTGAAGGC | CTTGCCTTCATGATACGGAG |
| RORA | rs8042149 | ACGTTGGATGAAATGCCAGGTAAAGCCTCG | ACGTTGGATGAGAGGAAAAGTGTCATTCCC | TCAACATTTTTTCCTCAACAA |
| RORA | rs2414687 | ACGTTGGATGGGTAGAGCTTAGGTAACCAC | ACGTTGGATGGTGCTGACACACATATGATT | CCATTATTGCCCAAACAAGAC |
| RORA | rs8036866 | ACGTTGGATGGTGTGTGATACAGATCAGGG | ACGTTGGATGGGTCAAGTGTTAGAGATCAG | GGGATATCAGGGCTCACCGTG |
| RORA | rs12593790 | ACGTTGGATGCTAGCCAAATCCAACTCAAG | ACGTTGGATGCTTCTGTTTTCCTCACACTG | GTGGATCAAGAGAAAGCACCA |
| RORA | rs940221 | ACGTTGGATGGTTTTCTGTTCCCTGTAGCC | ACGTTGGATGACTGTTTTGCCCATACTGCC | ACCCCCTGTAGCCAGTGCCACC |
| RORA | rs1030347 | ACGTTGGATGCTGTATGAGGCCCTCTTTTC | ACGTTGGATGCCCCTCACTTGATTTTGGTC | AAGACTCCAATCCCTGAGAGTT |
| RORA | rs17204635 | ACGTTGGATGCGCTTGTCAAGAGGGCTATG | ACGTTGGATGGGAGCAAGGTTTCTCACAAC | GGGGCAGAGGGCTATGCAAAGT |
| RORA | rs12912233 | ACGTTGGATGAACAGTGTTGCTTGCCCTTC | ACGTTGGATGTTCAGCCAGTGAAGAAAGGG | AAGGTCCCCATCCTCTTTTCAAC |
| RORA | rs2899663 | ACGTTGGATGCAGTAGTAGCATAGAAAATG | ACGTTGGATGTCAGCTCACACCTTGGGAAC | AAGAAAATGAATCTGATTCACTA |
| RORA | rs11630227 | ACGTTGGATGACCTAAATCTCAGCTTTCTC | ACGTTGGATGGTGCTAAGTACTTTAATGCG | TTTATAAATTGGGACTAACAGAA |
| RORA | rs8024629 | ACGTTGGATGTTTAACTCGGAGACTGGGAC | ACGTTGGATGAAATCCTTTGGCATTCGTTC | AACCCTGAGCCTAGTATTTTTCCT |
| RORA | rs782957 | ACGTTGGATGGGATATTCTCCTGCATATAC | ACGTTGGATGATGGGTGAAACTTGAACGGG | GCATATACATAATACAACCATCAA |
| RORA | rs341408 | ACGTTGGATGGAGGTACCATGATGATGCTG | ACGTTGGATGATGATGTGCCCATGCTTGTC | TGTAACATGATGATGCTGTGGAGA |
| RORA | rs10431795 | ACGTTGGATGAAGACACAGGAAGCTCTTGG | ACGTTGGATGTAAAATTTCCACAGAGAGG | CCTCTGAGATAATGCAGCTCTAAAG |
| RORA | rs782934 | ACGTTGGATGGAGTCATCTTCGGATGAGTG | ACGTTGGATGTTTCACACACACCCGTATGC | TGGGCGTGGCTTTGCTAGAAGGTAC |
| RORA | rs2062094 | ACGTTGGATGAACTACAGATGCAGGACACC | ACGTTGGATGAAGGCCCAATCCAGACCTTC | CACGTAGTTCTTTTCTTGCATTAAAC |
| RORA | rs17191442 | ACGTTGGATGAAACTGTGGCCACTTATCCC | ACGTTGGATGCTGCCCTCTACTGGTATGTC | GCTCCCTATTTAAAACAGCCAATAAA |
| RORA | rs2011857 | ACGTTGGATGGGTCCAAAACAGAGCTGATG | ACGTTGGATGGAGGGCATCTCTTGATCATC | GTCAAAAACAGAGCTGATGATAGAAG |
| RORA | rs782907 | ACGTTGGATGACAAACAAACCCTGAGCCAC | ACGTTGGATGACCTCTTACCCAGCTAACTC | TCCCCACAAACCCTGAGCCACTCATGC |
| RORA | rs7495991 | ACGTTGGATGGCAATTACTTTTGTGCCAACC | ACGTTGGATGCCCTCTTCACTTCTCTTATG | CCAACCTAATAATAATATGTTAAAATG |
| RORA | rs1482058 | ACGTTGGATGCTACTGTTCCCCAAAGGAAG | ACGTTGGATGCAAGTAACACTACCACTGGG | GCCAAAATACTAGCAATACCAAATGTG |
| RORA | rs919000 | ACGTTGGATGGGTTTATCTGTGGCTTGGTG | ACGTTGGATGAGTCGCAGAATGAATCAGCC | CCCACCAGTGCTTTGTGCCATTACTTGC |
| RORA | rs4775287 | ACGTTGGATGGTCACCTAACATAGTGCCTG | ACGTTGGATGATCTCAGGCTTGCCATCTAC | CCTTAACCTAACATAGTGCCTGACTAAC |
| RORA | rs1680446 | ACGTTGGATGGGACATTATCTTAGAGTAAGG | ACGTTGGATGCTGGTCTGTATCTAACTGGG | GGGCTCTTTGAATATATTTAGCTTCGTG |
| RORA | rs1871858 | ACGTTGGATGAAGTGCACTGAGTTTACGGG | ACGTTGGATGTGTCTGTGGAATGTGGGTTG | GAGTGGATAATAAGAGAGATATCCATTC |
| RORA | rs17204910 | ACGTTGGATGAACCCTGAAAATGTACTTCC | ACGTTGGATGCCATCAGTTTTTGACAAGGG | CCCACAGCTAATCCC |
| RORA | rs11629597 | ACGTTGGATGCCAAGGAAAGCCTCCTTTTG | ACGTTGGATGGGAGTGAGATGGGAGACTTA | GTGCTGTTAGGTCCC |
| RORA | rs11634318 | ACGTTGGATGACTGAAGAAAGTGGCTGTCC | ACGTTGGATGGGAGCAACGAGGTTATTTCC | ATCGCCTTTTGCAGAA |
| RORA | rs11071570 | ACGTTGGATGAGTGACTCTGAGTGTCAGG | ACGTTGGATGAGTGAAAAGAGGCTGTGACG | CAGGGGCCTAGATGAC |
| RORA | rs12902142 | ACGTTGGATGACAAAAGCTATCAGGCAGGG | ACGTTGGATGAATCAGTGGTCTCCAGCTAC | ACAGGCAGGGGTGAAT |
| RORA | rs16942816 | ACGTTGGATGACTGGGTGGCTTCTCTTCTC | ACGTTGGATGGATAGGCAAACCTTCAGGAC | CCCTTCTCCACGGAGCC |
| RORA | rs8027829 | ACGTTGGATGTCTCTCTGCTTCAAGGACAC | ACGTTGGATGAGAGAGTGGGAGTAAAAGGG | AAAAGTCACCCTGTTTTC |
| RORA | rs10519068 | ACGTTGGATGAAAAGGAGCAGAAGAGCACG | ACGTTGGATGCTGGGGAGAAAGAAAGGAAG | AGCCTGGTTGTGAAGTCT |
| RORA | rs16943448 | ACGTTGGATGTGACCTCTCTAACTGAAGCC | ACGTTGGATGCTTGGAAACTACAGACTGGG | TGCCTATTACCTCCTTGAA |
| RORA | rs12909890 | ACGTTGGATGAACTGGTTCCATGGTGCTTC | ACGTTGGATGTATGACTGACCCAGTGGAGG | CCATGGTGCTTCAGTAAAG |
| RORA | rs8027032 | ACGTTGGATGGAAAGCCCACCAACATCATC | ACGTTGGATGCCTGGCATGCATTTACTCAC | GGGGAATTGGGTTGCAGCC |
| RORA | rs975501 | ACGTTGGATGAGACTCTCTGATGGGAACTC | ACGTTGGATGGCTTCCACCAGAAATCCAAC | CTGCAGACCTTCAGTAACAC |
| RORA | rs6494229 | ACGTTGGATGTGCAGCTTAGTGCAACTTGG | ACGTTGGATGCCCACCTGAAAGTTGTGTGC | GATTGCAACTTGGTGACAAA |
| RORA | rs880626 | ACGTTGGATGGTAAAGCAAAATGGAGAGGG | ACGTTGGATGCTCATCTCCTGCTAGAATGC | GAGAGCGAATGTGGATAAGC |
| RORA | rs16943311 | ACGTTGGATGTCCAATGCGTCTGTGTTGTG | ACGTTGGATGAAGTTGCCACCTGTGCAATC | ACCCCCTGCCTTGTAAAGGAA |
| RORA | rs17204938 | ACGTTGGATGCTAGTGCTACTGGTGTTTGG | ACGTTGGATGGAATACGGCCATATGGTAAG | CTCCTGGAGTATGCAGACATC |
| RORA | rs11635314 | ACGTTGGATGATGGTGGCAATCTCAGTTGG | ACGTTGGATGAATAACTCTGACTTCCCGCC | AGGACAGGAACCTAAGGTAGA |
| RORA | rs880625 | ACGTTGGATGGGTGCCTGGCATATAGATAC | ACGTTGGATGACTAGATGTGTGGCTTTGGG | GAAAAACTTGTTAAGTGAATGA |
| RORA | rs4775318 | ACGTTGGATGATGCACTAGGCATGAACCAC | ACGTTGGATGATGTGGAGGAAAAGCCAAGG | CCCTTCCCCAAATACAAAACTGG |
| RORA | rs17204628 | ACGTTGGATGCCTCTTACATTGCTCTTTCC | ACGTTGGATGATGTCTCTCATGTCCTGGTG | CCCTGACTTTTGAAAGTGACAGA |
| RORA | rs12592311 | ACGTTGGATGGTTCATCTGGATGTCTTCCG | ACGTTGGATGAGTACAGAGTCACAGCAACC | GGTGACTAGAACAGTGAATGAAT |
| RORA | rs7177878 | ACGTTGGATGAGTGCCAAGATTTCAGAAGG | ACGTTGGATGGCCAAGGGAAAGTCACTAAC | TCCTTTTTCAGAAGGATCATCTCA |
| RORA | rs341399 | ACGTTGGATGACCTTCACATGCCAAATGCC | ACGTTGGATGCCTGGATATTTGGACACCTC | CCCACATGCAAAGCGGACAACTCA |
| RORA | rs782905 | ACGTTGGATGGGGAAATGAACTAGATACAGC | ACGTTGGATGTTGGTCCCAAGCTTGTAAAC | GAAGCTTATAAAAAGGCAGGAAAG |
| RORA | rs8040930 | ACGTTGGATGCCAGGCATTTTGCATAAGTC | ACGTTGGATGGAGCACAAATTATACACAGC | GTACTGTAATAATCTCAACAGTAAC |
| RORA | rs4775355 | ACGTTGGATGTGCACTTTGGATTCTGCCTG | ACGTTGGATGCCTGAAAGCATCCAAATGGG | CAAGTCTGCCTGGAATTTTAATAAC |
| RORA | rs340002 | ACGTTGGATGTGGTAATCAGCAGGAATGTG | ACGTTGGATGAGAGAGCTGTCAGTCATTTC | GGGGAATTGATGCTTGTAAAAGAGG |
| RORA | rs8041061 | ACGTTGGATGTGATGAATCGACTCTCCACC | ACGTTGGATGACTCTTAGACATCTATACCC | GGTTCTCATCAGCAAGGTAAACTATG |
| RORA | rs7173227 | ACGTTGGATGGCCTTACTGACTAGAAGTCC | ACGTTGGATGGTGAGGTATGAATTACTCCC | CCCTCTGCTCGCTACCTAAACATTCTC |
| RORA | rs8027234 | ACGTTGGATGATTCACTGGTGGACTGATGG | ACGTTGGATGAAACATGGCCGTGGAAATGG | CTCATCCTGGACTAGTCTTCAACACAC |
| RORA | rs10519100 | ACGTTGGATGCCTCCATGGATGAAATGCTC | ACGTTGGATGTCTAGTCAAGAGGGCCAATC | CAATTCTCACTTTGCGGAGTTTAAACC |
| RORA | rs4775330 | ACGTTGGATGGGCTGTTAGAATGCTGTGTG | ACGTTGGATGGGAGGATAGGAAGATCAAGG | AAGTGAATGTTATATGCATATACATAC |
| RORA | rs2140441 | ACGTTGGATGAGTACTCATCTTAAGGACTC | ACGTTGGATGGGGCCTACTAAGATACTTTTC | CTAACTAGCTATTACTGTTACAATGATT |
| RORA | rs11071588 | ACGTTGGATGTGTCTCTCGCTAGTCCTAAG | ACGTTGGATGGACTGGAATGATTTAAAGCC | TTTGTATTTTATAACAGGGCAGAAGATT |
| RORA | rs726913 | ACGTTGGATGGCCTGAAACATGAGACAGAC | ACGTTGGATGGCTTCTGTTGGATATATGAG | TCGATAAAAGATATAAAAGAAGTGAAAC |
| RORA | rs339998 | ACGTTGGATGGGAAGCTTTAGGAGAAAGGG | ACGTTGGATGTACCAGCACCTTGAGGAATG | TCCTGACTGCCTACT |
| RORA | rs16943453 | ACGTTGGATGTGAGTTCATTCAGGCCTGTG | ACGTTGGATGCGTCTTCTTTCAGGTCCTTC | AGGCCTGTGGTTACA |
| RORA | rs2306500 | ACGTTGGATGCACCCCTGGTAAGGATTTAG | ACGTTGGATGTTACTTGCTCACCTGCTCAC | GGATGGATCCTGTGG |
| RORA | rs1863270 | ACGTTGGATGCCTGTTTAGTGCTTCTTCCG | ACGTTGGATGCTCCCAGCCTTTATTGTTCC | CCGTCTTCAAAATGCC |
| RORA | rs12439380 | ACGTTGGATGGAACCAGTGTGAACAGAAAC | ACGTTGGATGACCGAAGTTCAACCACCTAC | CTTGGGAAGAGTGAGC |
| RORA | rs2438062 | ACGTTGGATGACTTCATCCACGGTGACTTC | ACGTTGGATGGATGGAGAGGCCAAGAAAAG | TCAGTGAGGGGTGGGG |
| RORA | rs341392 | ACGTTGGATGTACGGATACAGCACCATCAG | ACGTTGGATGCTAGCTGTCTTTGCGATAAC | GCGAATGCCTGCTAACT |
| RORA | rs1002147 | ACGTTGGATGTGCGGAGACACACATCTTTG | ACGTTGGATGGTTGAGCATCTCATGCTTAG | TTGGCCTCCTGGCTGTGT |
| RORA | rs11631055 | ACGTTGGATGTAAGATTGTGGAAGCGGCTG | ACGTTGGATGATGAGCAGGAGCTGGATTTC | CTAGGCTGACAAGGTGCT |
| RORA | rs13329238 | ACGTTGGATGCTCCAGAGAGGATTTGTGAC | ACGTTGGATGTGCAAGTGCTGCTCCATTTC | ACAATGGGAAGCTGAATT |
| RORA | rs782935 | ACGTTGGATGCAGTTTTCCAAAACTGCCCC | ACGTTGGATGTAACAGAGAGGGCTCTCTTC | ATTTTCAAATGCTTCCCTT |
| RORA | rs6494221 | ACGTTGGATGTGCAGTTGTGTAGAGGTTTC | ACGTTGGATGTCAAGGGCTACAGTGTTGAC | CCCTCCAGTGCACCAGAGAC |
| RORA | rs10162630 | ACGTTGGATGCCGCACATCCTAAGCAAGAC | ACGTTGGATGGGTCTCTGGCTGCTTTTTTG | ACAGTCAATTTTCTGAAGTC |
| RORA | rs11630262 | ACGTTGGATGAAACCAGCTGGTTAATGGAC | ACGTTGGATGCCAAATAATGATAGGGCATAC | CTGGTTAATGGACTCAGTGT |
| RORA | rs341459 | ACGTTGGATGTCATGTCTGAGAGGCAGAAG | ACGTTGGATGTACCCCTGCTTTCAGCATCC | CGGGGATGTGACATTTGAGC |
| RORA | rs12324380 | ACGTTGGATGAACAGGCCAACACATCTTGC | ACGTTGGATGGACCACCAGCTTGACTTTTG | GCCTATTGCAGCCTTTCACTA |
| RORA | rs12442938 | ACGTTGGATGGAAACTGAGATCTCCCTAAG | ACGTTGGATGTTGGCCCTTGGTAGTAACAC | AGATCTCCCTAAGATCAAATAA |
| RORA | rs17270188 | ACGTTGGATGCCTCTTTTGGAAAGAACTCTC | ACGTTGGATGCCCTGCGAAGACTCTTCATC | GTGGAAAGAACTCTCACTATAT |
| RORA | rs1523531 | ACGTTGGATGGGATGAGTTTCTTACCTAGC | ACGTTGGATGGTACCTGTATGTTCAAAAGCC | ATTTTTCGTATGTGAGAAAAAT |
| RORA | rs2118326 | ACGTTGGATGACCTCCTAAGTGCCAGTTAC | ACGTTGGATGTGTGAACTCCATGACAGCAG | GGTGATGTTCTGAATCCTGGGG |
| RORA | rs10851684 | ACGTTGGATGCTTCGCTGTGAGATGTTGGG | ACGTTGGATGAGTCGTGGTGAACAGTTTGG | GGAGACCAGTGGGGTGGCTGGC |
| RORA | rs2689352 | ACGTTGGATGCCTTGATCACACCTGGTATC | ACGTTGGATGTCCTTAAAGCACGGCTTGAG | CTTCGCACACCTGGTATCATCTAC |
| RORA | rs6494237 | ACGTTGGATGTATAATTTAATTGTTGGTGG | ACGTTGGATGTTGGGCAATAATGTATGTGG | TTCACTCCAATCAGTTTTGAAACA |
| RORA | rs7170465 | ACGTTGGATGCTCTGAAATATGCATACAGTG | ACGTTGGATGTTGGACCCAAAAAAATCCTC | GAGACTTGATCAGTTTTTGGCTTT |
| RORA | rs1370433 | ACGTTGGATGCCATTCTTAGTTGCACAGGG | ACGTTGGATGTGCAAGTGTCTGGAGTAAGG | GGAGTGCATGTGGAAATCTCAAAC |
| RORA | rs7165874 | ACGTTGGATGCCTTCATGGAAAACAATTC | ACGTTGGATGCAGGCAGGTTTTGTTTTGTC | TCCTTACTTCTACATGTGTAGTTTA |
| RORA | rs17303111 | ACGTTGGATGTTTTGTCTTCTGTCATCAC | ACGTTGGATGTTGTCTGGCACATAATAGGG | AACCTTCTTCTGTCATCACTCCTAAA |
| RORA | rs17270578 | ACGTTGGATGGTCAGACCACAAAGTCAAAC | ACGTTGGATGTCCCACTCTGCTATATTGGC | TTTTCAACTCTTTGTTGAGATGCTGT |
| RORA | rs17204573 | ACGTTGGATGCAGGGAAACAGACCTTTGAG | ACGTTGGATGGTTGGACATACTGGTGAGAC | GAACCAAATCTTTCTGTTAGAAATGC |
| RORA | rs3743266 | ACGTTGGATGGGTAACAGCAACTCTTCCAC | ACGTTGGATGAGCCATGATTATTGGCTTCC | TGGATGTCTAGAATTGTGGATAAATA |
| RORA | rs17237521 | ACGTTGGATGCGGCTCAGCAATCTCATTAG | ACGTTGGATGAAGGTACTTTGCTGGAGGTG | GAGTACCTGGGCTGAGTTGTGGATTA |
| RORA | rs9806453 | ACGTTGGATGTACATCCCTTTACCCAACCG | ACGTTGGATGTGTGGTTTGTGGGAATGCTC | CTCACCCATAACATCAATACCAAAAAG |
| RORA | rs340005 | ACGTTGGATGAGTATCCCTTCCACAGTTAC | ACGTTGGATGTGAGACATTTCTATTCCAG | GAAGGTTACATTTCTGATAACCATGAA |
| RORA | rs12440921 | ACGTTGGATGTCTCTTAATAGTTGTGGCTC | ACGTTGGATGATGCATTCTGGCCAGGTATG | GTATTATAATTGCTATTATCATTATCCT |
| RORA | rs7168782 | ACGTTGGATGTTTCCCCTTTTTCCTCTCAG | ACGTTGGATGAACATGGACGTGGTACCTTC | GACCGTTTTCCTCTCAGAAAATTAGTAA |
| RORA | rs1523530 | ACGTTGGATGCATGGGACCCAAGAATTTAC | ACGTTGGATGCTGGAATATGGCCAGATCTC | CTCATGGGGCTAATGAAAATATTTGATT |
| RORB | rs13293006 | ACGTTGGATGGTGGTGTGACAACAGAAAAC | ACGTTGGATGGCTCCTTGTGTATTTTATCC | GAACCAAGGCCTGTT |
| RORB | rs3903529 | ACGTTGGATGCTAAGTCACCTGGGTCATTG | ACGTTGGATGTGTGGTTGGCACAGAACAAG | GGTCATTGGAGAGGG |
| RORB | rs7037043 | ACGTTGGATGTGATGGACCTTTCGTAGAGC | ACGTTGGATGACGCCTTTGCATTTTGTTAG | GAATCAGGGCAATGGC |
| RORB | rs17691363 | ACGTTGGATGCGCCTGTTCTGCTGATACTG | ACGTTGGATGAGATATCTCGGGTCCCATAA | GCTGATACTGCACACTT |
| RORB | rs10781235 | ACGTTGGATGAAACCTTCCTCTATCCAGCC | ACGTTGGATGATGTGATGGGAACCTGACTG | TCGATCAGCTGAGTCAA |
| RORB | rs17612218 | ACGTTGGATGTCAGATAATGGCCTGGCCTC | ACGTTGGATGAAGGTCTGTGGTCAGGATAG | ACGCATTCCGAGAGGGTG |
| RORB | rs10217594 | ACGTTGGATGACGCAAAGACTGTTCTTCGG | ACGTTGGATGCCCTACCTTTGGCTTTAACC | CTCTTCGGTTTCCAGAGAC |
| RORB | rs10869435 | ACGTTGGATGATATGGGATGAGTTGTCCTG | ACGTTGGATGTCGTCACAAACTTACACAGC | GAAAATGCAGGGCAAGAAA |
| RORB | rs1013078 | ACGTTGGATGCTATTCCATTTAGAATACCAC | ACGTTGGATGCTTAACCCTCATTTTGATCCC | GATTTCCGATACTTTCACAT |
| RORB | rs1410225 | ACGTTGGATGATCACAGACTCACAGTGATG | ACGTTGGATGATCTGCAGCAACTTCCAAGC | CTGTTGAAAAAGGTAGTTCC |
| RORB | rs10869410 | ACGTTGGATGCAAAGCTGTCTGACCTCAAG | ACGTTGGATGCCATTGACTAATAATTATGC | AAGGGGTTCAGAAGTTATACT |
| RORB | rs7032677 | ACGTTGGATGATTAGGGCTGTAGCTGTGAC | ACGTTGGATGAGCAAAAGAGTTGACAGGAC | GGTGGTAGCTGTGACTTCAGA |
| RORB | rs7865407 | ACGTTGGATGCATTTGATGAAGGCACGGTC | ACGTTGGATGTCCAAGGAGGCAAAACTACC | CCCTCAGTCACTCAAATAGGGC |
| RORB | rs10869418 | ACGTTGGATGAGTATCCACATTGAAGGGTC | ACGTTGGATGCTGATAGCTTACACATTTC | TATTCAGAAAACACATCAAAGA |
| RORB | rs17227876 | ACGTTGGATGGAGACTGTTATCTTGGAGGG | ACGTTGGATGGGTGTAAATGTAACTCAGCG | CCCGGTATTGCTTCTTAGACATC |
| RORB | rs11144053 | ACGTTGGATGCTTCCTCAGCTGTTGTTTGG | ACGTTGGATGGTAGAGACCTGTCAGTGATG | TTTGGTTACATTTTGGGTTAAGG |
| RORB | rs17684881 | ACGTTGGATGCTGGTTTAGAGTTGGAGATG | ACGTTGGATGGCCACATCCCTTAAATTCTC | GGTGGAGATGATTGTTCGTAGAT |
| RORB | rs11144037 | ACGTTGGATGTGATGGTTCCTTATTGTGTC | ACGTTGGATGTGTTTATGAGGCCTACTGGG | TCATTGTGTCAAATTGTTCCAGAA |
| RORB | rs1570502 | ACGTTGGATGCACACGTATGTGTTTCCAAG | ACGTTGGATGCGAATATATCAGAAGCCATC | CGTGGATTTGATTTTTGATTCTTC |
| RORB | rs7857053 | ACGTTGGATGTTGTGAAAGTGCTTCCTCTG | ACGTTGGATGTAACAATTGAGGTGTACAG | CCCCGATTTTGCTTCAGCAATACTA |
| RORB | rs7033059 | ACGTTGGATGCTGGTCATCCATGGTGGTGA | ACGTTGGATGGGGTGAGAAACAAGTAGAGG | CCTCCATCCATGGTGGTGACCTCTT |
| RORB | rs11144043 | ACGTTGGATGAAAGCTAGCTGTAGGCTAGG | ACGTTGGATGCTTCTATCCTCAGTAAAGGG | GGAGGCTAGGTAATTTCTGACATTA |
| RORB | rs4090240 | ACGTTGGATGGTATATTTCAAACCACTTAG | ACGTTGGATGCACCAAATCTGGGACTTGAG | CTCGTTCAAACCACTTAGAATTTTGG |
| RORB | rs968357 | ACGTTGGATGCCAGGAGAAATACACTGAGG | ACGTTGGATGTCCCAATGTGCTAGCAATTC | GGCGCAAAATAAAAACGGTGCTTTAG |
| RORB | rs1018584 | ACGTTGGATGCTTTTCGTCTTGTTTCCCTC | ACGTTGGATGAGAGGAAGAGGAAGATGCAG | AAATTTTTTTTCCATTTATTTTCAAAG |
| RORB | rs1410226 | ACGTTGGATGTTGGGCAAGACAAGTTGGAG | ACGTTGGATGAGGATATAACAGGAGGTCAG | TAAATTGAATAGTTTTCATTTTTAGAC |
| RORB | rs17691614 | ACGTTGGATGGGCCATGCTCTGTTCTTTAC | ACGTTGGATGACCCACCTCTTCATTTTCCG | CCCCGATCAGATGACATAATCTTGTTTA |
| RORB | rs10869430 | ACGTTGGATGGAGTGAAACAAGACAGTTTG | ACGTTGGATGCACTCCAAACCCATGGCAAC | AGATTGAAACAAGACAGTTTGATTCCAG |
| RORB | rs1410227 | ACGTTGGATGTGGCCCCAGATGCGATTTTG | ACGTTGGATGACATCACTGAGCAGAAGAGG | GAGGCTGCAAGCTGA |
| RORB | rs17612874 | ACGTTGGATGGCAAGGCCATTTAATCTCTC | ACGTTGGATGCTAAAGGCAGTTGATGGGTG | GAGGCAGCTGTGCATT |
| RORB | rs7022435 | ACGTTGGATGGCCAACAGAAATGCCCTTTG | ACGTTGGATGGATTCTGAAGATCGTGCAGG | CCTTTGCCTTTCCCTTC |
| RORB | rs10869412 | ACGTTGGATGATTGTTTGTGCCGATGAATC | ACGTTGGATGTGATAGCCCATTTTGGTTTC | TACAGCTTCTTCACACA |
| RORB | rs12001830 | ACGTTGGATGCCATTTCTCCAAAACATGGG | ACGTTGGATGCCACTGTTTTTCCCTTTGAG | AGCTATGCAGCAGTATTT |
| RORB | rs17611535 | ACGTTGGATGTACAGGCTTCAGATACAGGC | ACGTTGGATGATGTGATGATGCAGCTCCTC | AGATATATGAGCAACAGGA |
| RORB | rs11144033 | ACGTTGGATGTTCCAGACTACACTCTTTTC | ACGTTGGATGCCAAGGCTCTTGGAAATCTG | ATTGCTGTAATTCAGAATCC |
| RORB | rs3750420 | ACGTTGGATGAGCTCTTTGGATACCTCTTC | ACGTTGGATGCCTCATATCTTTGCAGATAGC | GTGCAGATATTTCTGCTGGG |
| RORB | rs11144020 | ACGTTGGATGGGACACAGAGAGGTTGATTC | ACGTTGGATGAGTTTGTGGCATGGGCTTTG | CAGAGAGGTTGATTCTGTTGA |
| RORB | rs10121918 | ACGTTGGATGGCAGTTTTTGCCATTGAAAG | ACGTTGGATGTCCTCATATAAGTATCAAG | CAACCTAATATATTTGCACCAA |
| RORB | rs17060408 | ACGTTGGATGGTGGAGATGGTTAAGATTGG | ACGTTGGATGATTAGCATCAGGATTAGAG | CTAGATCCCTTTACTGAATATG |
| RORB | rs12352112 | ACGTTGGATGGAACTCTAGGAGCAAATTGG | ACGTTGGATGGCAAATTCTATTGCCAACTG | AAGTAAAAATCAGTTATGCCATT |
| RORB | rs1157358 | ACGTTGGATGGCCGCTACAACTTATAAAGTG | ACGTTGGATGGACCAACAAATAACGACAGC | TTACAAGGGGAATATTTAACTTA |
| RORB | rs1327836 | ACGTTGGATGGAGATATTTCCCATTCTGTTG | ACGTTGGATGTTGGGCATCTATGGTACAAC | CCCATTCTGTTGTAAATCCTAACT |
| RORB | rs17612778 | ACGTTGGATGGATAGTTTATAGATGGCTC | ACGTTGGATGCACACAGGCAAAATAGCAAC | TCGTTTATAGATGGCTCTAATACAT |
| RORB | rs11144039 | ACGTTGGATGGTGTGGATCAATATGTAAGAG | ACGTTGGATGAGGTCAATTATATCAGACG | GCTTCAAAATATTATTCTTACACCTT |
