# Supplementary material for: Evidence for genetic association of RORB with bipolar disorder
Source: BMC Psychiatry. 2009 Nov 12;9:70. doi: 10.1186/1471-244X-9-70 (PMC2780413; doi:10.1186/1471-244X-9-70)
Supplement: Additional file 2 — Association results for all analyzed RORA and RORB SNPs. This table details the association results for all RORA and RORB SNPs analyzed and includes results from both case-control and family-based samples. [file 1471-244X-9-70-S2.doc]

Association results for all analyzed *RORA* and *RORB* SNPs

| Gene | SNP | Region | Minor Allele | Major Allele | Case-Control MAF | Case MAF | Control MAF | Case-Control OR | Case-Control *P*-value | TDT MAF (Founders) | T | U | TDT OR | TDT *P*-value | Combined OR | Combined *P*-value |
| --- | --- | --- | --- | --- | --- | --- | --- | --- | --- | --- | --- | --- | --- | --- | --- | --- |
| RORA | rs3743266 | 3' UTR | C | T | 0.368 | 0.406 | 0.339 | 1.335 | 0.103 | 0.349 | 64 | 57 | 1.123 | 0.525 | 1.093 | 0.484 |
| RORA | rs7165874 | 3' UTR | A | T | 0.479 | 0.486 | 0.441 | 1.198 | 0.293 | 0.465 | 76 | 55 | 1.382 | 0.067 | 1.115 | 0.379 |
| RORA | rs1869488 | Intron | C | T | 0.213 | 0.196 | 0.209 | 0.921 | 0.696 | 0.224 | 49 | 40 | 1.225 | 0.340 | 1.026 | 0.864 |
| RORA | rs17270167 | Intron | C | G | 0.176 | 0.180 | 0.151 | 1.233 | 0.362 | 0.145 | 37 | 31 | 1.194 | 0.467 | 1.088 | 0.614 |
| RORA | rs1866006 | Intron | C | T | 0.109 | 0.111 | 0.116 | 0.946 | 0.837 | 0.113 | 28 | 26 | 1.077 | 0.786 | 1.004 | 0.985 |
| RORA | rs17270188 | Intron | G | A | 0.310 | 0.295 | 0.337 | 0.824 | 0.289 | 0.338 | 52 | 69 | 0.754 | 0.122 | 0.902 | 0.425 |
| RORA | rs17191442 | Intron | G | A | 0.109 | 0.090 | 0.097 | 0.922 | 0.780 | 0.115 | 35 | 23 | 1.522 | 0.115 | 1.086 | 0.676 |
| RORA | rs1866007 | Intron | G | A | 0.268 | 0.300 | 0.236 | 1.386 | 0.092 | 0.271 | 58 | 43 | 1.349 | 0.136 | 1.146 | 0.330 |
| RORA | rs17191463 | Intron | G | A | 0.158 | 0.180 | 0.143 | 1.311 | 0.243 | 0.142 | 42 | 32 | 1.313 | 0.245 | 1.125 | 0.475 |
| RORA | rs4594196 | Intron | C | T | 0.180 | 0.143 | 0.194 | 0.696 | 0.111 | 0.194 | 49 | 41 | 1.195 | 0.399 | 0.969 | 0.840 |
| RORA | rs3905275 | Intron | C | A | 0.187 | 0.210 | 0.151 | 1.493 | 0.073 | 0.186 | 35 | 32 | 1.094 | 0.714 | 1.119 | 0.497 |
| RORA | rs17270216 | Intron | G | A | 0.236 | 0.267 | 0.221 | 1.282 | 0.211 | 0.229 | 57 | 42 | 1.357 | 0.132 | 1.127 | 0.399 |
| RORA | rs10851684 | Intron | G | A | 0.461 | 0.490 | 0.406 | 1.404 | 0.048 | 0.423 | 75 | 58 | 1.293 | 0.141 | 1.139 | 0.290 |
| RORA | rs12439380 | Intron | G | C | 0.183 | 0.163 | 0.213 | 0.721 | 0.132 | 0.217 | 54 | 48 | 1.125 | 0.553 | 0.964 | 0.803 |
| RORA | rs10431795 | Intron | T | A | 0.394 | 0.376 | 0.399 | 0.906 | 0.572 | 0.388 | 49 | 66 | 0.742 | 0.113 | 0.921 | 0.518 |
| RORA | rs10851685 | Intron | T | A | 0.130 | 0.148 | 0.147 | 1.003 | 0.990 | 0.117 | 39 | 24 | 1.625 | 0.059 | 1.103 | 0.578 |
| RORA | rs974828 | Intron | T | C | 0.134 | 0.110 | 0.140 | 0.762 | 0.291 | 0.104 | 24 | 23 | 1.043 | 0.884 | 0.943 | 0.763 |
| RORA | rs8036966 | Intron | C | A | 0.130 | 0.131 | 0.147 | 0.872 | 0.576 | 0.133 | 31 | 40 | 0.775 | 0.286 | 0.918 | 0.617 |
| RORA | rs11633266 | Intron | G | A | 0.398 | 0.383 | 0.395 | 0.951 | 0.772 | 0.358 | 74 | 68 | 1.088 | 0.615 | 1.008 | 0.945 |
| RORA | rs340002 | Intron | A | G | 0.220 | 0.207 | 0.211 | 0.975 | 0.902 | 0.216 | 42 | 39 | 1.077 | 0.739 | 1.009 | 0.952 |
| RORA | rs11632600 | Intron | T | G | 0.173 | 0.173 | 0.163 | 1.078 | 0.740 | 0.163 | 36 | 36 | 1.000 | 1.000 | 1.017 | 0.918 |
| RORA | rs2289163 | Intron | C | A | 0.054 | 0.040 | 0.051 | 0.784 | 0.552 | 0.063 | 16 | 7 | 2.286 | 0.061 | 1.109 | 0.734 |
| RORA | rs7172348 | Intron | G | A | 0.176 | 0.187 | 0.190 | 0.979 | 0.922 | 0.207 | 44 | 49 | 0.898 | 0.604 | 0.972 | 0.848 |
| RORA | rs340009 | Intron | A | C | 0.323 | 0.312 | 0.353 | 0.833 | 0.310 | 0.384 | 51 | 80 | 0.638 | 0.011 | 0.871 | 0.278 |
| RORA | rs340021 | Intron | G | C | 0.060 | 0.043 | 0.062 | 0.685 | 0.322 | 0.067 | 10 | 22 | 0.455 | 0.034 | 0.776 | 0.348 |
| RORA | rs16942816 | Intron | C | T | 0.078 | 0.063 | 0.090 | 0.685 | 0.238 | 0.109 | 22 | 31 | 0.710 | 0.216 | 0.856 | 0.461 |
| RORA | rs340023 | Intron | T | C | 0.236 | 0.230 | 0.252 | 0.887 | 0.545 | 0.257 | 46 | 56 | 0.821 | 0.322 | 0.934 | 0.625 |
| RORA | rs103946 | Intron | G | A | 0.137 | 0.103 | 0.155 | 0.628 | 0.068 | 0.177 | 32 | 51 | 0.628 | 0.037 | 0.817 | 0.232 |
| RORA | rs890156 | Intron | A | T | 0.444 | 0.440 | 0.446 | 0.977 | 0.892 | 0.467 | 71 | 64 | 1.109 | 0.547 | 1.017 | 0.887 |
| RORA | rs235512 | Intron | G | A | 0.468 | 0.490 | 0.457 | 1.140 | 0.443 | 0.417 | 67 | 63 | 1.063 | 0.726 | 1.043 | 0.730 |
| RORA | rs11630262 | Promoter/Intron | A | G | 0.208 | 0.200 | 0.182 | 1.122 | 0.594 | 0.210 | 51 | 43 | 1.186 | 0.409 | 1.065 | 0.676 |
| RORA | rs339976 | Intron | C | G | 0.243 | 0.237 | 0.229 | 1.046 | 0.824 | 0.185 | 44 | 41 | 1.073 | 0.745 | 1.025 | 0.867 |
| RORA | rs339996 | Intron | A | G | 0.486 | 0.460 | 0.508 | 0.826 | 0.260 | 0.548 | 56 | 73 | 0.767 | 0.135 | 0.906 | 0.423 |
| RORA | rs17191554 | Intron | T | G | 0.102 | 0.117 | 0.089 | 1.349 | 0.288 | 0.058 | 20 | 16 | 1.250 | 0.505 | 1.123 | 0.591 |
| RORA | rs9806453 | Intron | A | C | 0.280 | 0.269 | 0.316 | 0.793 | 0.215 | 0.339 | 52 | 73 | 0.712 | 0.060 | 0.883 | 0.338 |
| RORA | rs339998 | Intron | A | G | 0.405 | 0.430 | 0.392 | 1.173 | 0.357 | 0.410 | 78 | 48 | 1.625 | 0.008 | 1.146 | 0.280 |
| RORA | rs2438062 | Intron | T | A | 0.279 | 0.274 | 0.248 | 1.144 | 0.492 | 0.224 | 46 | 46 | 1.000 | 1.000 | 1.032 | 0.828 |
| RORA | rs2433026 | Intron | G | C | 0.447 | 0.413 | 0.407 | 1.027 | 0.879 | 0.359 | 67 | 64 | 1.047 | 0.793 | 1.016 | 0.898 |
| RORA | rs1364822 | Intron | C | T | 0.165 | 0.153 | 0.159 | 0.959 | 0.856 | 0.154 | 45 | 38 | 1.184 | 0.442 | 1.031 | 0.851 |
| RORA | rs17303097 | Intron | C | A | 0.180 | 0.230 | 0.186 | 1.307 | 0.204 | 0.194 | 39 | 31 | 1.258 | 0.339 | 1.115 | 0.491 |
| RORA | rs880626 | Intron | T | C | 0.273 | 0.307 | 0.262 | 1.248 | 0.242 | 0.289 | 68 | 57 | 1.193 | 0.325 | 1.090 | 0.510 |
| RORA | rs880625 | Intron | C | T | 0.124 | 0.173 | 0.121 | 1.522 | 0.085 | 0.145 | 31 | 26 | 1.192 | 0.508 | 1.143 | 0.458 |
| RORA | rs12916690 | Intron | G | A | 0.245 | 0.257 | 0.233 | 1.140 | 0.509 | 0.206 | 46 | 47 | 0.979 | 0.917 | 1.026 | 0.860 |
| RORA | rs4775287 | Intron | G | A | 0.306 | 0.343 | 0.295 | 1.252 | 0.219 | 0.326 | 48 | 65 | 0.739 | 0.110 | 0.988 | 0.925 |
| RORA | rs8040332 | Intron | T | A | 0.106 | 0.130 | 0.109 | 1.227 | 0.437 | 0.137 | 31 | 29 | 1.069 | 0.796 | 1.060 | 0.752 |
| RORA | rs919000 | Intron | C | T | 0.197 | 0.203 | 0.182 | 1.146 | 0.528 | 0.172 | 35 | 48 | 0.729 | 0.154 | 0.965 | 0.816 |
| RORA | rs11629812 | Intron | T | C | 0.168 | 0.209 | 0.173 | 1.257 | 0.306 | 0.207 | 34 | 53 | 0.642 | 0.042 | 0.952 | 0.754 |
| RORA | rs6494217 | Intron | T | C | 0.187 | 0.207 | 0.190 | 1.111 | 0.621 | 0.207 | 36 | 52 | 0.692 | 0.088 | 0.946 | 0.716 |
| RORA | rs1425287 | Intron | T | C | 0.208 | 0.217 | 0.217 | 0.998 | 0.991 | 0.210 | 33 | 50 | 0.660 | 0.062 | 0.920 | 0.584 |
| RORA | rs13329238 | Intron | G | T | 0.387 | 0.410 | 0.364 | 1.212 | 0.270 | 0.429 | 78 | 64 | 1.219 | 0.240 | 1.088 | 0.485 |
| RORA | rs4774371 | Intron | T | C | 0.194 | 0.257 | 0.186 | 1.511 | 0.046 | 0.244 | 47 | 48 | 0.979 | 0.918 | 1.088 | 0.565 |
| RORA | rs7181662 | Intron | A | T | 0.367 | 0.426 | 0.343 | 1.423 | 0.046 | 0.430 | 65 | 67 | 0.970 | 0.862 | 1.071 | 0.580 |
| RORA | rs8027032 | Intron | G | A | 0.206 | 0.200 | 0.250 | 0.750 | 0.158 | 0.238 | 55 | 42 | 1.310 | 0.187 | 0.996 | 0.976 |
| RORA | rs8038077 | Intron | G | A | 0.067 | 0.087 | 0.089 | 0.970 | 0.918 | 0.094 | 23 | 21 | 1.095 | 0.763 | 1.013 | 0.952 |
| RORA | rs1030347 | Intron | A | C | 0.312 | 0.329 | 0.275 | 1.291 | 0.170 | 0.338 | 65 | 60 | 1.083 | 0.655 | 1.074 | 0.581 |
| RORA | rs17303111 | Intron | G | A | 0.239 | 0.225 | 0.291 | 0.706 | 0.074 | 0.260 | 58 | 50 | 1.160 | 0.441 | 0.959 | 0.760 |
| RORA | rs7173461 | Intron | G | C | 0.387 | 0.343 | 0.368 | 0.897 | 0.540 | 0.324 | 56 | 63 | 0.889 | 0.521 | 0.952 | 0.700 |
| RORA | rs1863270 | Intron | G | T | 0.324 | 0.332 | 0.287 | 1.237 | 0.249 | 0.338 | 71 | 64 | 1.109 | 0.547 | 1.069 | 0.595 |
| RORA | rs6494219 | Intron | A | G | 0.246 | 0.237 | 0.310 | 0.690 | 0.052 | 0.259 | 54 | 47 | 1.149 | 0.486 | 0.946 | 0.689 |
| RORA | rs17191582 | Intron | C | G | 0.176 | 0.207 | 0.180 | 1.188 | 0.439 | 0.169 | 32 | 35 | 0.914 | 0.714 | 1.024 | 0.887 |
| RORA | rs6494221 | Intron | G | T | 0.170 | 0.203 | 0.195 | 1.052 | 0.814 | 0.214 | 49 | 44 | 1.114 | 0.604 | 1.035 | 0.815 |
| RORA | rs8043356 | Intron | A | G | 0.151 | 0.130 | 0.182 | 0.671 | 0.089 | 0.159 | 45 | 40 | 1.125 | 0.588 | 0.949 | 0.744 |
| RORA | rs12899193 | Intron | G | A | 0.303 | 0.320 | 0.353 | 0.864 | 0.414 | 0.338 | 64 | 61 | 1.049 | 0.788 | 0.979 | 0.867 |
| RORA | rs12909890 | Intron | C | T | 0.369 | 0.367 | 0.422 | 0.793 | 0.184 | 0.371 | 63 | 59 | 1.068 | 0.717 | 0.962 | 0.759 |
| RORA | rs16943012 | Intron | C | G | 0.046 | 0.057 | 0.047 | 1.231 | 0.590 | 0.068 | 15 | 16 | 0.938 | 0.858 | 1.027 | 0.919 |
| RORA | rs8024629 | Intron | T | G | 0.144 | 0.190 | 0.159 | 1.241 | 0.336 | 0.210 | 48 | 45 | 1.067 | 0.756 | 1.060 | 0.702 |
| RORA | rs1820357 | Intron | C | A | 0.460 | 0.472 | 0.453 | 1.080 | 0.656 | 0.438 | 57 | 67 | 0.851 | 0.369 | 0.984 | 0.897 |
| RORA | rs11635314 | Intron | A | G | 0.152 | 0.134 | 0.137 | 0.979 | 0.932 | 0.157 | 34 | 40 | 0.850 | 0.486 | 0.959 | 0.806 |
| RORA | rs341413 | Intron | G | A | 0.208 | 0.187 | 0.182 | 1.030 | 0.891 | 0.200 | 43 | 50 | 0.860 | 0.468 | 0.972 | 0.851 |
| RORA | rs17237367 | Intron | T | C | 0.201 | 0.197 | 0.209 | 0.925 | 0.711 | 0.180 | 36 | 41 | 0.878 | 0.569 | 0.957 | 0.775 |
| RORA | rs17191596 | Intron | G | A | 0.106 | 0.133 | 0.124 | 1.087 | 0.744 | 0.137 | 36 | 32 | 1.125 | 0.628 | 1.045 | 0.802 |
| RORA | rs9920962 | Intron | C | T | 0.130 | 0.133 | 0.140 | 0.949 | 0.831 | 0.121 | 33 | 30 | 1.100 | 0.706 | 1.009 | 0.961 |
| RORA | rs716593 | Intron | A | G | 0.046 | 0.040 | 0.043 | 0.936 | 0.876 | 0.038 | 9 | 13 | 0.692 | 0.394 | 0.911 | 0.759 |
| RORA | rs8025324 | Intron | T | C | 0.137 | 0.147 | 0.143 | 1.027 | 0.913 | 0.116 | 32 | 30 | 1.067 | 0.800 | 1.020 | 0.912 |
| RORA | rs1871858 | Intron | G | C | 0.046 | 0.040 | 0.050 | 0.791 | 0.566 | 0.091 | 18 | 25 | 0.720 | 0.286 | 0.880 | 0.604 |
| RORA | rs7183955 | Intron | G | T | 0.187 | 0.190 | 0.186 | 1.026 | 0.905 | 0.164 | 40 | 40 | 1.000 | 1.000 | 1.006 | 0.971 |
| RORA | rs17204402 | Intron | G | C | 0.151 | 0.187 | 0.159 | 1.215 | 0.388 | 0.176 | 46 | 38 | 1.211 | 0.383 | 1.087 | 0.594 |
| RORA | rs1563565 | Intron | A | C | 0.382 | 0.420 | 0.394 | 1.110 | 0.552 | 0.409 | 60 | 66 | 0.909 | 0.593 | 1.003 | 0.983 |
| RORA | rs8040151 | Intron | C | T | 0.331 | 0.373 | 0.337 | 1.171 | 0.374 | 0.356 | 65 | 67 | 0.970 | 0.862 | 1.027 | 0.829 |
| RORA | rs17204440 | Intron | T | G | 0.475 | 0.520 | 0.496 | 1.100 | 0.574 | 0.469 | 73 | 76 | 0.961 | 0.806 | 1.011 | 0.926 |
| RORA | rs7164773 | Intron | A | G | 0.500 | 0.490 | 0.488 | 1.007 | 0.969 | 0.459 | 73 | 68 | 1.074 | 0.674 | 1.017 | 0.886 |
| RORA | rs12438866 | Intron | G | A | 0.349 | 0.365 | 0.361 | 1.015 | 0.933 | 0.341 | 58 | 59 | 0.983 | 0.926 | 1.000 | 0.998 |
| RORA | rs10519068 | Intron | T | C | 0.124 | 0.120 | 0.120 | 0.999 | 0.997 | 0.099 | 23 | 26 | 0.885 | 0.668 | 0.975 | 0.898 |
| RORA | rs10519070 | Intron | G | A | 0.144 | 0.173 | 0.143 | 1.252 | 0.336 | 0.138 | 30 | 44 | 0.682 | 0.104 | 0.968 | 0.843 |
| RORA | rs922782 | Intron | C | A | 0.468 | 0.479 | 0.465 | 1.059 | 0.740 | 0.436 | 67 | 57 | 1.175 | 0.369 | 1.047 | 0.709 |
| RORA | rs7173227 | Intron | A | G | 0.131 | 0.137 | 0.129 | 1.070 | 0.788 | 0.145 | 31 | 45 | 0.689 | 0.108 | 0.929 | 0.669 |
| RORA | rs12324380 | Intron | G | A | 0.032 | 0.023 | 0.027 | 0.857 | 0.775 | 0.032 | 6 | 11 | 0.546 | 0.225 | 0.842 | 0.643 |
| RORA | rs2899662 | Intron | A | G | 0.394 | 0.350 | 0.399 | 0.810 | 0.231 | 0.358 | 60 | 58 | 1.034 | 0.854 | 0.960 | 0.747 |
| RORA | rs1680446 | Intron | A | G | 0.032 | 0.023 | 0.027 | 0.857 | 0.775 | 0.040 | 6 | 12 | 0.500 | 0.157 | 0.824 | 0.599 |
| RORA | rs17204475 | Intron | T | C | 0.123 | 0.097 | 0.132 | 0.705 | 0.191 | 0.127 | 29 | 28 | 1.036 | 0.895 | 0.935 | 0.722 |
| RORA | rs4775297 | Intron | A | G | 0.419 | 0.362 | 0.421 | 0.783 | 0.170 | 0.403 | 61 | 66 | 0.924 | 0.657 | 0.932 | 0.578 |
| RORA | rs16943117 | Intron | A | G | 0.215 | 0.203 | 0.205 | 0.987 | 0.951 | 0.183 | 42 | 48 | 0.875 | 0.527 | 0.969 | 0.832 |
| RORA | rs11632858 | Intron | A | C | 0.025 | 0.060 | 0.019 | 3.230 | 0.016 | 0.026 | 8 | 8 | 1.000 | 1.000 | 1.282 | 0.488 |
| RORA | rs341459 | Intron | G | A | 0.254 | 0.290 | 0.264 | 1.141 | 0.487 | 0.294 | 64 | 44 | 1.455 | 0.054 | 1.115 | 0.426 |
| RORA | rs10162630 | Intron | T | C | 0.472 | 0.497 | 0.469 | 1.117 | 0.514 | 0.471 | 79 | 65 | 1.215 | 0.243 | 1.069 | 0.577 |
| RORA | rs17204489 | Intron | T | C | 0.057 | 0.064 | 0.047 | 1.396 | 0.377 | 0.042 | 9 | 10 | 0.900 | 0.819 | 1.070 | 0.817 |
| RORA | rs12902540 | Intron | G | A | 0.092 | 0.123 | 0.093 | 1.372 | 0.253 | 0.121 | 29 | 36 | 0.806 | 0.385 | 1.010 | 0.958 |
| RORA | rs17204496 | Intron | G | C | 0.039 | 0.037 | 0.027 | 1.365 | 0.525 | 0.036 | 5 | 12 | 0.417 | 0.090 | 0.903 | 0.778 |
| RORA | rs10519076 | Intron | G | C | 0.151 | 0.153 | 0.140 | 1.117 | 0.646 | 0.187 | 36 | 51 | 0.706 | 0.108 | 0.940 | 0.703 |
| RORA | rs7169281 | Intron | G | A | 0.454 | 0.523 | 0.461 | 1.282 | 0.144 | 0.494 | 71 | 50 | 1.420 | 0.056 | 1.137 | 0.305 |
| RORA | rs341380 | Intron | A | C | 0.366 | 0.300 | 0.368 | 0.735 | 0.088 | 0.295 | 48 | 50 | 0.960 | 0.840 | 0.921 | 0.541 |
| RORA | rs17270501 | Intron | A | G | 0.029 | 0.014 | 0.027 | 0.487 | 0.246 | 0.042 | 6 | 10 | 0.600 | 0.317 | 0.773 | 0.519 |
| RORA | rs12439995 | Intron | C | G | 0.165 | 0.163 | 0.143 | 1.166 | 0.516 | 0.151 | 33 | 37 | 0.892 | 0.633 | 1.009 | 0.956 |
| RORA | rs12440095 | Intron | T | C | 0.123 | 0.133 | 0.089 | 1.572 | 0.100 | 0.124 | 26 | 31 | 0.839 | 0.508 | 1.056 | 0.776 |
| RORA | rs341390 | Intron | T | C | 0.324 | 0.277 | 0.326 | 0.792 | 0.208 | 0.258 | 43 | 49 | 0.878 | 0.532 | 0.922 | 0.556 |
| RORA | rs7175883 | Intron | T | C | 0.050 | 0.030 | 0.062 | 0.468 | 0.068 | 0.030 | 7 | 7 | 1.000 | 1.000 | 0.817 | 0.544 |
| RORA | rs341392 | Intron | G | T | 0.222 | 0.203 | 0.217 | 0.921 | 0.691 | 0.237 | 47 | 55 | 0.855 | 0.428 | 0.949 | 0.713 |
| RORA | rs6494229 | Intron | T | C | 0.486 | 0.423 | 0.461 | 0.857 | 0.368 | 0.439 | 49 | 72 | 0.681 | 0.037 | 0.893 | 0.368 |
| RORA | rs7181803 | Intron | G | A | 0.050 | 0.030 | 0.062 | 0.468 | 0.068 | 0.030 | 7 | 7 | 1.000 | 1.000 | 0.817 | 0.544 |
| RORA | rs8041061 | Intron | A | C | 0.457 | 0.530 | 0.441 | 1.430 | 0.037 | 0.508 | 65 | 55 | 0.846 | 0.361 | 1.050 | 0.695 |
| RORA | rs8042149 | Intron | A | C | 0.475 | 0.416 | 0.485 | 0.758 | 0.106 | 0.458 | 60 | 71 | 0.845 | 0.337 | 0.907 | 0.428 |
| RORA | rs4775301 | Intron | G | A | 0.486 | 0.553 | 0.481 | 1.339 | 0.087 | 0.531 | 81 | 60 | 0.741 | 0.077 | 0.998 | 0.988 |
| RORA | rs341398 | Intron | T | C | 0.447 | 0.387 | 0.434 | 0.822 | 0.256 | 0.351 | 53 | 65 | 0.815 | 0.269 | 0.917 | 0.492 |
| RORA | rs341399 | Intron | A | G | 0.149 | 0.117 | 0.152 | 0.735 | 0.217 | 0.156 | 29 | 44 | 0.659 | 0.079 | 0.853 | 0.359 |
| RORA | rs341400 | Intron | T | C | 0.046 | 0.043 | 0.054 | 0.789 | 0.549 | 0.056 | 14 | 15 | 0.933 | 0.853 | 0.938 | 0.813 |
| RORA | rs3959689 | Intron | G | C | 0.496 | 0.570 | 0.492 | 1.367 | 0.066 | 0.564 | 77 | 52 | 0.675 | 0.028 | 0.990 | 0.938 |
| RORA | rs11630062 | Intron | G | A | 0.113 | 0.077 | 0.112 | 0.656 | 0.148 | 0.104 | 25 | 32 | 0.781 | 0.354 | 0.868 | 0.473 |
| RORA | rs341403 | Intron | G | A | 0.268 | 0.262 | 0.284 | 0.897 | 0.572 | 0.242 | 54 | 45 | 1.200 | 0.366 | 1.013 | 0.926 |
| RORA | rs754499 | Intron | C | A | 0.338 | 0.367 | 0.349 | 1.081 | 0.662 | 0.405 | 63 | 70 | 0.900 | 0.544 | 0.993 | 0.956 |
| RORA | rs11630227 | Intron | G | A | 0.447 | 0.410 | 0.419 | 0.965 | 0.837 | 0.380 | 57 | 56 | 1.018 | 0.925 | 0.995 | 0.970 |
| RORA | rs341408 | Intron | A | G | 0.419 | 0.410 | 0.423 | 0.950 | 0.766 | 0.390 | 69 | 63 | 1.095 | 0.602 | 1.008 | 0.947 |
| RORA | rs17204545 | Intron | C | A | 0.202 | 0.185 | 0.190 | 0.965 | 0.872 | 0.169 | 39 | 34 | 1.147 | 0.558 | 1.020 | 0.903 |
| RORA | rs11631055 | Intron | A | G | 0.113 | 0.133 | 0.132 | 1.014 | 0.957 | 0.156 | 42 | 39 | 1.077 | 0.739 | 1.021 | 0.901 |
| RORA | rs1673336 | Intron | A | G | 0.173 | 0.140 | 0.167 | 0.814 | 0.382 | 0.135 | 36 | 19 | 1.895 | 0.022 | 1.063 | 0.738 |
| RORA | rs2607582 | Intron | G | A | 0.099 | 0.083 | 0.101 | 0.811 | 0.476 | 0.065 | 17 | 15 | 1.133 | 0.724 | 0.969 | 0.889 |
| RORA | rs2306502 | Intron | G | T | 0.099 | 0.080 | 0.085 | 0.933 | 0.821 | 0.081 | 25 | 13 | 1.923 | 0.052 | 1.117 | 0.629 |
| RORA | rs2306500 | Intron | C | G | 0.327 | 0.317 | 0.306 | 1.050 | 0.790 | 0.285 | 55 | 53 | 1.038 | 0.847 | 1.019 | 0.887 |
| RORA | rs10519085 | Intron | A | G | 0.444 | 0.483 | 0.488 | 0.980 | 0.906 | 0.506 | 69 | 71 | 0.972 | 0.866 | 0.989 | 0.929 |
| RORA | rs8041466 | Intron | A | G | 0.394 | 0.387 | 0.361 | 1.119 | 0.524 | 0.346 | 59 | 52 | 1.135 | 0.506 | 1.053 | 0.689 |
| RORA | rs1482058 | Intron | G | A | 0.486 | 0.466 | 0.519 | 0.809 | 0.213 | 0.496 | 58 | 69 | 0.841 | 0.329 | 0.919 | 0.494 |
| RORA | rs1902618 | Intron | C | T | 0.206 | 0.211 | 0.213 | 0.990 | 0.959 | 0.225 | 49 | 53 | 0.925 | 0.692 | 0.980 | 0.889 |
| RORA | rs7166448 | Intron | T | C | 0.110 | 0.091 | 0.109 | 0.811 | 0.461 | 0.093 | 20 | 20 | 1.000 | 1.000 | 0.951 | 0.812 |
| RORA | rs17204573 | Intron | T | C | 0.085 | 0.087 | 0.098 | 0.877 | 0.655 | 0.094 | 22 | 26 | 0.846 | 0.564 | 0.937 | 0.753 |
| RORA | rs4775310 | Intron | G | C | 0.120 | 0.128 | 0.124 | 1.032 | 0.902 | 0.135 | 35 | 40 | 0.875 | 0.564 | 0.974 | 0.881 |
| RORA | rs341387 | Intron | A | G | 0.440 | 0.440 | 0.461 | 0.918 | 0.615 | 0.473 | 59 | 73 | 0.808 | 0.223 | 0.938 | 0.600 |
| RORA | rs8027829 | Intron | T | C | 0.418 | 0.420 | 0.426 | 0.977 | 0.891 | 0.441 | 57 | 66 | 0.864 | 0.417 | 0.965 | 0.775 |
| RORA | rs11631432 | Intron | A | G | 0.496 | 0.497 | 0.488 | 1.034 | 0.845 | 0.529 | 63 | 57 | 0.905 | 0.584 | 0.988 | 0.921 |
| RORA | rs4775311 | Intron | G | A | 0.197 | 0.190 | 0.209 | 0.886 | 0.569 | 0.188 | 43 | 43 | 1.000 | 1.000 | 0.974 | 0.860 |
| RORA | rs8039843 | Intron | T | G | 0.201 | 0.205 | 0.190 | 1.098 | 0.663 | 0.175 | 32 | 37 | 0.865 | 0.547 | 0.995 | 0.975 |
| RORA | rs113168 | Intron | G | C | 0.306 | 0.299 | 0.326 | 0.882 | 0.494 | 0.343 | 56 | 68 | 0.824 | 0.281 | 0.933 | 0.588 |
| RORA | rs12913421 | Intron | T | C | 0.282 | 0.270 | 0.275 | 0.974 | 0.891 | 0.278 | 60 | 40 | 1.500 | 0.046 | 1.079 | 0.586 |
| RORA | rs11071564 | Intron | T | A | 0.229 | 0.223 | 0.240 | 0.909 | 0.635 | 0.235 | 56 | 49 | 1.143 | 0.495 | 1.010 | 0.945 |
| RORA | rs7172917 | Intron | A | G | 0.222 | 0.223 | 0.244 | 0.890 | 0.561 | 0.233 | 47 | 52 | 0.904 | 0.615 | 0.954 | 0.739 |
| RORA | rs12592999 | Intron | T | C | 0.287 | 0.272 | 0.324 | 0.778 | 0.178 | 0.319 | 51 | 56 | 0.911 | 0.629 | 0.927 | 0.571 |
| RORA | rs4774376 | Intron | C | G | 0.067 | 0.093 | 0.074 | 1.295 | 0.404 | 0.079 | 20 | 14 | 1.429 | 0.304 | 1.140 | 0.571 |
| RORA | rs7168782 | Intron | T | G | 0.157 | 0.147 | 0.165 | 0.868 | 0.545 | 0.167 | 40 | 42 | 0.952 | 0.825 | 0.961 | 0.803 |
| RORA | rs17270578 | Intron | G | A | 0.101 | 0.097 | 0.116 | 0.822 | 0.479 | 0.110 | 24 | 31 | 0.774 | 0.345 | 0.906 | 0.612 |
| RORA | rs2899663 | Intron | T | C | 0.437 | 0.433 | 0.500 | 0.763 | 0.114 | 0.481 | 60 | 62 | 0.968 | 0.856 | 0.934 | 0.580 |
| RORA | rs2414686 | Intron | T | C | 0.317 | 0.332 | 0.368 | 0.854 | 0.374 | 0.363 | 59 | 54 | 1.093 | 0.638 | 0.982 | 0.889 |
| RORA | rs17204628 | Intron | A | T | 0.099 | 0.093 | 0.106 | 0.873 | 0.633 | 0.110 | 27 | 31 | 0.871 | 0.599 | 0.942 | 0.758 |
| RORA | rs17204635 | Intron | A | G | 0.025 | 0.030 | 0.023 | 1.299 | 0.623 | 0.024 | 8 | 6 | 1.333 | 0.593 | 1.127 | 0.754 |
| RORA | rs17270599 | Intron | C | T | 0.095 | 0.083 | 0.105 | 0.778 | 0.388 | 0.104 | 24 | 28 | 0.857 | 0.579 | 0.917 | 0.665 |
| RORA | rs2899664 | Intron | T | C | 0.387 | 0.383 | 0.361 | 1.103 | 0.578 | 0.375 | 58 | 71 | 0.817 | 0.252 | 0.978 | 0.859 |
| RORA | rs2062091 | Intron | G | A | 0.380 | 0.400 | 0.384 | 1.071 | 0.695 | 0.385 | 58 | 66 | 0.879 | 0.473 | 0.988 | 0.925 |
| RORA | rs11638433 | Intron | G | C | 0.296 | 0.270 | 0.283 | 0.937 | 0.733 | 0.308 | 45 | 57 | 0.790 | 0.235 | 0.938 | 0.644 |
| RORA | rs8027234 | Intron | C | G | 0.189 | 0.175 | 0.211 | 0.791 | 0.277 | 0.215 | 50 | 42 | 1.190 | 0.404 | 0.990 | 0.945 |
| RORA | rs4378570 | Intron | A | G | 0.362 | 0.305 | 0.372 | 0.742 | 0.097 | 0.323 | 67 | 62 | 1.081 | 0.660 | 0.955 | 0.714 |
| RORA | rs4775314 | Intron | G | A | 0.232 | 0.243 | 0.221 | 1.134 | 0.533 | 0.264 | 48 | 55 | 0.873 | 0.490 | 0.997 | 0.981 |
| RORA | rs16943311 | Intron | C | G | 0.321 | 0.342 | 0.316 | 1.124 | 0.519 | 0.315 | 57 | 52 | 1.096 | 0.632 | 1.047 | 0.730 |
| RORA | rs6494232 | Intron | C | T | 0.113 | 0.063 | 0.109 | 0.555 | 0.055 | 0.089 | 23 | 24 | 0.958 | 0.884 | 0.878 | 0.542 |
| RORA | rs8036866 | Intron | T | C | 0.085 | 0.077 | 0.085 | 0.891 | 0.710 | 0.098 | 22 | 26 | 0.846 | 0.564 | 0.940 | 0.769 |
| RORA | rs16943318 | Intron | T | C | 0.275 | 0.247 | 0.244 | 1.013 | 0.946 | 0.264 | 71 | 49 | 1.449 | 0.045 | 1.092 | 0.515 |
| RORA | rs4775318 | Intron | T | A | 0.319 | 0.303 | 0.332 | 0.876 | 0.468 | 0.306 | 52 | 69 | 0.754 | 0.122 | 0.914 | 0.487 |
| RORA | rs8033151 | Intron | T | C | 0.306 | 0.300 | 0.275 | 1.129 | 0.519 | 0.308 | 72 | 49 | 1.469 | 0.037 | 1.117 | 0.401 |
| RORA | rs2062094 | Intron | G | A | 0.243 | 0.252 | 0.250 | 1.009 | 0.964 | 0.228 | 39 | 60 | 0.650 | 0.035 | 0.916 | 0.537 |
| RORA | rs7166062 | Intron | G | A | 0.053 | 0.080 | 0.047 | 1.783 | 0.108 | 0.083 | 24 | 17 | 1.412 | 0.274 | 1.213 | 0.418 |
| RORA | rs11854760 | Intron | G | C | 0.356 | 0.310 | 0.310 | 1.000 | 0.998 | 0.319 | 70 | 53 | 1.321 | 0.125 | 1.063 | 0.638 |
| RORA | rs11634318 | Intron | A | C | 0.064 | 0.063 | 0.047 | 1.375 | 0.399 | 0.051 | 12 | 14 | 0.857 | 0.695 | 1.040 | 0.885 |
| RORA | rs2279291 | Intron | A | C | 0.451 | 0.453 | 0.500 | 0.829 | 0.271 | 0.463 | 61 | 78 | 0.782 | 0.149 | 0.910 | 0.436 |
| RORA | rs12442938 | Intron | G | C | 0.158 | 0.197 | 0.159 | 1.296 | 0.246 | 0.189 | 31 | 41 | 0.756 | 0.239 | 1.003 | 0.986 |
| RORA | rs1351546 | Intron | A | G | 0.380 | 0.413 | 0.407 | 1.027 | 0.879 | 0.409 | 59 | 77 | 0.766 | 0.123 | 0.949 | 0.671 |
| RORA | rs1351545 | Intron | A | G | 0.239 | 0.243 | 0.236 | 1.039 | 0.849 | 0.235 | 53 | 45 | 1.178 | 0.419 | 1.044 | 0.760 |
| RORA | rs7342684 | Intron | C | A | 0.175 | 0.184 | 0.201 | 0.894 | 0.615 | 0.179 | 40 | 41 | 0.976 | 0.912 | 0.971 | 0.851 |
| RORA | rs12438355 | Intron | T | C | 0.204 | 0.227 | 0.198 | 1.190 | 0.405 | 0.230 | 37 | 50 | 0.740 | 0.163 | 0.977 | 0.876 |
| RORA | rs4775330 | Intron | T | C | 0.298 | 0.290 | 0.305 | 0.932 | 0.706 | 0.305 | 61 | 52 | 1.173 | 0.397 | 1.019 | 0.888 |
| RORA | rs12442730 | Intron | C | T | 0.151 | 0.183 | 0.140 | 1.384 | 0.163 | 0.171 | 30 | 46 | 0.652 | 0.066 | 0.979 | 0.897 |
| RORA | rs4332688 | Intron | C | G | 0.451 | 0.460 | 0.457 | 1.011 | 0.950 | 0.473 | 65 | 72 | 0.903 | 0.550 | 0.980 | 0.870 |
| RORA | rs2140441 | Intron | A | T | 0.396 | 0.378 | 0.414 | 0.861 | 0.392 | 0.390 | 61 | 60 | 1.017 | 0.928 | 0.970 | 0.810 |
| RORA | rs17204698 | Intron | G | T | 0.239 | 0.247 | 0.205 | 1.266 | 0.247 | 0.220 | 48 | 44 | 1.091 | 0.677 | 1.073 | 0.627 |
| RORA | rs1523527 | Intron | C | T | 0.296 | 0.287 | 0.333 | 0.804 | 0.234 | 0.304 | 62 | 58 | 1.069 | 0.715 | 0.968 | 0.801 |
| RORA | rs12440921 | Intron | C | G | 0.064 | 0.051 | 0.071 | 0.694 | 0.309 | 0.074 | 22 | 20 | 1.100 | 0.758 | 0.958 | 0.854 |
| RORA | rs8034880 | Intron | C | T | 0.116 | 0.110 | 0.120 | 0.905 | 0.707 | 0.095 | 16 | 24 | 0.667 | 0.206 | 0.908 | 0.636 |
| RORA | rs8034950 | Intron | G | A | 0.335 | 0.317 | 0.349 | 0.865 | 0.421 | 0.320 | 62 | 60 | 1.033 | 0.856 | 0.976 | 0.847 |
| RORA | rs12912233 | Intron | A | G | 0.430 | 0.417 | 0.454 | 0.861 | 0.382 | 0.433 | 69 | 64 | 1.078 | 0.665 | 0.983 | 0.890 |
| RORA | rs17237521 | Intron | G | A | 0.148 | 0.167 | 0.140 | 1.233 | 0.376 | 0.137 | 21 | 38 | 0.553 | 0.027 | 0.942 | 0.738 |
| RORA | rs2414687 | Intron | T | G | 0.218 | 0.217 | 0.252 | 0.821 | 0.326 | 0.263 | 59 | 52 | 1.135 | 0.506 | 0.989 | 0.933 |
| RORA | rs6494237 | Intron | G | A | 0.232 | 0.202 | 0.242 | 0.792 | 0.259 | 0.219 | 59 | 43 | 1.372 | 0.113 | 1.022 | 0.881 |
| RORA | rs4638514 | Intron | A | G | 0.388 | 0.317 | 0.409 | 0.669 | 0.026 | 0.340 | 58 | 50 | 1.160 | 0.441 | 0.939 | 0.632 |
| RORA | rs11071577 | Intron | C | T | 0.415 | 0.379 | 0.426 | 0.822 | 0.258 | 0.431 | 77 | 60 | 1.283 | 0.146 | 1.012 | 0.920 |
| RORA | rs1589702 | Intron | A | G | 0.447 | 0.400 | 0.446 | 0.829 | 0.275 | 0.380 | 71 | 59 | 1.203 | 0.293 | 0.997 | 0.984 |
| RORA | rs7180208 | Intron | G | A | 0.345 | 0.390 | 0.326 | 1.324 | 0.114 | 0.365 | 46 | 63 | 0.730 | 0.104 | 1.004 | 0.977 |
| RORA | rs7495128 | Intron | A | G | 0.236 | 0.201 | 0.248 | 0.764 | 0.187 | 0.198 | 49 | 46 | 1.065 | 0.758 | 0.956 | 0.755 |
| RORA | rs7178442 | Intron | A | G | 0.218 | 0.173 | 0.209 | 0.792 | 0.280 | 0.189 | 46 | 39 | 1.179 | 0.448 | 0.985 | 0.920 |
| RORA | rs16943448 | Intron | C | A | 0.164 | 0.133 | 0.161 | 0.799 | 0.351 | 0.142 | 37 | 32 | 1.156 | 0.547 | 0.983 | 0.919 |
| RORA | rs7176717 | Intron | T | C | 0.482 | 0.440 | 0.473 | 0.876 | 0.437 | 0.402 | 70 | 66 | 1.061 | 0.732 | 0.984 | 0.894 |
| RORA | rs12591914 | Intron | T | G | 0.289 | 0.323 | 0.267 | 1.309 | 0.150 | 0.318 | 54 | 62 | 0.871 | 0.458 | 1.028 | 0.832 |
| RORA | rs16943453 | Intron | C | A | 0.187 | 0.163 | 0.194 | 0.812 | 0.348 | 0.148 | 36 | 34 | 1.059 | 0.811 | 0.964 | 0.820 |
| RORA | rs1110418 | Intron | A | G | 0.355 | 0.293 | 0.363 | 0.728 | 0.079 | 0.292 | 64 | 50 | 1.280 | 0.190 | 0.980 | 0.877 |
| RORA | rs4265751 | Intron | A | C | 0.405 | 0.383 | 0.434 | 0.810 | 0.224 | 0.411 | 64 | 58 | 1.103 | 0.587 | 0.973 | 0.826 |
| RORA | rs951265 | Intron | T | C | 0.429 | 0.414 | 0.430 | 0.939 | 0.717 | 0.357 | 57 | 48 | 1.188 | 0.380 | 1.018 | 0.892 |
| RORA | rs1916645 | Intron | T | C | 0.089 | 0.088 | 0.080 | 1.116 | 0.725 | 0.098 | 16 | 19 | 0.842 | 0.612 | 0.992 | 0.972 |
| RORA | rs10519097 | Intron | A | G | 0.148 | 0.163 | 0.151 | 1.096 | 0.694 | 0.142 | 32 | 39 | 0.821 | 0.406 | 0.978 | 0.896 |
| RORA | rs17204770 | Intron | G | A | 0.454 | 0.540 | 0.454 | 1.415 | 0.042 | 0.504 | 75 | 72 | 0.960 | 0.805 | 1.066 | 0.591 |
| RORA | rs12908671 | Intron | T | C | 0.127 | 0.140 | 0.116 | 1.237 | 0.405 | 0.123 | 26 | 35 | 0.743 | 0.249 | 0.983 | 0.926 |
| RORA | rs16943472 | Intron | G | C | 0.127 | 0.137 | 0.120 | 1.159 | 0.562 | 0.124 | 26 | 33 | 0.788 | 0.362 | 0.983 | 0.925 |
| RORA | rs11638929 | Intron | A | G | 0.486 | 0.443 | 0.535 | 0.693 | 0.031 | 0.486 | 70 | 75 | 0.933 | 0.678 | 0.911 | 0.434 |
| RORA | rs10519099 | Intron | G | A | 0.261 | 0.283 | 0.252 | 1.174 | 0.404 | 0.268 | 59 | 62 | 0.952 | 0.785 | 1.022 | 0.871 |
| RORA | rs10519100 | Intron | G | C | 0.106 | 0.130 | 0.086 | 1.589 | 0.098 | 0.125 | 31 | 24 | 1.292 | 0.345 | 1.167 | 0.429 |
| RORA | rs17237563 | Intron | A | G | 0.127 | 0.140 | 0.128 | 1.110 | 0.676 | 0.142 | 41 | 38 | 1.079 | 0.736 | 1.039 | 0.818 |
| RORA | rs1523530 | Intron | T | A | 0.128 | 0.138 | 0.129 | 1.078 | 0.765 | 0.140 | 40 | 36 | 1.111 | 0.646 | 1.041 | 0.815 |
| RORA | rs16943489 | Intron | G | A | 0.141 | 0.163 | 0.124 | 1.379 | 0.189 | 0.133 | 27 | 38 | 0.711 | 0.172 | 0.999 | 0.997 |
| RORA | rs17237570 | Intron | A | G | 0.070 | 0.067 | 0.081 | 0.806 | 0.506 | 0.067 | 20 | 18 | 1.111 | 0.746 | 0.976 | 0.917 |
| RORA | rs8040930 | Intron | C | T | 0.049 | 0.038 | 0.044 | 0.869 | 0.747 | 0.043 | 12 | 8 | 1.500 | 0.371 | 1.053 | 0.869 |
| RORA | rs4774381 | Intron | C | T | 0.197 | 0.161 | 0.221 | 0.677 | 0.072 | 0.188 | 34 | 36 | 0.944 | 0.811 | 0.901 | 0.519 |
| RORA | rs1403737 | Intron | T | C | 0.296 | 0.237 | 0.322 | 0.654 | 0.025 | 0.282 | 46 | 46 | 1.000 | 1.000 | 0.904 | 0.474 |
| RORA | rs10519105 | Intron | G | C | 0.088 | 0.087 | 0.101 | 0.847 | 0.568 | 0.090 | 19 | 22 | 0.864 | 0.639 | 0.934 | 0.749 |
| RORA | rs1403739 | Intron | C | T | 0.156 | 0.128 | 0.155 | 0.797 | 0.351 | 0.139 | 40 | 27 | 1.481 | 0.112 | 1.034 | 0.848 |
| RORA | rs17270745 | Intron | C | T | 0.102 | 0.087 | 0.097 | 0.891 | 0.694 | 0.104 | 31 | 25 | 1.240 | 0.423 | 1.028 | 0.888 |
| RORA | rs782957 | Intron | A | T | 0.150 | 0.117 | 0.168 | 0.659 | 0.088 | 0.149 | 25 | 31 | 0.807 | 0.423 | 0.868 | 0.436 |
| RORA | rs10519107 | Intron | C | G | 0.465 | 0.463 | 0.500 | 0.863 | 0.387 | 0.504 | 82 | 68 | 1.206 | 0.253 | 1.011 | 0.923 |
| RORA | rs7162388 | Intron | T | C | 0.063 | 0.070 | 0.070 | 1.004 | 0.991 | 0.062 | 12 | 15 | 0.800 | 0.564 | 0.961 | 0.874 |
| RORA | rs809736 | Intron | C | T | 0.204 | 0.233 | 0.207 | 1.163 | 0.467 | 0.244 | 49 | 50 | 0.980 | 0.920 | 1.028 | 0.850 |
| RORA | rs4775352 | Intron | G | T | 0.106 | 0.097 | 0.120 | 0.784 | 0.372 | 0.104 | 30 | 24 | 1.250 | 0.414 | 0.995 | 0.981 |
| RORA | rs12443239 | Intron | C | T | 0.419 | 0.373 | 0.434 | 0.777 | 0.144 | 0.407 | 69 | 63 | 1.095 | 0.602 | 0.965 | 0.772 |
| RORA | rs782944 | Intron | G | T | 0.493 | 0.447 | 0.519 | 0.747 | 0.087 | 0.463 | 70 | 66 | 1.061 | 0.732 | 0.950 | 0.673 |
| RORA | rs782945 | Intron | C | G | 0.415 | 0.477 | 0.384 | 1.463 | 0.027 | 0.425 | 52 | 64 | 0.813 | 0.265 | 1.049 | 0.707 |
| RORA | rs10519108 | Intron | G | C | 0.317 | 0.283 | 0.314 | 0.864 | 0.430 | 0.307 | 59 | 57 | 1.035 | 0.853 | 0.976 | 0.853 |
| RORA | rs782947 | Intron | G | A | 0.141 | 0.207 | 0.140 | 1.606 | 0.038 | 0.173 | 39 | 38 | 1.026 | 0.909 | 1.114 | 0.505 |
| RORA | rs17303404 | Intron | C | G | 0.060 | 0.063 | 0.054 | 1.178 | 0.651 | 0.052 | 14 | 13 | 1.077 | 0.847 | 1.054 | 0.841 |
| RORA | rs1437550 | Intron | G | T | 0.190 | 0.173 | 0.190 | 0.894 | 0.612 | 0.185 | 45 | 43 | 1.047 | 0.831 | 0.987 | 0.931 |
| RORA | rs12324535 | Intron | C | T | 0.458 | 0.477 | 0.485 | 0.969 | 0.851 | 0.490 | 76 | 67 | 1.134 | 0.452 | 1.021 | 0.861 |
| RORA | rs8037669 | Intron | T | C | 0.345 | 0.367 | 0.330 | 1.178 | 0.358 | 0.355 | 67 | 70 | 0.957 | 0.798 | 1.024 | 0.845 |
| RORA | rs6494246 | Intron | A | G | 0.261 | 0.253 | 0.248 | 1.028 | 0.886 | 0.243 | 52 | 63 | 0.825 | 0.305 | 0.963 | 0.780 |
| RORA | rs2899666 | Intron | G | A | 0.331 | 0.320 | 0.326 | 0.975 | 0.888 | 0.302 | 59 | 57 | 1.035 | 0.853 | 1.002 | 0.990 |
| RORA | rs782934 | Intron | C | G | 0.137 | 0.120 | 0.143 | 0.815 | 0.414 | 0.139 | 27 | 37 | 0.730 | 0.211 | 0.893 | 0.527 |
| RORA | rs7168008 | Intron | A | G | 0.313 | 0.343 | 0.318 | 1.122 | 0.523 | 0.369 | 55 | 63 | 0.873 | 0.462 | 0.997 | 0.979 |
| RORA | rs16943579 | Intron | T | C | 0.067 | 0.083 | 0.070 | 1.212 | 0.549 | 0.071 | 17 | 25 | 0.680 | 0.217 | 0.956 | 0.842 |
| RORA | rs12915830 | Intron | T | C | 0.380 | 0.450 | 0.384 | 1.314 | 0.114 | 0.431 | 78 | 67 | 1.164 | 0.361 | 1.096 | 0.446 |
| RORA | rs2011857 | Intron | C | T | 0.440 | 0.403 | 0.426 | 0.910 | 0.582 | 0.386 | 68 | 65 | 1.046 | 0.795 | 0.989 | 0.928 |
| RORA | rs12903172 | Intron | G | A | 0.380 | 0.393 | 0.368 | 1.112 | 0.543 | 0.375 | 70 | 63 | 1.111 | 0.544 | 1.047 | 0.710 |
| RORA | rs2689352 | Intron | G | A | 0.333 | 0.370 | 0.324 | 1.224 | 0.259 | 0.387 | 67 | 62 | 1.081 | 0.660 | 1.062 | 0.631 |
| RORA | rs940221 | Intron | C | T | 0.250 | 0.267 | 0.252 | 1.080 | 0.692 | 0.235 | 52 | 49 | 1.061 | 0.765 | 1.030 | 0.831 |
| RORA | rs11629597 | Intron | G | A | 0.174 | 0.210 | 0.176 | 1.246 | 0.309 | 0.234 | 49 | 48 | 1.021 | 0.919 | 1.051 | 0.739 |
| RORA | rs7171287 | Intron | C | G | 0.183 | 0.170 | 0.163 | 1.053 | 0.820 | 0.164 | 42 | 34 | 1.235 | 0.359 | 1.058 | 0.727 |
| RORA | rs7177611 | Intron | T | C | 0.296 | 0.263 | 0.298 | 0.840 | 0.357 | 0.281 | 59 | 62 | 0.952 | 0.785 | 0.954 | 0.717 |
| RORA | rs782905 | Intron | T | A | 0.213 | 0.243 | 0.211 | 1.203 | 0.364 | 0.249 | 46 | 46 | 1.000 | 1.000 | 1.042 | 0.778 |
| RORA | rs12899546 | Intron | T | A | 0.489 | 0.517 | 0.488 | 1.120 | 0.505 | 0.519 | 77 | 74 | 0.961 | 0.807 | 1.015 | 0.901 |
| RORA | rs4775355 | Intron | G | A | 0.195 | 0.203 | 0.191 | 1.078 | 0.725 | 0.219 | 49 | 50 | 0.980 | 0.920 | 1.011 | 0.942 |
| RORA | rs782907 | Intron | T | C | 0.310 | 0.313 | 0.314 | 0.997 | 0.987 | 0.326 | 66 | 59 | 1.119 | 0.531 | 1.025 | 0.849 |
| RORA | rs8026340 | Intron | G | A | 0.173 | 0.175 | 0.182 | 0.949 | 0.814 | 0.171 | 39 | 41 | 0.951 | 0.823 | 0.978 | 0.888 |
| RORA | rs782909 | Intron | A | G | 0.151 | 0.160 | 0.147 | 1.103 | 0.678 | 0.156 | 46 | 34 | 1.353 | 0.180 | 1.093 | 0.586 |
| RORA | rs718911 | Intron | C | T | 0.077 | 0.067 | 0.078 | 0.850 | 0.620 | 0.063 | 19 | 13 | 1.462 | 0.289 | 1.037 | 0.881 |
| RORA | rs10519111 | Intron | G | A | 0.173 | 0.153 | 0.163 | 0.931 | 0.760 | 0.135 | 37 | 32 | 1.156 | 0.547 | 1.014 | 0.932 |
| RORA | rs12902142 | Intron | G | A | 0.270 | 0.247 | 0.273 | 0.870 | 0.473 | 0.246 | 48 | 47 | 1.021 | 0.918 | 0.973 | 0.844 |
| RORA | rs782910 | Intron | T | C | 0.243 | 0.240 | 0.233 | 1.042 | 0.837 | 0.259 | 50 | 61 | 0.820 | 0.297 | 0.964 | 0.790 |
| RORA | rs12592311 | Intron | C | G | 0.230 | 0.210 | 0.234 | 0.866 | 0.482 | 0.202 | 40 | 45 | 0.889 | 0.588 | 0.944 | 0.701 |
| RORA | rs893287 | Intron | T | C | 0.396 | 0.391 | 0.366 | 1.112 | 0.554 | 0.405 | 66 | 65 | 1.015 | 0.930 | 1.026 | 0.837 |
| RORA | rs12324440 | Intron | A | G | 0.301 | 0.329 | 0.302 | 1.134 | 0.497 | 0.302 | 52 | 60 | 0.867 | 0.450 | 0.998 | 0.985 |
| RORA | rs782913 | Intron | G | A | 0.366 | 0.318 | 0.353 | 0.854 | 0.382 | 0.329 | 68 | 51 | 1.333 | 0.119 | 1.026 | 0.843 |
| RORA | rs13329643 | Intron | A | G | 0.142 | 0.155 | 0.128 | 1.255 | 0.356 | 0.144 | 41 | 35 | 1.171 | 0.491 | 1.086 | 0.623 |
| RORA | rs782920 | Intron | A | G | 0.049 | 0.057 | 0.062 | 0.909 | 0.789 | 0.060 | 14 | 9 | 1.556 | 0.297 | 1.057 | 0.841 |
| RORA | rs782926 | Intron | T | C | 0.380 | 0.310 | 0.380 | 0.734 | 0.083 | 0.323 | 46 | 58 | 0.793 | 0.239 | 0.888 | 0.368 |
| RORA | rs782928 | Intron | A | G | 0.222 | 0.230 | 0.221 | 1.053 | 0.798 | 0.223 | 46 | 34 | 1.353 | 0.180 | 1.074 | 0.638 |
| RORA | rs782929 | Intron | T | C | 0.056 | 0.060 | 0.062 | 0.965 | 0.921 | 0.062 | 16 | 8 | 2.000 | 0.103 | 1.118 | 0.684 |
| RORA | rs782935 | Intron | G | A | 0.355 | 0.430 | 0.371 | 1.276 | 0.162 | 0.415 | 78 | 66 | 1.182 | 0.317 | 1.093 | 0.463 |
| RORA | rs782938 | Intron | G | A | 0.285 | 0.370 | 0.295 | 1.406 | 0.060 | 0.354 | 69 | 64 | 1.078 | 0.665 | 1.092 | 0.484 |
| RORA | rs16943672 | Intron | A | T | 0.070 | 0.073 | 0.054 | 1.379 | 0.361 | 0.062 | 22 | 15 | 1.467 | 0.250 | 1.166 | 0.527 |
| RORA | rs4775360 | Intron | C | G | 0.440 | 0.390 | 0.473 | 0.713 | 0.049 | 0.380 | 86 | 50 | 1.720 | 0.002 | 1.038 | 0.760 |
| RORA | rs7183068 | Intron | G | T | 0.363 | 0.350 | 0.380 | 0.879 | 0.465 | 0.359 | 55 | 85 | 0.647 | 0.011 | 0.884 | 0.316 |
| RORA | rs1159814 | Intron | G | A | 0.430 | 0.440 | 0.434 | 1.024 | 0.889 | 0.459 | 63 | 71 | 0.887 | 0.490 | 0.980 | 0.866 |
| RORA | rs7170465 | Intron | A | G | 0.204 | 0.213 | 0.236 | 0.876 | 0.514 | 0.256 | 46 | 71 | 0.648 | 0.021 | 0.880 | 0.357 |
| RORA | rs9788704 | Intron | A | G | 0.201 | 0.230 | 0.213 | 1.102 | 0.634 | 0.264 | 43 | 73 | 0.589 | 0.005 | 0.903 | 0.465 |
| RORA | rs11071587 | Intron | T | C | 0.444 | 0.427 | 0.423 | 1.017 | 0.921 | 0.453 | 80 | 60 | 1.333 | 0.091 | 1.069 | 0.583 |
| RORA | rs11071588 | Intron | A | C | 0.404 | 0.377 | 0.402 | 0.898 | 0.536 | 0.359 | 57 | 63 | 0.905 | 0.584 | 0.956 | 0.720 |
| RORA | rs4775362 | Intron | G | A | 0.222 | 0.203 | 0.267 | 0.699 | 0.074 | 0.213 | 41 | 46 | 0.891 | 0.592 | 0.899 | 0.469 |
| RORA | rs1437547 | Intron | C | T | 0.120 | 0.090 | 0.136 | 0.630 | 0.087 | 0.097 | 19 | 23 | 0.826 | 0.537 | 0.861 | 0.464 |
| RORA | rs8024716 | Intron | A | G | 0.215 | 0.180 | 0.252 | 0.652 | 0.039 | 0.225 | 42 | 50 | 0.840 | 0.404 | 0.877 | 0.373 |
| RORA | rs975501 | Intron | G | A | 0.447 | 0.415 | 0.447 | 0.876 | 0.459 | 0.407 | 61 | 55 | 1.109 | 0.578 | 0.992 | 0.948 |
| RORA | rs4238351 | Intron | T | C | 0.408 | 0.370 | 0.419 | 0.816 | 0.241 | 0.367 | 71 | 59 | 1.203 | 0.293 | 0.995 | 0.966 |
| RORA | rs12593790 | Intron | C | T | 0.036 | 0.027 | 0.043 | 0.615 | 0.300 | 0.038 | 7 | 14 | 0.500 | 0.127 | 0.773 | 0.437 |
| RORA | rs17204910 | Intron | G | A | 0.195 | 0.197 | 0.172 | 1.180 | 0.453 | 0.164 | 40 | 36 | 1.111 | 0.646 | 1.061 | 0.709 |
| RORA | rs1437541 | Intron | T | C | 0.060 | 0.067 | 0.050 | 1.346 | 0.416 | 0.097 | 19 | 25 | 0.760 | 0.366 | 0.982 | 0.939 |
| RORA | rs1370433 | Intron | T | A | 0.060 | 0.037 | 0.070 | 0.503 | 0.075 | 0.067 | 13 | 22 | 0.591 | 0.128 | 0.772 | 0.321 |
| RORA | rs17303530 | Intron | C | A | 0.085 | 0.090 | 0.112 | 0.781 | 0.380 | 0.102 | 28 | 25 | 1.120 | 0.680 | 0.973 | 0.890 |
| RORA | rs11071591 | Intron | A | G | 0.419 | 0.410 | 0.423 | 0.950 | 0.766 | 0.435 | 74 | 83 | 0.892 | 0.473 | 0.964 | 0.752 |
| RORA | rs1465812 | Intron | G | A | 0.489 | 0.453 | 0.496 | 0.842 | 0.313 | 0.481 | 68 | 82 | 0.829 | 0.253 | 0.925 | 0.509 |
| RORA | rs4774388 | Intron | G | A | 0.164 | 0.140 | 0.183 | 0.728 | 0.178 | 0.181 | 32 | 37 | 0.865 | 0.547 | 0.904 | 0.548 |
| RORA | rs4775369 | Intron | C | T | 0.430 | 0.463 | 0.419 | 1.199 | 0.289 | 0.425 | 78 | 70 | 1.114 | 0.511 | 1.064 | 0.600 |
| RORA | rs11638592 | Intron | A | T | 0.141 | 0.170 | 0.112 | 1.617 | 0.053 | 0.119 | 35 | 30 | 1.167 | 0.535 | 1.147 | 0.435 |
| RORA | rs1437543 | Intron | T | C | 0.190 | 0.183 | 0.209 | 0.848 | 0.440 | 0.232 | 47 | 60 | 0.783 | 0.209 | 0.914 | 0.530 |
| RORA | rs1370429 | Intron | C | T | 0.366 | 0.337 | 0.403 | 0.752 | 0.105 | 0.415 | 63 | 71 | 0.887 | 0.490 | 0.916 | 0.479 |
| RORA | rs1816624 | Intron | G | A | 0.225 | 0.173 | 0.240 | 0.663 | 0.050 | 0.220 | 49 | 48 | 1.021 | 0.919 | 0.922 | 0.579 |
| RORA | rs4774390 | Intron | C | G | 0.338 | 0.347 | 0.333 | 1.061 | 0.740 | 0.346 | 77 | 68 | 1.132 | 0.455 | 1.042 | 0.738 |
| RORA | rs4775371 | Intron | T | C | 0.145 | 0.164 | 0.159 | 1.042 | 0.860 | 0.184 | 46 | 44 | 1.045 | 0.833 | 1.019 | 0.905 |
| RORA | rs17204938 | Intron | G | T | 0.011 | 0.040 | 0.020 | 2.092 | 0.162 | 0.039 | 12 | 7 | 1.714 | 0.251 | 1.312 | 0.446 |
| RORA | rs7167741 | Intron | A | T | 0.493 | 0.483 | 0.485 | 0.995 | 0.978 | 0.446 | 74 | 74 | 1.000 | 1.000 | 0.999 | 0.993 |
| RORA | rs17204952 | Intron | A | G | 0.123 | 0.143 | 0.140 | 1.032 | 0.898 | 0.175 | 46 | 36 | 1.278 | 0.270 | 1.066 | 0.695 |
| RORA | rs17204959 | Intron | A | G | 0.489 | 0.467 | 0.488 | 0.917 | 0.609 | 0.456 | 70 | 69 | 1.014 | 0.932 | 0.984 | 0.895 |
| RORA | rs4774392 | Intron | C | G | 0.277 | 0.284 | 0.306 | 0.903 | 0.587 | 0.306 | 65 | 56 | 1.161 | 0.413 | 1.012 | 0.927 |
| RORA | rs726914 | Intron | T | C | 0.368 | 0.372 | 0.345 | 1.123 | 0.514 | 0.337 | 66 | 62 | 1.065 | 0.724 | 1.040 | 0.757 |
| RORA | rs726913 | Intron | T | C | 0.396 | 0.409 | 0.383 | 1.118 | 0.524 | 0.367 | 55 | 56 | 0.982 | 0.924 | 1.023 | 0.860 |
| RORA | rs726955 | Intron | T | C | 0.232 | 0.257 | 0.221 | 1.218 | 0.325 | 0.262 | 58 | 56 | 1.036 | 0.851 | 1.049 | 0.724 |
| RORA | rs2118326 | Intron | A | G | 0.234 | 0.193 | 0.238 | 0.766 | 0.198 | 0.240 | 49 | 47 | 1.043 | 0.838 | 0.953 | 0.743 |
| RORA | rs4775374 | Intron | C | T | 0.377 | 0.377 | 0.364 | 1.054 | 0.764 | 0.388 | 63 | 61 | 1.033 | 0.858 | 1.019 | 0.883 |
| RORA | rs1550226 | Intron | A | G | 0.113 | 0.113 | 0.124 | 0.903 | 0.696 | 0.167 | 41 | 34 | 1.206 | 0.419 | 1.027 | 0.880 |
| RORA | rs930358 | Intron | C | T | 0.149 | 0.140 | 0.165 | 0.821 | 0.417 | 0.183 | 36 | 43 | 0.837 | 0.431 | 0.922 | 0.624 |
| RORA | rs930359 | Intron | G | A | 0.243 | 0.233 | 0.225 | 1.049 | 0.811 | 0.242 | 51 | 57 | 0.895 | 0.564 | 0.985 | 0.912 |
| RORA | rs7175393 | Intron | A | G | 0.183 | 0.198 | 0.151 | 1.386 | 0.149 | 0.197 | 37 | 53 | 0.698 | 0.092 | 0.985 | 0.921 |
| RORA | rs17303572 | Intron | A | G | 0.352 | 0.360 | 0.345 | 1.068 | 0.711 | 0.373 | 53 | 78 | 0.680 | 0.029 | 0.933 | 0.581 |
| RORA | rs1002147 | Intron | C | G | 0.113 | 0.123 | 0.128 | 0.959 | 0.871 | 0.146 | 29 | 34 | 0.853 | 0.529 | 0.957 | 0.807 |
| RORA | rs10438343 | Promoter | A | G | 0.173 | 0.190 | 0.147 | 1.358 | 0.181 | 0.207 | 41 | 53 | 0.774 | 0.216 | 0.999 | 0.994 |
| RORA | rs7177878 | Promoter | T | C | 0.332 | 0.287 | 0.323 | 0.843 | 0.356 | 0.306 | 52 | 58 | 0.897 | 0.567 | 0.941 | 0.645 |
| RORB | rs4090240 | Intron | T | C | 0.232 | 0.298 | 0.204 | 1.655 | 0.013 | 0.276 | 60 | 44 | 1.364 | 0.117 | 1.192 | 0.217 |
| RORB | rs17227876 | Intron | A | T | 0.305 | 0.272 | 0.287 | 0.926 | 0.684 | 0.248 | 42 | 49 | 0.857 | 0.463 | 0.953 | 0.730 |
| RORB | rs10869410 | Intron | G | A | 0.383 | 0.346 | 0.409 | 0.762 | 0.123 | 0.402 | 57 | 62 | 0.919 | 0.647 | 0.924 | 0.535 |
| RORB | rs13293006 | Intron | A | C | 0.450 | 0.433 | 0.425 | 1.032 | 0.856 | 0.379 | 55 | 62 | 0.887 | 0.518 | 0.983 | 0.894 |
| RORB | rs1018584 | Intron | A | C | 0.309 | 0.379 | 0.298 | 1.442 | 0.044 | 0.347 | 66 | 50 | 1.320 | 0.137 | 1.151 | 0.283 |
| RORB | rs10869412 | Intron | T | C | 0.243 | 0.183 | 0.276 | 0.590 | 0.010 | 0.272 | 47 | 57 | 0.825 | 0.327 | 0.858 | 0.280 |
| RORB | rs7857053 | Intron | C | T | 0.323 | 0.290 | 0.315 | 0.890 | 0.534 | 0.279 | 48 | 56 | 0.857 | 0.433 | 0.943 | 0.667 |
| RORB | rs10869418 | Intron | T | A | 0.221 | 0.164 | 0.254 | 0.578 | 0.010 | 0.234 | 47 | 49 | 0.959 | 0.838 | 0.884 | 0.402 |
| RORB | rs17611535 | Intron | T | C | 0.146 | 0.170 | 0.142 | 1.240 | 0.362 | 0.131 | 31 | 28 | 1.107 | 0.696 | 1.074 | 0.685 |
| RORB | rs10217594 | Intron | T | C | 0.264 | 0.313 | 0.254 | 1.341 | 0.122 | 0.283 | 53 | 45 | 1.178 | 0.419 | 1.106 | 0.466 |
| RORB | rs10781235 | Intron | T | C | 0.215 | 0.163 | 0.250 | 0.586 | 0.011 | 0.227 | 55 | 48 | 1.146 | 0.490 | 0.927 | 0.600 |
| RORB | rs7037043 | Intron | G | A | 0.349 | 0.310 | 0.383 | 0.724 | 0.072 | 0.342 | 61 | 58 | 1.052 | 0.783 | 0.941 | 0.635 |
| RORB | rs17684881 | Intron | T | C | 0.085 | 0.077 | 0.094 | 0.803 | 0.471 | 0.084 | 20 | 25 | 0.800 | 0.456 | 0.908 | 0.653 |
| RORB | rs17612218 | Intron | A | G | 0.123 | 0.117 | 0.117 | 0.995 | 0.985 | 0.100 | 21 | 24 | 0.875 | 0.655 | 0.974 | 0.893 |
| RORB | rs11144020 | Intron | A | C | 0.493 | 0.436 | 0.528 | 0.691 | 0.032 | 0.510 | 80 | 63 | 1.270 | 0.155 | 0.975 | 0.833 |
| RORB | rs17612778 | Intron | A | C | 0.371 | 0.342 | 0.378 | 0.857 | 0.384 | 0.326 | 58 | 68 | 0.853 | 0.373 | 0.934 | 0.589 |
| RORB | rs17612874 | Intron | A | C | 0.136 | 0.120 | 0.138 | 0.853 | 0.532 | 0.142 | 35 | 29 | 1.207 | 0.453 | 1.007 | 0.967 |
| RORB | rs17691363 | Intron | G | A | 0.032 | 0.023 | 0.035 | 0.656 | 0.406 | 0.040 | 11 | 11 | 1.000 | 1.000 | 0.928 | 0.818 |
| RORB | rs10869430 | Intron | A | G | 0.365 | 0.351 | 0.391 | 0.845 | 0.340 | 0.375 | 64 | 47 | 1.362 | 0.107 | 1.022 | 0.866 |
| RORB | rs1157358 | Intron | T | C | 0.137 | 0.218 | 0.091 | 2.802 | 4.5E-05 | 0.167 | 29 | 38 | 0.763 | 0.272 | 1.162 | 0.401 |
| RORB | rs7022435 | Intron | A | G | 0.176 | 0.310 | 0.135 | 2.881 | 1.1E-06 | 0.203 | 34 | 51 | 0.667 | 0.065 | 1.150 | 0.373 |
| RORB | rs17691614 | Intron | G | A | 0.273 | 0.238 | 0.276 | 0.822 | 0.316 | 0.232 | 44 | 46 | 0.957 | 0.833 | 0.947 | 0.703 |
| RORB | rs7032677 | Intron | A | G | 0.461 | 0.560 | 0.429 | 1.693 | 0.002 | 0.443 | 54 | 79 | 0.684 | 0.030 | 1.037 | 0.765 |
| RORB | rs3750420 | Intron | T | C | 0.237 | 0.375 | 0.201 | 2.388 | 7.9E-06 | 0.304 | 65 | 66 | 0.985 | 0.930 | 1.176 | 0.214 |
| RORB | rs1570502 | Intron | T | C | 0.395 | 0.335 | 0.413 | 0.715 | 0.059 | 0.404 | 62 | 46 | 1.348 | 0.124 | 0.980 | 0.876 |
| RORB | rs1013078 | Intron | T | A | 0.338 | 0.423 | 0.324 | 1.530 | 0.016 | 0.365 | 50 | 71 | 0.704 | 0.056 | 1.023 | 0.858 |
| RORB | rs11144033 | Intron | G | A | 0.404 | 0.490 | 0.386 | 1.529 | 0.014 | 0.402 | 53 | 76 | 0.697 | 0.043 | 1.020 | 0.875 |
| RORB | rs3903529 | Intron | A | T | 0.229 | 0.357 | 0.205 | 2.154 | 8.2E-05 | 0.247 | 34 | 55 | 0.618 | 0.026 | 1.094 | 0.537 |
| RORB | rs968357 | Intron | C | T | 0.197 | 0.177 | 0.195 | 0.884 | 0.573 | 0.197 | 44 | 43 | 1.023 | 0.915 | 0.979 | 0.890 |
| RORB | rs11144037 | Intron | A | G | 0.130 | 0.193 | 0.109 | 1.952 | 0.006 | 0.131 | 29 | 34 | 0.853 | 0.529 | 1.121 | 0.519 |
| RORB | rs12352112 | Intron | T | C | 0.061 | 0.037 | 0.051 | 0.706 | 0.403 | 0.046 | 14 | 10 | 1.400 | 0.414 | 0.999 | 0.997 |
| RORB | rs11144039 | Intron | C | T | 0.058 | 0.057 | 0.055 | 1.037 | 0.922 | 0.085 | 28 | 16 | 1.750 | 0.070 | 1.160 | 0.535 |
| RORB | rs11144043 | Intron | C | G | 0.201 | 0.150 | 0.227 | 0.602 | 0.021 | 0.199 | 40 | 43 | 0.930 | 0.742 | 0.882 | 0.419 |
| RORB | rs7865407 | Intron | G | T | 0.394 | 0.367 | 0.413 | 0.822 | 0.261 | 0.410 | 81 | 57 | 1.421 | 0.041 | 1.036 | 0.776 |
| RORB | rs10869435 | Intron | A | T | 0.454 | 0.490 | 0.437 | 1.240 | 0.210 | 0.469 | 57 | 87 | 0.655 | 0.012 | 0.955 | 0.703 |
| RORB | rs12001830 | Intron | T | C | 0.193 | 0.170 | 0.221 | 0.724 | 0.134 | 0.171 | 43 | 44 | 0.977 | 0.915 | 0.928 | 0.623 |
| RORB | rs10121918 | Intron | A | G | 0.393 | 0.417 | 0.377 | 1.180 | 0.352 | 0.436 | 91 | 59 | 1.542 | 0.009 | 1.143 | 0.273 |
| RORB | rs7033059 | Intron | A | G | 0.338 | 0.293 | 0.371 | 0.704 | 0.052 | 0.330 | 56 | 69 | 0.812 | 0.245 | 0.886 | 0.341 |
| RORB | rs11144053 | Intron | C | G | 0.229 | 0.280 | 0.197 | 1.587 | 0.023 | 0.227 | 37 | 55 | 0.673 | 0.061 | 1.023 | 0.879 |
| RORB | rs1327836 | Intron | G | T | 0.155 | 0.124 | 0.165 | 0.716 | 0.168 | 0.163 | 28 | 41 | 0.683 | 0.118 | 0.856 | 0.369 |
| RORB | rs17060408 | Intron | C | T | 0.058 | 0.047 | 0.059 | 0.785 | 0.526 | 0.085 | 15 | 22 | 0.682 | 0.250 | 0.870 | 0.579 |
| RORB | rs1410227 | 3' UTR | G | A | 0.119 | 0.102 | 0.130 | 0.761 | 0.308 | 0.137 | 23 | 41 | 0.561 | 0.024 | 0.830 | 0.318 |
| RORB | rs1410225 | 3' UTR | G | A | 0.194 | 0.173 | 0.211 | 0.784 | 0.261 | 0.207 | 36 | 55 | 0.655 | 0.046 | 0.865 | 0.340 |
| MAF = Minor Allele Frequency | | | | | | | | | | | | | | | | |
| OR = Odds Ratio of the minor allele | | | | | | | | | | | | | | | | |
| T = Number of transmitted of minor alleles; U = Number of untransmitted of minor alleles | | | | | | | | | | | | | | | | |
