# Supplementary material for: Evidence for genetic association of RORB with bipolar disorder
Source: BMC Psychiatry. 2009 Nov 12;9:70. doi: 10.1186/1471-244X-9-70 (PMC2780413; doi:10.1186/1471-244X-9-70)
Supplement: Additional file 3 — Association results for age-at-onset quantitative trait analysis. This table contains the results of the quantitative trait association analyses of RORA and RORB SNPs with age-at-onset of bipolar disorder. [file 1471-244X-9-70-S3.doc]

Association results for age-at-onset quantitative trait analysis

| **Gene** | **SNP** | **Beta**  **(test statistic)** | **Permuted *P-*value** |
| --- | --- | --- | --- |
| RORA | rs3743266 | -0.302 | 0.618 |
| RORA | rs7165874 | -0.402 | 0.481 |
| RORA | rs1869488 | -0.004 | 0.995 |
| RORA | rs17270167 | 0.999 | 0.179 |
| RORA | rs1866006 | 0.555 | 0.556 |
| RORA | rs17270188 | 0.636 | 0.334 |
| RORA | rs17191442 | -0.752 | 0.368 |
| RORA | rs1866007 | -0.760 | 0.199 |
| RORA | rs17191463 | 0.522 | 0.542 |
| RORA | rs4594196 | -0.417 | 0.547 |
| RORA | rs3905275 | -0.893 | 0.201 |
| RORA | rs17270216 | -0.277 | 0.667 |
| RORA | rs10851684 | -0.405 | 0.444 |
| RORA | rs12439380 | -1.102 | 0.102 |
| RORA | rs10431795 | 1.177 | 0.034 |
| RORA | rs10851685 | -1.487 | 0.056 |
| RORA | rs974828 | 0.417 | 0.687 |
| RORA | rs8036966 | -0.556 | 0.531 |
| RORA | rs11633266 | 0.052 | 0.926 |
| RORA | rs340002 | -0.774 | 0.234 |
| RORA | rs11632600 | -0.761 | 0.295 |
| RORA | rs2289163 | -0.793 | 0.497 |
| RORA | rs7172348 | 0.821 | 0.228 |
| RORA | rs340009 | 0.968 | 0.117 |
| RORA | rs340021 | 0.751 | 0.553 |
| RORA | rs16942816 | -0.856 | 0.418 |
| RORA | rs340023 | 1.041 | 0.109 |
| RORA | rs103946 | -0.255 | 0.755 |
| RORA | rs890156 | 0.183 | 0.736 |
| RORA | rs235512 | -0.099 | 0.850 |
| RORA | rs11630262 | -0.248 | 0.716 |
| RORA | rs339976 | 0.465 | 0.513 |
| RORA | rs339996 | 0.130 | 0.811 |
| RORA | rs17191554 | 1.434 | 0.130 |
| RORA | rs9806453 | 0.062 | 0.931 |
| RORA | rs339998 | -0.644 | 0.244 |
| RORA | rs2438062 | 0.787 | 0.229 |
| RORA | rs2433026 | 0.373 | 0.534 |
| RORA | rs1364822 | -0.217 | 0.786 |
| RORA | rs17303097 | -0.764 | 0.231 |
| RORA | rs880626 | -0.769 | 0.190 |
| RORA | rs880625 | -1.112 | 0.152 |
| RORA | rs12916690 | 0.299 | 0.685 |
| RORA | rs4775287 | 0.182 | 0.792 |
| RORA | rs8040332 | 0.624 | 0.447 |
| RORA | rs919000 | 0.554 | 0.461 |
| RORA | rs11629812 | 1.014 | 0.159 |
| RORA | rs6494217 | 1.016 | 0.145 |
| RORA | rs1425287 | 0.734 | 0.343 |
| RORA | rs13329238 | 0.292 | 0.574 |
| RORA | rs4774371 | 0.472 | 0.442 |
| RORA | rs7181662 | 0.522 | 0.348 |
| RORA | rs8027032 | -0.764 | 0.305 |
| RORA | rs8038077 | -0.878 | 0.346 |
| RORA | rs1030347 | 0.553 | 0.336 |
| RORA | rs17303111 | -0.947 | 0.147 |
| RORA | rs7173461 | 0.149 | 0.803 |
| RORA | rs1863270 | 0.658 | 0.253 |
| RORA | rs6494219 | -1.104 | 0.095 |
| RORA | rs17191582 | -0.279 | 0.749 |
| RORA | rs6494221 | -0.512 | 0.497 |
| RORA | rs8043356 | -0.598 | 0.474 |
| RORA | rs12899193 | -0.803 | 0.174 |
| RORA | rs12909890 | -0.688 | 0.272 |
| RORA | rs16943012 | 0.884 | 0.486 |
| RORA | rs8024629 | -0.180 | 0.804 |
| RORA | rs1820357 | -0.557 | 0.415 |
| RORA | rs11635314 | 0.102 | 0.903 |
| RORA | rs341413 | -0.188 | 0.819 |
| RORA | rs17237367 | -0.789 | 0.309 |
| RORA | rs17191596 | 0.301 | 0.713 |
| RORA | rs9920962 | 0.296 | 0.728 |
| RORA | rs716593 | -0.283 | 0.864 |
| RORA | rs8025324 | 0.600 | 0.471 |
| RORA | rs1871858 | 2.033 | 0.121 |
| RORA | rs7183955 | 0.342 | 0.639 |
| RORA | rs17204402 | -0.865 | 0.216 |
| RORA | rs1563565 | -0.778 | 0.237 |
| RORA | rs8040151 | -0.664 | 0.315 |
| RORA | rs17204440 | -0.429 | 0.475 |
| RORA | rs7164773 | 0.735 | 0.259 |
| RORA | rs12438866 | -0.643 | 0.342 |
| RORA | rs10519068 | 0.779 | 0.381 |
| RORA | rs10519070 | -0.232 | 0.785 |
| RORA | rs922782 | -0.429 | 0.498 |
| RORA | rs7173227 | 0.148 | 0.858 |
| RORA | rs12324380 | 0.065 | 0.976 |
| RORA | rs2899662 | 0.176 | 0.768 |
| RORA | rs1680446 | -1.173 | 0.567 |
| RORA | rs17204475 | 1.169 | 0.179 |
| RORA | rs4775297 | 0.063 | 0.910 |
| RORA | rs16943117 | 0.068 | 0.925 |
| RORA | rs11632858 | 0.277 | 0.848 |
| RORA | rs341459 | 0.024 | 0.974 |
| RORA | rs10162630 | 0.080 | 0.882 |
| RORA | rs17204489 | 0.066 | 0.968 |
| RORA | rs12902540 | -0.226 | 0.792 |
| RORA | rs17204496 | -0.832 | 0.593 |
| RORA | rs10519076 | -0.543 | 0.522 |
| RORA | rs7169281 | -0.212 | 0.757 |
| RORA | rs341380 | -0.087 | 0.894 |
| RORA | rs17270501 | -1.604 | 0.424 |
| RORA | rs12439995 | 0.701 | 0.362 |
| RORA | rs12440095 | 0.675 | 0.459 |
| RORA | rs341390 | 0.437 | 0.543 |
| RORA | rs7175883 | 1.298 | 0.463 |
| RORA | rs341392 | -0.305 | 0.654 |
| RORA | rs6494229 | 0.433 | 0.475 |
| RORA | rs7181803 | 1.298 | 0.479 |
| RORA | rs8041061 | 0.242 | 0.710 |
| RORA | rs8042149 | -0.203 | 0.732 |
| RORA | rs4775301 | 0.141 | 0.814 |
| RORA | rs341398 | 0.428 | 0.482 |
| RORA | rs341399 | 0.577 | 0.494 |
| RORA | rs341400 | 0.382 | 0.761 |
| RORA | rs3959689 | 0.377 | 0.546 |
| RORA | rs11630062 | -0.269 | 0.783 |
| RORA | rs341403 | -0.373 | 0.531 |
| RORA | rs754499 | 0.273 | 0.675 |
| RORA | rs11630227 | -0.124 | 0.856 |
| RORA | rs341408 | -0.008 | 0.987 |
| RORA | rs17204545 | -0.848 | 0.323 |
| RORA | rs11631055 | 0.704 | 0.419 |
| RORA | rs1673336 | -0.218 | 0.761 |
| RORA | rs2607582 | 0.020 | 0.982 |
| RORA | rs2306502 | -0.789 | 0.378 |
| RORA | rs2306500 | -0.071 | 0.928 |
| RORA | rs10519085 | -0.143 | 0.844 |
| RORA | rs8041466 | -0.239 | 0.694 |
| RORA | rs1482058 | -0.251 | 0.633 |
| RORA | rs1902618 | 0.468 | 0.527 |
| RORA | rs7166448 | 1.404 | 0.152 |
| RORA | rs341365 | -0.205 | 0.756 |
| RORA | rs17204573 | 0.137 | 0.900 |
| RORA | rs4775310 | -0.762 | 0.405 |
| RORA | rs341387 | 0.641 | 0.289 |
| RORA | rs8027829 | -0.684 | 0.256 |
| RORA | rs11631432 | 0.418 | 0.484 |
| RORA | rs4775311 | 0.508 | 0.499 |
| RORA | rs8039843 | 0.054 | 0.956 |
| RORA | rs113168 | 0.204 | 0.732 |
| RORA | rs12913421 | 0.090 | 0.863 |
| RORA | rs11071564 | 0.724 | 0.255 |
| RORA | rs7172917 | -0.379 | 0.592 |
| RORA | rs12592999 | 0.395 | 0.523 |
| RORA | rs4774376 | -0.874 | 0.298 |
| RORA | rs7168782 | -0.611 | 0.414 |
| RORA | rs17270578 | -0.523 | 0.594 |
| RORA | rs2899663 | -0.213 | 0.727 |
| RORA | rs2414686 | -0.072 | 0.910 |
| RORA | rs17204628 | -0.289 | 0.778 |
| RORA | rs17204635 | -3.088 | 0.177 |
| RORA | rs17270599 | -0.584 | 0.544 |
| RORA | rs2899664 | -0.470 | 0.382 |
| RORA | rs2062091 | 0.353 | 0.537 |
| RORA | rs11638433 | 0.576 | 0.349 |
| RORA | rs8027234 | -0.378 | 0.641 |
| RORA | rs4378570 | 0.075 | 0.906 |
| RORA | rs4775314 | 0.970 | 0.106 |
| RORA | rs16943311 | 0.308 | 0.642 |
| RORA | rs6494232 | -1.041 | 0.343 |
| RORA | rs8036866 | 0.036 | 0.977 |
| RORA | rs16943318 | -0.350 | 0.573 |
| RORA | rs4775318 | 1.024 | 0.110 |
| RORA | rs8033151 | -0.751 | 0.183 |
| RORA | rs2062094 | 0.913 | 0.164 |
| RORA | rs7166062 | -1.090 | 0.311 |
| RORA | rs11854760 | -0.411 | 0.519 |
| RORA | rs11634318 | 0.196 | 0.858 |
| RORA | rs2279291 | 0.271 | 0.657 |
| RORA | rs12442938 | 0.505 | 0.545 |
| RORA | rs1351546 | 0.693 | 0.271 |
| RORA | rs1351545 | -0.933 | 0.183 |
| RORA | rs7342684 | 0.251 | 0.725 |
| RORA | rs12438355 | 1.030 | 0.137 |
| RORA | rs4775330 | -0.244 | 0.711 |
| RORA | rs12442730 | 0.712 | 0.336 |
| RORA | rs4332688 | 0.448 | 0.455 |
| RORA | rs2140441 | -0.410 | 0.478 |
| RORA | rs17204698 | -0.953 | 0.198 |
| RORA | rs1523527 | 0.267 | 0.626 |
| RORA | rs12440921 | 0.918 | 0.397 |
| RORA | rs8034880 | 1.547 | 0.075 |
| RORA | rs8034950 | 0.726 | 0.221 |
| RORA | rs12912233 | 0.067 | 0.889 |
| RORA | rs17237521 | 1.751 | 0.038 |
| RORA | rs2414687 | 0.105 | 0.874 |
| RORA | rs6494237 | -0.140 | 0.809 |
| RORA | rs4638514 | -0.184 | 0.763 |
| RORA | rs11071577 | -0.180 | 0.744 |
| RORA | rs1589702 | 0.294 | 0.630 |
| RORA | rs7180208 | -0.121 | 0.843 |
| RORA | rs7495128 | 0.025 | 0.972 |
| RORA | rs7178442 | 0.136 | 0.865 |
| RORA | rs16943448 | 0.091 | 0.925 |
| RORA | rs7176717 | 0.617 | 0.298 |
| RORA | rs12591914 | -0.109 | 0.879 |
| RORA | rs16943453 | -0.410 | 0.586 |
| RORA | rs1110418 | -0.146 | 0.809 |
| RORA | rs4265751 | -0.432 | 0.469 |
| RORA | rs951265 | -0.008 | 0.993 |
| RORA | rs1916645 | 0.544 | 0.606 |
| RORA | rs10519097 | 1.438 | 0.050 |
| RORA | rs17204770 | -0.245 | 0.648 |
| RORA | rs12908671 | 0.274 | 0.780 |
| RORA | rs16943472 | 0.376 | 0.648 |
| RORA | rs11638929 | -0.432 | 0.436 |
| RORA | rs10519099 | 0.560 | 0.320 |
| RORA | rs10519100 | -0.368 | 0.679 |
| RORA | rs17237563 | 0.623 | 0.412 |
| RORA | rs1523530 | 0.404 | 0.607 |
| RORA | rs16943489 | 1.024 | 0.240 |
| RORA | rs17237570 | 0.856 | 0.424 |
| RORA | rs8040930 | 0.655 | 0.647 |
| RORA | rs4774381 | -0.055 | 0.947 |
| RORA | rs1403737 | 0.665 | 0.352 |
| RORA | rs10519105 | -0.191 | 0.839 |
| RORA | rs1403739 | 1.002 | 0.186 |
| RORA | rs17270745 | 0.896 | 0.307 |
| RORA | rs782957 | -0.318 | 0.733 |
| RORA | rs10519107 | 0.307 | 0.591 |
| RORA | rs7162388 | -0.853 | 0.479 |
| RORA | rs809736 | -0.624 | 0.364 |
| RORA | rs4775352 | 1.416 | 0.147 |
| RORA | rs12443239 | -0.503 | 0.386 |
| RORA | rs782944 | -0.639 | 0.267 |
| RORA | rs782945 | 0.241 | 0.660 |
| RORA | rs10519108 | -1.457 | 0.026 |
| RORA | rs782947 | -0.774 | 0.310 |
| RORA | rs17303404 | -0.707 | 0.547 |
| RORA | rs1437550 | 0.033 | 0.957 |
| RORA | rs12324535 | -0.602 | 0.264 |
| RORA | rs8037669 | -1.230 | 0.036 |
| RORA | rs6494246 | -0.656 | 0.299 |
| RORA | rs2899666 | 0.374 | 0.558 |
| RORA | rs782934 | -1.517 | 0.070 |
| RORA | rs7168008 | -0.300 | 0.607 |
| RORA | rs16943579 | 1.267 | 0.218 |
| RORA | rs12915830 | 1.099 | 0.049 |
| RORA | rs2011857 | -0.408 | 0.497 |
| RORA | rs12903172 | 0.966 | 0.112 |
| RORA | rs2689352 | 0.605 | 0.312 |
| RORA | rs940221 | 0.450 | 0.536 |
| RORA | rs11629597 | -1.009 | 0.119 |
| RORA | rs7171287 | 1.168 | 0.139 |
| RORA | rs7177611 | -0.269 | 0.660 |
| RORA | rs782905 | -1.111 | 0.121 |
| RORA | rs12899546 | -0.070 | 0.883 |
| RORA | rs4775355 | -0.400 | 0.607 |
| RORA | rs782907 | -0.790 | 0.180 |
| RORA | rs8026340 | -0.273 | 0.718 |
| RORA | rs4774386 | 0.716 | 0.377 |
| RORA | rs782909 | -0.290 | 0.714 |
| RORA | rs718911 | 0.390 | 0.713 |
| RORA | rs10519111 | 0.897 | 0.228 |
| RORA | rs12902142 | 0.148 | 0.822 |
| RORA | rs782910 | -0.351 | 0.585 |
| RORA | rs12592311 | -0.225 | 0.749 |
| RORA | rs893287 | -0.594 | 0.311 |
| RORA | rs12324440 | 0.642 | 0.315 |
| RORA | rs782913 | 0.037 | 0.950 |
| RORA | rs13329643 | -0.048 | 0.946 |
| RORA | rs782920 | -2.064 | 0.097 |
| RORA | rs782926 | -0.260 | 0.687 |
| RORA | rs782928 | -0.407 | 0.530 |
| RORA | rs782929 | -2.221 | 0.063 |
| RORA | rs782935 | -0.280 | 0.617 |
| RORA | rs782938 | 0.352 | 0.547 |
| RORA | rs16943672 | 0.384 | 0.691 |
| RORA | rs4775360 | -0.029 | 0.967 |
| RORA | rs7183068 | -0.345 | 0.561 |
| RORA | rs1159814 | 0.112 | 0.864 |
| RORA | rs7170465 | 0.149 | 0.818 |
| RORA | rs9788704 | 0.138 | 0.824 |
| RORA | rs11071587 | 0.137 | 0.802 |
| RORA | rs11071588 | 0.378 | 0.542 |
| RORA | rs4775362 | -1.227 | 0.118 |
| RORA | rs1437547 | -0.860 | 0.437 |
| RORA | rs8024716 | -1.058 | 0.125 |
| RORA | rs975501 | -0.436 | 0.453 |
| RORA | rs4238351 | -0.694 | 0.234 |
| RORA | rs12593790 | 1.864 | 0.283 |
| RORA | rs17204910 | 0.464 | 0.552 |
| RORA | rs1437541 | 1.134 | 0.244 |
| RORA | rs1370433 | 0.342 | 0.815 |
| RORA | rs17303530 | -1.436 | 0.140 |
| RORA | rs11071591 | 0.287 | 0.609 |
| RORA | rs1465812 | 0.030 | 0.947 |
| RORA | rs4774388 | 0.009 | 0.984 |
| RORA | rs4775369 | -0.185 | 0.766 |
| RORA | rs11638592 | 1.096 | 0.200 |
| RORA | rs1437543 | 0.013 | 0.979 |
| RORA | rs1370429 | -0.286 | 0.616 |
| RORA | rs1816624 | -0.639 | 0.365 |
| RORA | rs4774390 | -0.094 | 0.881 |
| RORA | rs4775371 | -0.625 | 0.436 |
| RORA | rs17204938 | -1.665 | 0.263 |
| RORA | rs7167741 | -0.262 | 0.682 |
| RORA | rs17204952 | -0.863 | 0.300 |
| RORA | rs17204959 | -0.018 | 0.984 |
| RORA | rs4774392 | 0.026 | 0.960 |
| RORA | rs726914 | 0.248 | 0.672 |
| RORA | rs726913 | 0.073 | 0.896 |
| RORA | rs726955 | -0.065 | 0.917 |
| RORA | rs2118326 | 0.472 | 0.520 |
| RORA | rs4775374 | 0.723 | 0.221 |
| RORA | rs1550226 | 0.088 | 0.926 |
| RORA | rs930358 | -1.665 | 0.050 |
| RORA | rs930359 | 0.467 | 0.419 |
| RORA | rs7175393 | 1.541 | 0.025 |
| RORA | rs17303572 | 0.262 | 0.670 |
| RORA | rs1002147 | -1.068 | 0.222 |
| RORA | rs10438343 | 1.053 | 0.103 |
| RORA | rs7177878 | 0.232 | 0.672 |
| RORB | rs4090240 | 0.532 | 0.464 |
| RORB | rs17227876 | -0.318 | 0.665 |
| RORB | rs10869410 | -0.161 | 0.769 |
| RORB | rs13293006 | -0.274 | 0.660 |
| RORB | rs1018584 | 0.311 | 0.642 |
| RORB | rs10869412 | -0.012 | 0.981 |
| RORB | rs7857053 | -0.283 | 0.689 |
| RORB | rs10869418 | -0.185 | 0.767 |
| RORB | rs17611535 | -0.013 | 0.989 |
| RORB | rs10217594 | -0.351 | 0.548 |
| RORB | rs10781235 | 0.168 | 0.827 |
| RORB | rs7037043 | 0.246 | 0.689 |
| RORB | rs17684881 | 0.743 | 0.445 |
| RORB | rs17612218 | 1.702 | 0.092 |
| RORB | rs11144020 | 0.226 | 0.690 |
| RORB | rs17612778 | -0.368 | 0.581 |
| RORB | rs17612874 | 0.306 | 0.717 |
| RORB | rs17691363 | 1.362 | 0.442 |
| RORB | rs10869430 | -0.409 | 0.517 |
| RORB | rs1157358 | 0.832 | 0.357 |
| RORB | rs7022435 | 0.201 | 0.793 |
| RORB | rs17691614 | 0.497 | 0.537 |
| RORB | rs7032677 | 0.627 | 0.300 |
| RORB | rs3750420 | 0.122 | 0.869 |
| RORB | rs1570502 | -0.596 | 0.298 |
| RORB | rs1013078 | 0.213 | 0.742 |
| RORB | rs11144033 | 0.172 | 0.816 |
| RORB | rs3903529 | 0.258 | 0.710 |
| RORB | rs968357 | -0.324 | 0.647 |
| RORB | rs11144037 | 0.531 | 0.547 |
| RORB | rs12352112 | -0.277 | 0.840 |
| RORB | rs11144039 | -0.029 | 0.977 |
| RORB | rs11144043 | -0.498 | 0.506 |
| RORB | rs7865407 | 0.065 | 0.913 |
| RORB | rs10869435 | 0.061 | 0.920 |
| RORB | rs12001830 | -1.725 | 0.037 |
| RORB | rs10121918 | 0.130 | 0.825 |
| RORB | rs7033059 | -1.399 | 0.039 |
| RORB | rs11144053 | 1.234 | 0.119 |
| RORB | rs1327836 | -0.451 | 0.635 |
| RORB | rs17060408 | -1.441 | 0.256 |
| RORB | rs1410227 | -0.774 | 0.466 |
| RORB | rs1410225 | -1.100 | 0.148 |
