# Supplementary material for: Evidence for genetic association of RORB with bipolar disorder
Source: BMC Psychiatry. 2009 Nov 12;9:70. doi: 10.1186/1471-244X-9-70 (PMC2780413; doi:10.1186/1471-244X-9-70)
Supplement: Additional file 4 — RORB SNPs associated with bipolar disorder in genome-wide association studies. This table contains the P-values of RORB SNPs associated with bipolar disorder in our study and four genome-wide association analyses. [file 1471-244X-9-70-S4.doc]

*RORB* SNPs associated with bipolar disorder in four genome-wide association studies

A blank indicates that the specified SNP was not tested in the sample.

CC sample: results from case-control sample, this study

WTCC: Wellcome Trust Case Control sample [42]

NIMH: NIMH Genetics Initiative sample [43]

German: German sample [43]

STEP-BD: Systematic Treatment Enhancement Program for Bipolar Disorder [41]

Citations refer to references found in the companion article
